# Supplementary material for: Isotope systematics of subfossil, historical, and modern Nautilus macromphalus from New Caledonia
Source: PLoS One. 2022 Dec 28;17(12):e0277666. doi: 10.1371/journal.pone.0277666 (PMC9797077; doi:10.1371/journal.pone.0277666)
Supplement: Attachment — Submitted filename: PONE-D-21-31441_reviewer.pdf [file pone.0277666.s002.pdf]

PLOS ONE

Isotope systematics of subfossil, historical, and modern Nautilus macromphalus from New Caledonia

--Manuscript Draft--

**Manuscript Number:**	PONE-D-21-31441
**Article Type:**	Research Article
**Full Title:**	Isotope systematics of subfossil, historical, and modern Nautilus macromphalus from New Caledonia
**Short Title:**	Isotope systematics of Nautilus macromphalus
**Corresponding Author:**	Benjamin J. Linzmeier, Ph.D.<br>University of South Alabama<br>Mobile, Alabama UNITED STATES
**Keywords:**	Nautilus; calcium isotopes; ocean acidification; nitrogen isotopes; carbon isotopes
**Abstract:**	Cephalopod carbonate geochemistry underpins studies ranging from Phanerozoic, global-scale change to outcrop-scale paleoecological reconstructions. Interpreting these data hinges on assumed similarity to model organisms, such as Nautilus, and generalization from other molluscan biomineralization processes. Aquarium rearing and capture of wild Nautilus suggest shell carbonate precipitates quickly (35 µm/day) in oxygen isotope equilibrium with seawater. Other components of Nautilus shell chemistry are less well-studied but have potential to serve as proxies for paleobiology and paleoceanography.<br>To calibrate the geochemical response of cephalopod δ15Norg, δ13Corg, δ13Ccarb, δ18Ocarb, and δ44/40Cacarb to modern anthropogenic environmental change, we analyzed modern, historical, and subfossil Nautilus macromphalus from New Caledonia. Samples span initial human habitation, colonialization, and industrial pCO2 increase. This sampling strategy is advantageous because it avoids the shock response that can affect geochemical change in aquarium experiments. Given the range of living depths and more complex ecology of Nautilus, however, some anthropogenic signals, such as ocean acidification, may not have propagated to their living depths.<br>Our data suggest some environmental changes are more easily preserved than others given variability in cephalopod average living depth. Calculation of the percent respired carbon incorporated into the shell using δ13Corg, δ13Ccarb, and Suess-effect corrected δ13CDIC suggests an increase in the last 130 years that may have been caused by increasing carbon dioxide concentration or decreasing oxygen concentration at the depths these individuals inhabited. This pattern is consistent with increasing atmospheric CO2 and/or eutrophication offshore of New Caledonia. We find that δ44/40Ca remains stable across the last 130 years. The subfossil shell from a cenote may exhibit early δ44/40Ca diagenesis. Questions remain about the proportion of dietary vs ambient seawater calcium incorporation into the Nautilus shell. Values of δ15N do not indicate trophic level change in the last 130 years, and the subfossil shell may show diagenetic alteration of δ15N toward lower values. Future work using historical collections of Sepia and Spirula may provide additional calibration of fossil cephalopod geochemistry.
**Order of Authors:**	Benjamin J. Linzmeier, Ph.D.
	Andrew D. Jacobson, PhD
	Bradley B. Sageman, PhD
	Matthew T. Hurtgen, PhD
	Meagan E. Ankney, PhD
	Andrew Masterson, PhD
	Neil H. Landman, PhD
**Additional Information:**	

Powered by Editorial Manager® and ProduXion Manager® from Aries Systems Corporation

Question	Response
**Financial Disclosure**<br><br>Enter a financial disclosure statement that describes the sources of funding for the work included in this submission. Review the submission guidelines for detailed requirements. View published research articles from *PLOS ONE* for specific examples.<br><br>This statement is required for submission and **will appear in the published article** if the submission is accepted. Please make sure it is accurate.<br><br>**Unfunded studies**<br>Enter: *The author(s) received no specific funding for this work.*<br><br>**Funded studies**<br>Enter a statement with the following details:<br>• Initials of the authors who received each award<br>• Grant numbers awarded to each author<br>• The full name of each funder<br>• URL of each funder website<br>• Did the sponsors or funders play any role in the study design, data collection and analysis, decision to publish, or preparation of the manuscript?<br>• **NO** - Include this sentence at the end of your statement: *The funders had no role in study design, data collection and analysis, decision to publish, or preparation of the manuscript.*<br>• **YES** - Specify the role(s) played.<br><br>* typeset	Ubben Program for Climate and Carbon Science at Northwestern University to BJL, ADJ, BBS, and MTH<br><br>David and Lucile Packard Foundation (2007–31757) to ADJ
**Competing Interests**<br><br>Use the instructions below to enter a competing interest statement for this submission. On behalf of all authors, disclose any competing interests that could be perceived to bias this work—acknowledging all financial support and any other relevant financial or non-financial competing interests.	NO

Powered by Editorial Manager® and ProduXion Manager® from Aries Systems Corporation

This statement is required for submission and **will appear in the published article** if the submission is accepted. Please make sure it is accurate and that any funding sources listed in your Funding Information later in the submission form are also declared in your Financial Disclosure statement.<br><br>View published research articles from *PLOS ONE* for specific examples.<br><br>**NO authors have competing interests**<br><br>Enter: *The authors have declared that no competing interests exist.*<br><br>**Authors with competing interests**<br><br>Enter competing interest details beginning with this statement:<br><br>*I have read the journal's policy and the authors of this manuscript have the following competing interests: [insert competing interests here]*<br><br>* typeset	
**Ethics Statement**<br><br>Enter an ethics statement for this submission. This statement is required if the study involved:<br><br>• Human participants<br>• Human specimens or tissue<br>• Vertebrate animals or cephalopods<br>• Vertebrate embryos or tissues<br>• Field research<br><br>Write "N/A" if the submission does not require an ethics statement.<br><br>General guidance is provided below. Consult the submission guidelines for detailed instructions. **Make sure that all information entered here is included in the Methods section of the manuscript.**	All specimens of Nautilus macromphalus (Mollusca: Cephalopoda) used in this study were collected for morphological study of Nautilus and deposited at various museums (American Museum of Natural History, Field Museum of Natural History, Royal Ontario Museum). The methods of capture and euthanasia of Nautilus before the 2000’s specimens are unknown, but all specimens were collected before local protected status was granted in 2008. The two individuals collected in 2002 and 2004 were captured in a baited trap by Dr. Royal H. Mapes, euthanized, and kept for a morphological study of Nautilus macromphalus.

Powered by Editorial Manager® and ProduXion Manager® from Aries Systems Corporation

**Format for specific study types**<br><br>**Human Subject Research (involving human participants and/or tissue)**<br>• Give the name of the institutional review board or ethics committee that approved the study<br>• Include the approval number and/or a statement indicating approval of this research<br>• Indicate the form of consent obtained (written/oral) or the reason that consent was not obtained (e.g. the data were analyzed anonymously)<br><br>**Animal Research (involving vertebrate animals, embryos or tissues)**<br>• Provide the name of the Institutional Animal Care and Use Committee (IACUC) or other relevant ethics board that reviewed the study protocol, and indicate whether they approved this research or granted a formal waiver of ethical approval<br>• Include an approval number if one was obtained<br>• If the study involved *non-human primates*, add *additional details* about animal welfare and steps taken to ameliorate suffering<br>• If anesthesia, euthanasia, or any kind of animal sacrifice is part of the study, include briefly which substances and/or methods were applied<br><br>**Field Research**<br><br>Include the following details if this study involves the collection of plant, animal, or other materials from a natural setting:<br>• Field permit number<br>• Name of the institution or relevant body that granted permission	
**Data Availability**<br><br>Authors are required to make all data underlying the findings described fully available, without restriction, and from the time of publication. PLOS allows rare exceptions to address legal and ethical concerns. See the PLOS Data Policy and FAQ for detailed information.	Yes - all data are fully available without restriction

A Data Availability Statement describing where the data can be found is required at submission. Your answers to this question constitute the Data Availability Statement and **will be published in the article**, if accepted.<br><br>**Important:** Stating 'data available on request from the author' is not sufficient. If your data are only available upon request, select 'No' for the first question and explain your exceptional situation in the text box.<br><br>Do the authors confirm that all data underlying the findings described in their manuscript are fully available without restriction?	
**Describe where the data may be found in full sentences. If you are copying our sample text, replace any instances of XXX with the appropriate details.**<br><br>• If the data are **held or will be held in a public repository**, include URLs, accession numbers or DOIs. If this information will only be available after acceptance, indicate this by ticking the box below. For example: *All XXX files are available from the XXX database (accession number(s) XXX, XXX.).*<br>• If the data are all contained **within the manuscript and/or Supporting Information files**, enter the following: *All relevant data are within the manuscript and its Supporting Information files.*<br>• If neither of these applies but you are able to provide **details of access elsewhere**, with or without limitations, please do so. For example:<br><br>*Data cannot be shared publicly because of [XXX]. Data are available from the XXX Institutional Data Access / Ethics Committee (contact via XXX) for researchers who meet the criteria for access to confidential data.*<br><br>*The data underlying the results presented in the study are available*	All relevant data are within the manuscript and its Supporting Information files.

from (include the name of the third party and contact information or URL).<br>• This text is appropriate if the data are owned by a third party and authors do not have permission to share the data.<br><br>* typeset	
Additional data availability information:	

Manuscript

Click here to access/download;Manuscript;Isotope systematics of Nautilus Submission.docx

Preprint for submission to: PLoS One

Isotope systematics of subfossil, historical, and modern *Nautilus macromphalus* from New Caledonia

Benjamin J. Linzmeier[1], Andrew D. Jacobson[2], Bradley B. Sageman[2], Matthew T. Hurtgen[2], Meagan E. Ankney[2], Andrew Masterson[2], and Neil H. Landman[3]

[1] Department of Earth Sciences, University of South Alabama, Mobile, Alabama, USA

[2] Department of Earth and Planetary Sciences, Northwestern University, Evanston, Illinois, USA

[3] Division of Paleobiology, American Museum of Natural History, New York, New York, USA

Abstract

Cephalopod carbonate geochemistry underpins studies ranging from Phanerozoic, global-scale change to outcrop-scale paleoecological reconstructions. Interpreting these data hinges on assumed similarity to model organisms, such as *Nautilus*, and generalization from other molluscan biomineralization processes. Aquarium rearing and capture of wild *Nautilus* suggest shell carbonate precipitates quickly (35 μm/day) in oxygen isotope equilibrium with seawater. Other components of *Nautilus* shell chemistry are less well-studied but have potential to serve as proxies for paleobiology and paleoceanography.

To calibrate the geochemical response of cephalopod $\delta^{15}N_{org}$, $\delta^{13}C_{org}$, $\delta^{13}C_{carb}$, $\delta^{18}O_{carb}$, and $\delta^{44/40}Ca_{carb}$ to modern anthropogenic environmental change, we analyzed modern, historical, and subfossil *Nautilus macromphalus* from New Caledonia. Samples span initial human habitation, colonialization, and industrial pCO_2 increase. This sampling strategy is advantageous because it avoids the shock response that can affect geochemical change in aquarium experiments. Given the range of living depths and more complex ecology of *Nautilus*, however, some anthropogenic signals, such as ocean acidification, may not have propagated to their living depths.

Our data suggest some environmental changes are more easily preserved than others given variability in cephalopod average living depth. Calculation of the percent respired carbon

incorporated into the shell using $\delta^{13}C_{org}$, $\delta^{13}C_{carb}$, and Suess-effect corrected $\delta^{13}C_{DIC}$ suggests an increase in the last 130 years that may have been caused by increasing carbon dioxide concentration or decreasing oxygen concentration at the depths these individuals inhabited. This pattern is consistent with increasing atmospheric CO_2 and/or eutrophication offshore of New Caledonia. We find that $\delta^{44/40}Ca$ remains stable across the last 130 years. The subfossil shell from a cenote may exhibit early $\delta^{44/40}Ca$ diagenesis. Questions remain about the proportion of dietary vs ambient seawater calcium incorporation into the *Nautilus* shell. Values of $\delta^{15}N$ do not indicate trophic level change in the last 130 years, and the subfossil shell may show diagenetic alteration of $\delta^{15}N$ toward lower values. Future work using historical collections of *Sepia* and *Spirula* may provide additional calibration of fossil cephalopod geochemistry.

1. Introduction

The geochemistry of cephalopod mollusks has been used to quantify paleoclimate (Price et al., 2013; Urey et al., 1951) and investigate paleobiology (Ellis and Tobin, 2019; Ferguson et al., 2019; Landman et al., 2018; Linzmeier et al., 2018; Lukeneder et al., 2010; Moriya et al., 2003; Sessa et al., 2015) throughout their long fossil record (Kröger et al., 2011). Modern *Nautilus* is a model organism for the isotope geochemistry of extinct cephalopods, including the ammonoids and nautiloids, because it is the only remaining externally shelled cephalopod (Fig. 1). Extensive work has been done to quantify the isotope systematics of ~~light stable isotopes (~~$\delta^{13}C$ and $\delta^{18}O$~~)~~ in *Nautilus* shell carbonate. Aquarium rearing experiments suggest *Nautilus* shells precipitate in $\delta^{18}O$ equilibrium with seawater (Landman et al., 1994; Linzmeier et al., 2016). ~~Carbon isotope ratio data~~ ($\delta^{13}C_{shell}$) suggest both metabolic and dissolved inorganic carbon (DIC) sources (Landman et al., 1994) similar to that for other mollusks (McConnaughey and Gillikin, 2008). Analyses of $\delta^{15}N$ from intercrystalline organic matter, also known as conchiolin, show trophic

change associated with hatching and maturity (Kashiyama et al., 2010). The carbon isotope ratio of conchiolin, like organic matter in other mollusk shells, likely reflects the $\delta^{13}C$ of respired carbon (Crocker et al., 1985; Gillikin et al., 2007; McConnaughey and Gillikin, 2008).

Other isotope systems, particularly metal isotopes, have received less investigation. The calcium isotope ratio ($\delta^{44/40}Ca$) of *Nautilus* shell has only recently been measured from a handful of modern shells (Gussone and Heuser, 2016, and references therein) and may reflect changing *Nautilus* fractionation factor ($\Delta^{44/40}Ca_{shell\text{-}SW}$, (Linzmeier et al., 2020), changing $\delta^{44/40}Ca$ value of seawater (Holmden et al., 2012), or dietary change (Gussone and Heuser, 2016; Hoffmann et al., 2021).

Anthropogenic changes have impacted ocean chemistry and ecosystems through a variety of pathways. Some of these changes are particularly dramatic and likely to impact *Nautilus*, including the rise of coastal 'dead zones' (Fennel and Testa, 2019), overfishing (Barord et al., 2014), and acidification (Doney et al., 2009). For most organisms, these environmental changes are unfolding on a generational timescale, so phenotypic plasticity and ecosystem feedbacks may allow for faster adaptation, potentially mitigating the worst possible effects (Kroeker et al., 2017; Schunter et al., 2018; Telesca et al., 2021; Urban et al., 2016); however, it is difficult to assess deep-time analogs for change on timescales of 100's to 10,000's of years due to the time-averaged and punctuated nature of the fossil record (Kidwell, 2002; Kowalewski et al., 2018). Careful, high precision analyses of proxies across modern to historical samples provide an opportunity to calibrate the geochemical changes expected in fossil archives. Here, we present a suite of isotope analyses ($\delta^{13}C_{org}$, $\delta^{15}N_{org}$, $\delta^{13}C_{carb}$, $\delta^{18}O_{carb}$, and $\delta^{44/40}Ca_{carb}$) of *Nautilus macromphalus* from New Caledonia (Fig. 2). Samples range in age from modern to subfossil and span indigenous settlement (Stevenson et al., 2001), European colonization, and industrial ocean

acidification and warming (Fig. 3). Samples show analytically resolvable differences that may reflect trophic change, diagenetic alteration, or response to changing ocean chemistry through this interval. These data highlight the utility of museum collections and the subfossil record for better quantifying past environmental change to predict biological resiliency in the face of anthropogenic pressures.

2. Materials and Methods

2.1 Ethics statement

All specimens of *Nautilus macromphalus* (Mollusca: Cephalopoda) used in this study were collected for morphological study of *Nautilus* and deposited at various museums (American Museum of Natural History, Field Museum of Natural History, Royal Ontario Museum). The methods of capture and euthanasia of Nautilus before the 2000's specimens are unknown, but all specimens were collected before local protected status was granted in 2008. The two individuals collected in 2002 and 2004 were captured in a baited trap by Dr. Royal H. Mapes, euthanized, and kept for a morphological study of *Nautilus macromphalus*.

2.2 *Nautilus macromphalus* samples

Nautilus macromphalus is endemic to New Caledonia and the Loyalty Islands and distinguishable by the prominent, open umbilicus with inward sloping walls (Saunders, 2010), Fig. 1). Individual *Nautilus* likely grow to maturity over 2.5 to 6 years, although considerable uncertainty remains in their total lifespan (Landman and Cochran, 2010). The oldest samples used in this study are AMNH 11260 and AMNH 112595. These samples were recovered from a Cenote on Lifou (Fig. 2) and date to ~6800 years before present using radiocarbon (Landman et

al., 2014). Multiple fragments from near the aperture of this shell were powdered to provide adequate material for analyses. The second oldest sample is from the Field Museum of Natural History in Chicago (FM 41) and was part of the original collection purchased from Wards Scientific to start the collection after the World's Columbian Exposition in 1893. A single 2 cm^2 chip from near the aperture was removed for analysis. Three samples from the Royal Ontario Museum (ROM 60177, 48997, and 49000) were collected in 1975, 1977, and 1978 respectively. Small chips of approximately 2 cm^2 were removed from the aperture. The most recent samples, housed at the American Museum of Natural History (AMNH 105621, AMNH 123397), were collected in 2002 and 2004, respectively. One sample, AMNH 105621, was previously studied for $\delta^{18}O_{carb}$ variability by secondary ion mass spectrometry (Linzmeier et al., 2016). For this sample, powder was drilled from a chip that was embedded in epoxy.

2.3 Organic matter $\delta^{13}C$ and $\delta^{15}N$

Processing of carbonate to extract organic matter was done in the Sedimentary Geochemistry Laboratory at Northwestern University. Powdered shell was reacted with room temperature 1 N HCl overnight. Smaller amounts of recent shells with abundant organic matter were reacted with less HCl and then dried down to isolate the organic matter. The subfossil shells from the cenote required ~1-2 g of shell. Organic matter was collected from solution using ashed glass filters. Organic matter was then loaded into tin capsules and combusted at 980 ^{o} C on a Costech 4010 Elemental Analyzer, coupled to a ThermoFinnigan Delta V Plus mass spectrometer via a ConFlo IV interface in the Stable Isotope Laboratory at Northwestern University. Long term precision of $\delta^{13}C$ and $\delta^{15}N$ analyses (±0.2‰ and ±0.3‰, $1\sigma_{SD}$) was assessed by comparison with isotope standards supplied by Indiana University (Schimmelmann et al., 2009).

2.4 Carbonate $\delta^{13}C$ and $\delta^{18}O$

Analyses of carbonate powder were done in the Stable Isotope Laboratory at Northwestern University using a Thermo Gasbench II coupled with continuous flow of He carrier gas to a Thermo Delta V IRMS. Values were standardized to the Vienna Pee Dee Belemnite (VPDB) scale using NBS-18 (National Bureau of Standards) and an in-house carbonate standard, Carrera Lago Marble ($\delta^{18}O$ = -3.66, $\delta^{13}C$ = 2.31 ‰, VPDB). Analytical uncertainties ($2\sigma_{SD}$) for these analyses are ±0.2‰ for $\delta^{18}O_{carb}$ and ±0.1‰ for $\delta^{13}C_{carb}$.

2.5 Elemental analysis

Approximately 50 mg of powdered shell was placed in acid-washed HDPE test tubes and dissolved in ~10 mL of 5% HNO_3. The mixtures were agitated on a rocker table for ~24 hours, centrifuged, and then filtered through 0.45 µm syringe filters. Solutions were diluted with 5% HNO_3 to ensure elemental concentrations within calibration ranges of standards (assuming near-to-pure calcium carbonate). Measurements were done using a Thermo Scientific iCAP 6500 ICP-OES in the Aqueous Geochemistry Laboratory at Northwestern University. Repeated measurements of NIST SRM 1643f were used to both monitor instrument stability and confirm data quality. Standard measurements suggest an instrumental precision of ±5% (RSD).

2.6 Carbonate $\delta^{44/40}Ca$

Calcium isotope ratio analyses were performed in the Radiogenic Isotope Laboratory at Northwestern University using an optimized ^{43}Ca-^{42}Ca double spike method implemented on a Thermo-Fisher Triton Multi-Collector Thermal Ionization Mass Spectrometer (TIMS) (Lehn et al., 2013). Sample solutions containing 50 µg of Ca were weighed into acid-cleaned Teflon vials,

spiked, and equilibrated. Solutions were dried down overnight at 90 °C. Residues were then dissolved in 0.5 mL of 1.55N HCl, and Ca was separated from other elements by passing solutions through Teflon columns filled with Bio-Rad AG MP-50 cation exchange resin. Solutions were dried down once more and subsequently treated with two drops of 35% H_2O_2 to oxidize organic compounds and finally two drops of 16N HNO_3 to convert Ca to nitrate form. Samples were then dissolved in 0.4 µL of 8N HNO_3 and split into 4 beads. One bead was loaded onto a single degassed tantalum filament assembly, between ~0.5 mm wide parafilm dams, and subsequently dried at 3.5 amps after adding 0.5 µL of 10% H_3PO_4.

A stable, 20V ^{40}Ca ion beam was obtained after a 0.5 hr warm-up sequence. Measurement of $^{40}Ca/^{42}Ca$, $^{43}Ca/^{42}Ca$, and $^{43}Ca/^{44}Ca$ ratios was accomplished using a three-hop collector cup configuration. Sample (SMP) $^{44}Ca/^{40}Ca$ ratios are reported in delta notation relative to OSIL seawater (SW), where $\delta^{44/40}Ca_{SMP} = [(^{44}Ca/^{40}Ca)_{SMP} / (^{44}Ca/^{40}Ca)_{SW} – 1] \times 1000$. Analysis of the OSIL SW standard had reproducibility of ±0.04‰ ($2\sigma_{SD}$) during the analytical sessions including these samples. Analyses of NIST 915b yield an average of -1.14‰ ± 0.07‰ during the period of study.

All stable isotope and elemental data are available in the Supplemental data file (SI 1).

3. Results

3.1 Carbonate trace elements

Recent samples show no discernible temporal trends in either Mg/Ca or Sr/Ca ratios and display similar variation to the subfossil specimens (Fig. 4). Material from subfossil cenote specimens has higher Sr/Ca (3 mmol/mol) and lower Mg/Ca (0.5 mmol/mol) than most historical samples (Fig. 4).

3.2 Carbonate isotopes

The cenote specimens fall within the range of $\delta^{18}O$ and $\delta^{13}C$ of shell carbonate measured for modern specimens (Fig. 5). No change in $\delta^{44/40}Ca$ is apparent in these samples less than 120 years old; however, the subfossil cenote specimens are ~0.12‰ lower than modern samples (Fig. 6 A). For samples collected in the last 120 years, $\delta^{13}C_{shell}$ decreases by ~2‰ toward the present (Fig. 6 B), while $\delta^{18}O_{shell}$ increases by ~0.5‰ (Fig. 6 C).

3.3 Organic matter isotopes

Samples spanning the last 130 years show no temporal trends in either $\delta^{13}C_{org}$ or $\delta^{15}N_{org}$ (Fig. 6 D and E and Fig. 7). Subfossil cenote specimens have lower $\delta^{13}C_{org}$ and $\delta^{15}N_{org}$ by ~7‰ and ~3‰, respectively. Recent samples also consistently have lower C/N (<3) compared to subfossil specimens (>4, Fig. 7 B).

4. Discussion

4.1 Diagenesis

4.1.1 Organic matter isotopes

Although organic matter within mollusk shells has been reported for multiple fossil specimens, pristine preservation is rare (Buchardt and Weiner, 1981; Clark, 1999; Myers et al., 2018; Weiner et al., 1976). Degradation of organic matter within shells generally removes more labile amino acids and preserves recalcitrant ones, although the exact effect on $\delta^{13}C_{org}$ and $\delta^{15}N_{org}$ values is unknown. Some have noted much lower $\delta^{15}N$ in much older fossil nautiloids (*Cymatoceras sakalavus*, Albian ~113 to 100 Ma) and have suggested these reflect diagenetic alteration (Kashiyama et al., 2010). Sedimentary organic matter degradation drives $\delta^{15}N$ toward

lower values while C:N increases (Junium and Arthur, 2007). We see a similar pattern between the recent and subfossil specimens (Fig. 7 B) and therefore suggest that the $\delta^{15}N$ of the cenote specimens is altered and does not reflect a primary trophic level signature. The $\delta^{13}C_{org}$ values in these samples are also aberrant compared to the more recent specimens (Fig. 7 A). This may be due to different food available in the cenote during the last several months of life, or other unknown oceanographic circulation changes around New Caledonia.

4.1.2 Carbonate isotopes

Diagenetic alteration of light stable isotopes is of particular interest for constraining the fidelity of paleoclimatological records (Cochran et al., 2010; Immenhauser et al., 2016; Swart, 2015). The subfossil specimens from the cenote show signs that shell nacre recrystallized (Landman et al., 2014), and higher Sr/Ca in these shells is consistent with addition of either strontianite (Cochran et al., 2010) or authigenic aragonite (Fig. 4B). The lack of clear $\delta^{13}C$ or $\delta^{18}O$ outliers is notable and suggests that any recrystallization did not incorporate large volumes of additional diagenetic carbonate. Shell $\delta^{44/40}Ca$ may have been altered toward lower values, as has been observed in Cretaceous mollusks from Antarctica (Linzmeier et al., 2020), where trends toward higher Sr/Ca correlate with lower $\delta^{44/40}Ca$ values. This could point to the addition of small domains of authigenic aragonite or strontianite.

These apparent alteration effects occurred at low temperatures (~28 °C), ambient pressures, and within the last 7000 years (Landman et al., 2014). Carbonate recrystallization/precipitation was likely tied to microbial degradation of organic matter causing variable carbonate saturation state within or near shells (Eyre et al., 2018; Ries et al., 2016). Future work contrasting these diagenetic pathways with higher temperature hydrothermal

experiments (Milano et al., 2016; Pederson et al., 2019) may help provide a more detailed 'taphonomic chronometer' for use in deep time (Tomašových et al., 2017).

4.2 Isotope interpretation

4.2.1 Organic matter isotopes

Modern and historical shells suggest slight variation in prey preference or availability for the individuals measured (Fig. 7 A). In other work on *Nautilus*, $\delta^{15}N_{org}$ changes with hatching and early growth and variation of 1-2‰ within and between individuals has been observed (Kashiyama et al., 2010). Similar individual – to – individual and within individual variability may be linked to differing prey choice (Newsome et al., 2012, 2009) and is likely to be present in *Nautilus* given neurological complexity (Young, 2010). There are no strong trends through time in the recent $\delta^{13}C_{org}$ or $\delta^{15}N_{org}$ (Fig. 6 D and E and Fig. 7 A) that would suggest major trophic changes for these *Nautilus macromphalous* in the last ~120 years.

4.2.2 Calcium isotopes

In vertebrates, $\delta^{44/40}Ca$ has been proposed to be a trophic level indicator in modern and ancient terrestrial and marine ecosystems (Martin et al., 2017; Skulan and DePaolo, 1999). Ingestion of hard tissue material and subsequent use of calcium from that source in biomineralization is likely necessary to cause a $\delta^{44/40}Ca$ trophic level effect (Heuser et al., 2011). It is possible that the difference measured between the oldest *Nautilus macromphalus* and the more recent samples (Figs. 6 A, 8 F) is due to changing composition of scavenged prey, including crustaceans and fish (Saunders and Ward, 2010), perhaps due to ecosystem changes coupled to fishing pressures (Cinner et al., 2020; Guillemot et al., 2009). Shifting dietary composition causing different $\delta^{44/40}Ca$ is an unlikely explanation, however, because no strong

correlation with either $\delta^{15}N_{org}$ or $\delta^{13}C_{org}$ is observed, and moreover, recent individuals show measurable differences in both isotope systems indicative of differing diets (Fig. 8 F (Newsome et al., 2012). In addition, comparing $\delta^{44/40}Ca$ to average $\delta^{15}N$ and trophic level across other modern *Nautilus*, *Sepia*, and *Spirula* also suggests a lack of trophic response in cephalopods (Hoffmann et al., 2021).

The calcium isotope composition of mollusk material may vary with the calcium isotope composition of seawater ($\delta^{44/40}Ca_{SW}$) or changes in the fractionation between seawater and precipitated shell ($\Delta^{44/40}Ca_{SW\text{-}Shell}$) (Linzmeier et al., 2020). Bivalve mollusks derive ~80% or more of their shell calcium from seawater sources (Bevelander, 1952) and can make use of only water sources for 100% of calcium in shell precipitation (Borght and Puymbroeck, 1966). While shifts in local-scale Ca cycling dynamics are possible (Holmden et al., 2012), changes in the isotopic composition of the global ocean unlikely explain the observed variations, given the long residence time of Ca in seawater. Evidence from the geologic record, however, suggests that $\Delta^{44/40}Ca_{SW\text{-}Shell}$ is sensitive to seawater carbonate state. Culturing experiments show that the pH of extrapallial fluid from *Arctica islandica* bivalves is at least partially regulated during shell formation, but can vary with ambient pH (Liu et al., 2015). Temperature may affect $\Delta^{44/40}Ca_{SW\text{-}Shell}$ in bivalves (Hippler et al., 2013), although temperature covaries with carbonate saturation state, which can be modulated by the ratio of photosynthesis to respiration (Kelly and Hofmann, 2013; Waldbusser and Salisbury, 2014), and in general, the temperature sensitivity of $\Delta^{44/40}Ca_{SW\text{-}Shell}$ for other carbonate producers is low (Gussone et al., 2006; Gussone and Greifelt, 2019; Gussone and Heuser, 2016; Pretet et al., 2013; Sime et al., 2005).

Seawater likely constitutes the largest source of calcium for the precipitation of *Nautilus macromphalus* shell (Bevelander, 1952; Borght and Puymbroeck, 1966), but complex

precipitation and resorption of intermediate carbonate reservoirs could influence shell $\delta^{44/40}$Ca (Boucher-Rodoni and Boucher, 1993). Uroliths represent one proposed intermediary carbonate structure between seawater and shell precipitation (Schipp and Martin, 2010). These structures are amorphous calcium phosphate and have higher Sr contents than the shell wall (Lowenstam et al., 1984). The calcium fractionation factor for this material is unknown, but may be close to -1‰, similar to peloidal calcium phosphate (Gussone and Dietzel, 2016; Schmitt et al., 2003).

Fixed $\Delta^{44/40}Ca_{SW\text{-}Shell}$ in cephalopods (mostly for belemnites) is typically assumed in the reconstruction of $\delta^{44/40}Ca_{SW}$ change through time (Blättler et al., 2012; Farkaš et al., 2007a, 2007b; Hoffmann et al., 2021). Recent evidence suggests that $\Delta^{44/40}Ca_{SW\text{-}Shell}$ for other types of carbonate producers varies with seawater saturation state (Kitch et al., 2021; Linzmeier et al., 2020; Wang et al., 2020) in a way consistent with theoretical models for inorganic calcite formation (DePaolo, 2011; Mills et al., 2021). Any variation of cephalopod $\Delta^{44/40}Ca_{SW\text{-}Shell}$ may reflect additional controls, such as changes in the transport of Ca into the extrapallial fluid (EPF). One possibility is that proportions of Ca transported by selective intracellular calcium channels, non-selective intercellular pathways, or active enzymatic transport (Ca^{2+}-ATPase and carbonic anhydrase) can change. However, under most conditions, selective intracellular calcium channels are thought to dominate transportation of Ca into the EPF through high diffusive fluxes that are selective for Ca (Carré et al., 2006). Another possibility is that any calcium isotope fractionation imparted by one or more of these transport mechanisms varies with seawater saturation state. Future well-controlled aquarium rearing experiments will be necessary to determine if $\Delta^{44/40}Ca_{SW\text{-}Shell}$ changes with carbonate saturation state or another environmental condition.

For anthropogenic ocean acidification to change $\Delta^{44/40}$Ca of these *Nautilus*, higher CO_2 concentrations must propagate to their living depths, which in turn must remain relatively stable

throughout the study interval. The observed change in $\delta^{13}C$ of shell carbonate (Fig. 6B) suggests propagation of anthropogenic carbon into the habitat of the *Nautilus* but does not guarantee a change in the concentration of CO_2 necessary to cause acidification. Boron isotope measurements from shallow water (7-8 m) coral suggests a decrease of ~0.15 pH near New Caledonia since the 1890's but also indicate similar variations on decadal timescales (Wu et al., 2018). Deeper waters, like those that the *Nautilus* live in, are generally expected to acidify more slowly, although organic matter export can sometimes enhance acidification at depth depending on the interaction between deep water ventilation, organic matter export, and general ocean circulation patterns (Chen et al., 2017; Qi et al., 2017). Because the oceans are still in the early stages of acidification, it is likely that the amount of pH change at these depths has not been sufficient to cause detectable alteration in the $\delta^{44/40}Ca$ of the studied *Nautilus* specimens.

4.2.3 Respired CO_2

The proportion of respired carbon incorporated into the shells of mollusks depends on changes in ambient O_2:CO_2 in surrounding waters (McConnaughey et al., 1997; McConnaughey and Gillikin, 2008) and metabolic rate (Zhao et al., 2018). The $\delta^{13}C$ metabolic rate proxy is more advanced in fish but holds promise for cephalopods (Chung et al., 2021, 2019b, 2019a). This quantity can be calculated following a simple relationship outlined by (McConnaughey et al., 1997; McConnaughey and Gillikin, 2008):

$$C_{metabolic} = \frac{\delta^{13}C_{shell} - \varepsilon_{aragonite} - \delta^{13}C_{DIC}}{\delta^{13}C_{metabolic} - \delta^{13}C_{DIC}} \qquad (1)$$

where $\delta^{13}C_{shell}$ refers to the measured value for carbonate shell material. We assume $\delta^{13}C_{metabolic}$ is equivalent to $\delta^{13}C_{org}$ measured in the shell organic matter—given similar findings for other mollusks (Gillikin et al., 2007). The value of $\varepsilon_{aragonite}$ is assumed to be 2.7‰, which is the value

for inorganic aragonite (Romanek et al., 1992). To constrain $\delta^{13}C_{DIC}$, we use recently published estimates of $\delta^{13}C_{DIC}$ for preindustrial (1.2 ‰, VPDB) and recent (1.0 ‰, VPDB) water near New Caledonia (Eide et al., 2017b, 2017a) at the average *Nautilus macromphalus* living depth of ~400 m (Auclair et al., 2004). This depth is chosen even though shell grows across a range of depths (Linzmeier et al., 2016) due to time averaging of bulk samples like those used in this study (Linzmeier, 2019).

Calculations using these metrics show increasing amounts of respired carbon are being incorporated into the shells of *Nautilus macromphalus* during the last 130 years (Fig. 9). The observed increase (~15%) likely reflects changing metabolic rates caused by transgenerational acclimation to ocean acidification, as has been seen in *Mya arenaria* bivalves (Zhao et al., 2018). The increase is much less than the observed metabolic carbon increase (27% to 61%) in the experimentally reared bivalves over a much larger pH range (8.1 to 7.7 pH) (Zhao et al., 2018). Other experimentation with *Arctica islandica* bivalves has suggested that ontogenetic variation in the amount of respired carbon incorporated into shells is minimal (Beirne et al., 2012). Decrease of pH at the living depths of these *Nautilus macromphalus* may be facilitated by dampened deep water oxygenation related to continued organic matter export (Chen et al., 2017). The observed increase in respired carbon could also be modulated by changes in the ratio of dissolved oxygen to carbon dioxide through expanding oxygen minimum zones offshore of New Caledonia due to increased agricultural intensity and nutrient-rich runoff (Duwig et al., 1998; Gaillard and Manner, 2010), dramatic changes to vegetation (Bouchet et al., 1995), increasing [CO_2] due to anthropogenic injection into the atmosphere-ocean system (Orr et al., 2005), pressure from mining runoff (Pascal et al., 2008), or changing preferred habitat depths due to changing ecosystem composition from fishing other organisms (Fig. 3).

4.3 Future work

One of the primary challenges to clearly calibrating geochemical proxies in biogenic carbonates is the ‘shock’ of changing environmental conditions during treatment of individuals (Clark et al., 2020; Hahn et al., 2012). Although the magnitude of anthropogenic and natural environmental change can be large, both are slow relative to generation time of organisms. This means that the best calibrations for proxies preserved in biogenic carbonates may be found in historical or spatially distributed samples that have lived in conditions across several generations (Clark et al., 2020; Telesca et al., 2021). Studies in modern settings may benefit from cross-calibration with low-cost real-time environmental logging (Beddows and Mallon, 2018). Future work on these isotope systems in cephalopods and other mollusks should leverage museum collections spanning collection time and geographic range to avoid confounding factors of shock and to allow for adaptive responses. Focus on aragonitic mollusk shell is of particular merit due to the wider ranging methods for diagenetic assessment (Cochran et al., 2010; Pederson et al., 2019; Witts et al., 2018), common preservation of original mineralogy (Seuss et al., 2012), and preservation of shell organic matter in mollusks (Clark, 1999; Myers et al., 2018). Further work on internally shelled mollusks (*Sepia* and *Spirlua*) may be informative for interrogating belemnite archives given newfound microstructural complexity (Benito et al., 2016; Hoffmann et al., 2016).

5. Conclusions

Geochemical analyses of subfossil, historical, and modern *Nautilus macromphalus* show the importance of calibrating geochemical signals across generations of organisms. The low temperature diagenetic alteration of $\delta^{44/40}$Ca, elemental ratios, and δ^{15}N of organic matter also

point to the importance of robust diagenetic assessment of samples from different environments. Our data suggest that there is intergenerational increase in *Nautilus macromphalus* metabolism near New Caledonia, which is likely caused by ocean acidification. Future work using historical and modern marine mollusk shell material will be important for calibrating geochemical proxies across events that can cause both selection and/or phenotypic plasticity response.

Acknowledgements

We would like to thank Jochen Gerber (Field Museum) and Bushra Hussaini (AMNH) for assistance in accessing samples. Funding for this research was from the Ubben Program for Climate and Carbon Science at Northwestern University (Illinois, USA) and the David and Lucile Packard Foundation (2007–31757).

References

Auclair, A.-C., Lecuyer, C., Bucher, H., Sheppard, S.M.F., 2004. Carbon and oxygen isotope composition of *Nautilus macromphalus*: a record of thermocline waters off New Caledonia. Chem. Geol. 207, 91–100. https://doi.org/10.1016/j.chemgeo.2004.02.006

Barord, G.J., Dooley, F., Dunstan, A., Ilano, A., Keister, K.N., Neumeister, H., Preuss, T., Schoepfer, S., Ward, P.D., 2014. Comparative Population Assessments of *Nautilus* sp. in the Philippines, Australia, Fiji, and American Samoa Using Baited Remote Underwater Video Systems. PLoS ONE 9, e100799. https://doi.org/10.1371/journal.pone.0100799

Beddows, P.A., Mallon, E.K., 2018. Cave Pearl Data Logger: A Flexible Arduino-Based Logging Platform for Long-Term Monitoring in Harsh Environments. Sensors 18, 530. https://doi.org/10.3390/s18020530

Beirne, E.C., Wanamaker, A.D., Feindel, S.C., 2012. Experimental validation of environmental controls on the $\delta^{13}C$ of *Arctica islandica* (ocean quahog) shell carbonate. Geochim. Cosmochim. Acta 84, 395–409. https://doi.org/10.1016/j.gca.2012.01.021

Benito, M.I., Reolid, M., Viedma, C., 2016. On the microstructure, growth pattern and original porosity of belemnite rostra: insights from calcitic Jurassic belemnites. J. Iber. Geol. 42, 201–226. https://doi.org/10.5209/revBJIGE.2016.v42.n2.53232

Bevelander, G., 1952. Calcification in mollusks III. Intake and deposition of Ca^{45} and P^{32} in relation to shell formation. Biol. Bull. 102, 9–15. https://doi.org/10.2307/1538617

Blättler, C.L., Henderson, G.M., Jenkyns, H.C., 2012. Explaining the Phanerozoic Ca isotope history of seawater. Geology 40, 843–846. https://doi.org/10.1130/G33191.1

Borght, O.V.D., Puymbroeck, S.V., 1966. Calcium Metabolism in a Freshwater Mollusc : Quantitative Importance of Water and Food as Supply for Calcium During Growth. Nature 210, 791. https://doi.org/10.1038/210791a0

Boucher-Rodoni, R., Boucher, G., 1993. Respiratory quotient and calcification of *Nautilus macromphalus* (Cephalopoda: Nautiloidea). Mar. Biol. 117, 629–633.

Bouchet, P., Jaffre, T., Veillon, J.-M., 1995. Plant extinction in New Caledonia: protection of sclerophyll forests urgently needed. Biodivers. Conserv. 4, 415–428. https://doi.org/10.1007/BF00058425

Buchardt, B., Weiner, S., 1981. Diagenesis of aragonite from Upper Cretaceous ammonites: a geochemical case-study. Sedimentology 28, 423–438. https://doi.org/10.1111/j.1365-3091.1981.tb01691.x

Carré, M., Bentaleb, I., Bruguier, O., Ordinola, E., Barrett, N.T., Fontugne, M., 2006. Calcification rate influence on trace element concentrations in aragonitic bivalve shells: Evidences and mechanisms. Geochim. Cosmochim. Acta 70, 4906–4920. https://doi.org/10.1016/j.gca.2006.07.019

Chen, C.-T.A., Lui, H.-K., Hsieh, C.-H., Yanagi, T., Kosugi, N., Ishii, M., Gong, G.-C., 2017. Deep oceans may acidify faster than anticipated due to global warming. Nat. Clim. Change 7, 890. https://doi.org/10.1038/s41558-017-0003-y

Chung, M.-T., Chen, C.-Y., Shiao, J.-C., Shirai, K., Wang, C.-H., 2021. Metabolic proxy for cephalopods: Stable carbon isotope values recorded in different biogenic carbonates. Methods Ecol. Evol. 12, 1648–1657. https://doi.org/10.1111/2041-210X.13630

Chung, M.-T., Trueman, C.N., Godiksen, J.A., Grønkjær, P., 2019a. Otolith $\delta^{13}C$ values as a metabolic proxy: approaches and mechanical underpinnings. Mar. Freshw. Res. https://doi.org/10.1071/MF18317

Chung, M.-T., Trueman, C.N., Godiksen, J.A., Holmstrup, M.E., Grønkjær, P., 2019b. Field metabolic rates of teleost fishes are recorded in otolith carbonate. Commun. Biol. 2, 24. https://doi.org/10.1038/s42003-018-0266-5

Cinner, J.E., Zamborain-Mason, J., Gurney, G.G., Graham, N.A.J., MacNeil, M.A., Hoey, A.S., Mora, C., Villéger, S., Maire, E., McClanahan, T.R., Maina, J.M., Kittinger, J.N., Hicks, C.C., 2020. Meeting fisheries, ecosystem function, and biodiversity goals in a human-dominated world 6.

Clark, G.R.I., 1999. Organic matrix taphonomy in some molluscan shell microstructures. Palaeogeogr. Palaeoclimatol. Palaeoecol. 149, 305–312.

Clark, M.S., Peck, L.S., Arivalagan, J., Backeljau, T., Berland, S., Cardoso, J.C.R., Caurcel, C., Chapelle, G., Noia, M.D., Dupont, S., Gharbi, K., Hoffman, J.I., Last, K.S., Marie, A., Melzner, F., Michalek, K., Morris, J., Power, D.M., Ramesh, K., Sanders, T., Sillanpää, K., Sleight, V.A., Stewart- Sinclair, P.J., Sundell, K., Telesca, L., Vendrami, D.L.J., Ventura, A., Wilding, T.A., Yarra, T., Harper, E.M., 2020. Deciphering mollusc shell production: the roles of genetic mechanisms through to ecology, aquaculture and biomimetics. Biol. Rev. 95, 1812–1837. https://doi.org/10.1111/brv.12640

Cochran, J.K., Kallenberg, K., Landman, N.H., Harries, P.J., Weinreb, D., Turekian, K.K., Beck, A.J., Cobban, W.A., 2010. Effect of diagenesis on the Sr, O, and C isotope composition of Late Cretaceous mollusks from the Western Interior Seaway of North America. Am. J. Sci. 310, 69–88. https://doi.org/10.2475/02.2010.01

Cravatte, S., Kestenare, E., Eldin, G., Ganachaud, A., Lefèvre, J., Marin, F., Menkes, C., Aucan, J., 2015. Regional circulation around New Caledonia from two decades of observations. J. Mar. Syst. 148, 249–271. https://doi.org/10.1016/j.jmarsys.2015.03.004

Crocker, K.C., DeNiro, M.J., Ward, P.D., 1985. Stable isotopic investigations of early development in extant and fossil chambered cephalopods I. Oxygen isotopic composition of eggwater and carbon isotopic composition of siphuncle organic matter in Nautilus. Geochim. Cosmochim. Acta 49, 2527–2532. https://doi.org/10.1016/0016-7037(85)90120-6

DePaolo, D.J., 2011. Surface kinetic model for isotopic and trace element fractionation during precipitation of calcite from aqueous solutions. Geochim. Cosmochim. Acta 75, 1039–1056. https://doi.org/10.1016/j.gca.2010.11.020

Doney, S.C., Fabry, V.J., Feely, R.A., Kleypas, J.A., 2009. Ocean Acidification: The Other CO2 Problem. Annu. Rev. Mar. Sci. 1, 169–192. https://doi.org/10.1146/annurev.marine.010908.163834

Duwig, C., Becquer, T., Clothier, B.E., Vauclin, M., 1998. Nitrate leaching through oxisols of the Loyalty Islands (New Caledonia) under intensified agricultural practices. Geoderma 84, 29–43. https://doi.org/10.1016/S0016-7061(97)00119-5

Eide, M., Olsen, A., Ninnemann, U.S., Eldevik, T., 2017a. A global estimate of the full oceanic 13C Suess effect since the preindustrial. Glob. Biogeochem. Cycles 31, 492–514. https://doi.org/10.1002/2016GB005472

Eide, M., Olsen, A., Ninnemann, U.S., Johannessen, T., 2017b. A global ocean climatology of preindustrial and modern ocean δ^{13}C: Climatology of Preindustrial δ^{13}C. Glob. Biogeochem. Cycles 31, 515–534. https://doi.org/10.1002/2016GB005473

Ellis, N.M., Tobin, T.S., 2019. Evidence for seasonal variation in δ^{13}C and δ^{18}O profiles of *Baculites* and implications for growth rate. Palaeontology 62, 583–598. https://doi.org/10.1111/pala.12416

Etheridge, D.M., Steele, L.P., Langenfelds, R.L., Francey, R.J., Barnola, J.M., Morgan, V.I., 1998. Historical CO2 records from the Law Dome DE08, DE08-2, and DSS ice cores. Trends Compend. Data Glob. Change 351–364.

Eyre, B.D., Cyronak, T., Drupp, P., Carlo, E.H.D., Sachs, J.P., Andersson, A.J., 2018. Coral reefs will transition to net dissolving before end of century. Science 359, 908–911. https://doi.org/10.1126/science.aao1118

Farkaš, J., Böhm, F., Wallmann, K., Blenkinsop, J., Eisenhauer, A., van Geldern, R., Munnecke, A., Voigt, S., Veizer, J., 2007a. Calcium isotope record of Phanerozoic oceans: Implications for chemical evolution of seawater and its causative mechanisms. Geochim. Cosmochim. Acta 71, 5117–5134. https://doi.org/10.1016/j.gca.2007.09.004

Farkaš, J., Buhl, D., Blenkinsop, J., Veizer, J., 2007b. Evolution of the oceanic calcium cycle during the late Mesozoic: Evidence from $\delta^{44/40}$Ca of marine skeletal carbonates. Earth Planet. Sci. Lett. 253, 96–111. https://doi.org/10.1016/j.epsl.2006.10.015

Fennel, K., Testa, J.M., 2019. Biogeochemical Controls on Coastal Hypoxia. Annu. Rev. Mar. Sci. 11, 105–130. https://doi.org/10.1146/annurev-marine-010318-095138

Ferguson, K., Macleod, K.G., Landman, N.H., Sessa, J.A., 2019. Evaluating growth and ecology in baculitid and scaphitid ammonites using stable isotope sclerochronology. PALAIOS 34, 317–329. https://doi.org/10.2110/palo.2019.005

Gaillard, C., Manner, H.I., 2010. Yam Cultivation on the East Coast of New Caledonia: adaptation of agriculture to social and economic changes. Aust. Geogr. 41, 485–505. https://doi.org/10.1080/00049182.2010.519697

Gillikin, D.P., Lorrain, A., Meng, L., Dehairs, F., 2007. A large metabolic carbon contribution to the $\delta^{13}C$ record in marine aragonitic bivalve shells. Geochim. Cosmochim. Acta 71, 2936–2946. https://doi.org/10.1016/j.gca.2007.04.003

Guillemot, N., Léopold, M., Cuif, M., Chabanet, P., 2009. Characterization and management of informal fisheries confronted with socio-economic changes in New Caledonia (South Pacific). Fish. Res. 98, 51–61. https://doi.org/10.1016/j.fishres.2009.03.013

Gussone, N., Dietzel, M., 2016. Calcium Isotope Fractionation During Mineral Precipitation from Aqueous Solution, in: Calcium Stable Isotope Geochemistry. Springer Berlin Heidelberg, Berlin, Heidelberg, pp. 75–110. https://doi.org/10.1007/978-3-540-68953-9_3

Gussone, N., Greifelt, T., 2019. Incorporation of Ca isotopes in carapaxes of marine ostracods. Chem. Geol. 510, 130–139. https://doi.org/10.1016/j.chemgeo.2019.02.012

Gussone, N., Heuser, A., 2016. Biominerals and Biomaterial, in: Calcium Stable Isotope Geochemistry. Springer Berlin Heidelberg, Berlin, Heidelberg, pp. 111–144. https://doi.org/10.1007/978-3-540-68953-9_4

Gussone, N., Langer, G., Thoms, S., Nehrke, G., Eisenhauer, A., Riebesell, U., Wefer, G., 2006. Cellular calcium pathways and isotope fractionation in *Emiliania huxleyi*. Geology 34, 625–628. https://doi.org/10.1130/G22733.1

Hahn, S., Rodolfo-Metalpa, R., Griesshaber, E., Schmahl, W.W., Buhl, D., Hall-Spencer, J.M., Baggini, C., Fehr, K.T., Immenhauser, A., 2012. Marine bivalve shell geochemistry and ultrastructure from modern low pH environments: Environmental effect versus experimental bias. https://doi.org/10.5194/bg-9-1897-2012

Heuser, A., Tütken, T., Gussone, N., Galer, S.J.G., 2011. Calcium isotopes in fossil bones and teeth — Diagenetic versus biogenic origin. Geochim. Cosmochim. Acta 75, 3419–3433. https://doi.org/10.1016/j.gca.2011.03.032

Hippler, D., Witbaard, R., van Aken, H.M., Buhl, D., Immenhauser, A., 2013. Exploring the calcium isotope signature of *Arctica islandica* as an environmental proxy using laboratory- and field-cultured specimens. Palaeogeogr. Palaeoclimatol. Palaeoecol., Unraveling environmental histories from skeletal diaries - advances in sclerochronology 373, 75–87. https://doi.org/10.1016/j.palaeo.2011.11.015

Hoffmann, R., Richter, D.K., Neuser, R.D., Jöns, N., Linzmeier, B.J., Lemanis, R.E., Fusseis, F., Xiao, X., Immenhauser, A., 2016. Evidence for a composite organic–inorganic fabric of belemnite rostra: Implications for palaeoceanography and palaeoecology. Sediment. Geol. 341, 203–215. https://doi.org/10.1016/j.sedgeo.2016.06.001

Hoffmann, R., Riechelmann, S., Liebetrau, V., Eisenhauer, A., Immenhauser, A., 2021. Calcium isotope values of modern and fossil cephalopod shells – Trophic level or proxy for seawater geochemistry? Chem. Geol. 583, 120466. https://doi.org/10.1016/j.chemgeo.2021.120466

Holmden, C., Papanastassiou, D.A., Blanchon, P., Evans, S., 2012. $\delta^{44/40}Ca$ variability in shallow water carbonates and the impact of submarine groundwater discharge on Ca-cycling in marine environments. Geochim. Cosmochim. Acta 83, 179–194. https://doi.org/10.1016/j.gca.2011.12.031

House, M.R., 2010. Geographic Distribution of Nautilus Shells, in: Saunders, W.B., Landman, N.H. (Eds.), Nautilus: The Biology and Paleobiology of a Living Fossil, Reprint with Additions, Topics in Geobiology. Springer Netherlands, Dordrecht, pp. 53–64. https://doi.org/10.1007/978-90-481-3299-7_4

Immenhauser, A., Schöne, B.R., Hoffmann, R., Niedermayr, A., 2016. Mollusc and brachiopod skeletal hard parts: Intricate archives of their marine environment. Sedimentology 63, 1–59. https://doi.org/10.1111/sed.12231

Junium, C.K., Arthur, M.A., 2007. Nitrogen cycling during the Cretaceous, Cenomanian-Turonian Oceanic Anoxic Event II. Geochem. Geophys. Geosystems 8. https://doi.org/10.1029/2006GC001328

Kashiyama, Y., Ogawa, N.O., Chikaraishi, Y., Kashi, N., Sakai, S., Tanabe, K., Ohkouchi, N., 2010. Reconstructing the life history of modern and fossil nautiloids based on the nitrogen isotopic composition of shell organic matter and amino acids, in: Tanabe, K., Shigeta, Y., Sasaki, T., Hirano, H. (Eds.), Cephalopods - Present and Past. Tokai University Press, Tokyo, pp. 67–75.

Kelly, M.W., Hofmann, G.E., 2013. Adaptation and the physiology of ocean acidification. Funct. Ecol. 27, 980–990. https://doi.org/10.1111/j.1365-2435.2012.02061.x

Kidwell, S.M., 2002. Time-averaged molluscan death assemblages: Palimpsests of richness, snapshots of abundance. Geology 30, 803–806. https://doi.org/10.1130/0091-7613(2002)030<0803:TAMDAP>2.0.CO;2

Kitch, G.D., Jacobson, A.D., Harper, D.T., Hurtgen, M.T., Sageman, B.B., Zachos, J.C., 2021. Calcium isotope composition of *Morozovella* over the latePaleocene–early Eocene. Geology. https://doi.org/10.1130/G48619.1

Kowalewski, M., Casebolt, S., Hua, Q., Whitacre, K.E., Kaufman, D.S., Kosnik, M.A., 2018. One fossil record, multiple time resolutions: Disparate time-averaging of echinoids and mollusks on a Holocene carbonate platform. Geology 46, 51–54. https://doi.org/10.1130/G39789.1

Kroeker, K.J., Kordas, R.L., Harley, C.D.G., 2017. Embracing interactions in ocean acidification research: confronting multiple stressor scenarios and context dependence. Biol. Lett. 13, 20160802. https://doi.org/10.1098/rsbl.2016.0802

Kröger, B., Vinther, J., Fuchs, D., 2011. Cephalopod origin and evolution: A congruent picture emerging from fossils, development and molecules. BioEssays 33, 602–613. https://doi.org/10.1002/bies.201100001

Landman, N.H., Cochran, J.K., 2010. Growth and Longevity of *Nautilus*, in: Nautilus, Topics in Geobiology. Springer, Dordrecht, pp. 401–420. https://doi.org/10.1007/978-90-481-3299-7_28

Landman, N.H., Cochran, J.K., Rye, D.M., Tanabe, K., Arnold, J.M., 1994. Early life history of *Nautilus*: evidence from isotopic analyses of aquarium-reared specimens. Paleobiology 20, 40–51.

Landman, N.H., Cochran, J.K., Slovacek, M., Larson, N.L., Garb, M.P., Brezina, J., Witts, J.D., 2018. Isotope sclerochronology of ammonites (*Baculites compressus*) from methane seep and non-seep sites in the Late Cretaceous Western Interior Seaway, USA: Implications for ammonite habitat and mode of life. Am. J. Sci. 318, 603–639. https://doi.org/10.2475/06.2018.01

Landman, N.H., Mapes, R.H., Cochran, J.K., Lignier, V., Hembree, D.I., Goiran, C., Folcher, E., Brunet, P., 2014. An unusual occurrence of *Nautilus macromphalus* in a Cenote in the

Loyalty Islands (New Caledonia). PLOS ONE 9, e113372. https://doi.org/10.1371/journal.pone.0113372
Lehn, G.O., Jacobson, A.D., Holmden, C., 2013. Precise analysis of Ca isotope ratios ($\delta^{44/40}$Ca) using an optimized ^{43}Ca–^{42}Ca double-spike MC-TIMS method. Int. J. Mass Spectrom. 351, 69–75. https://doi.org/10.1016/j.ijms.2013.06.013
Linzmeier, B.J., 2019. Refining the interpretation of oxygen isotope variability in free-swimming organisms. Swiss J. Palaeontol. 138, 109–121. https://doi.org/10.1007/s13358-019-00187-3
Linzmeier, B.J., Jacobson, A.D., Sageman, B.B., Hurtgen, M.T., Ankney, M.E., Petersen, S.V., Tobin, T.S., Kitch, G.D., Wang, J., 2020. Calcium isotope evidence for environmental variability before and across the Cretaceous-Paleogene mass extinction. Geology 48, 34–38. https://doi.org/10.1130/G46431.1
Linzmeier, B.J., Kozdon, R., Peters, S.E., Valley, J.W., 2016. Oxygen isotope variability within *Nautilus* shell growth bands. PLOS ONE 11, e0153890. https://doi.org/10.1371/journal.pone.0153890
Linzmeier, B.J., Landman, N.H., Peters, S.E., Kozdon, R., Kitajima, K., Valley, J.W., 2018. Ion microprobe–measured stable isotope evidence for ammonite habitat and life mode during early ontogeny. Paleobiology 44, 684–708. https://doi.org/10.1017/pab.2018.21
Liu, Y.-W., Aciego, S.M., Wanamaker, A.D., 2015. Environmental controls on the boron and strontium isotopic composition of aragonite shell material of cultured *Arctica islandica*. Biogeosciences 12, 3351–3368. https://doi.org/10.5194/bg-12-3351-2015
Lowenstam, H.A., Traub, W., Weiner, S., 1984. *Nautilus* hard parts: a study of the mineral and organic constitutents. Paleobiology 10, 268–279.
Lukeneder, A., Harzhauser, M., Müllegger, S., Piller, W.E., 2010. Ontogeny and habitat change in Mesozoic cephalopods revealed by stable isotopes (δ^{18}O, δ^{13}C). Earth Planet. Sci. Lett. 296, 103–114. https://doi.org/10.1016/j.epsl.2010.04.053
Martin, J.E., Vincent, P., Tacail, T., Khaldoune, F., Jourani, E., Bardet, N., Balter, V., 2017. Calcium Isotopic Evidence for Vulnerable Marine Ecosystem Structure Prior to the K/Pg Extinction. Curr. Biol. 27, 1641-1644.e2. https://doi.org/10.1016/j.cub.2017.04.043
McConnaughey, T.A., Burdett, J., Whelan, J.F., Paull, C.K., 1997. Carbon isotopes in biological carbonates: Respiration and photosynthesis. Geochim. Cosmochim. Acta 61, 611–622. https://doi.org/10.1016/S0016-7037(96)00361-4
McConnaughey, T.A., Gillikin, D.P., 2008. Carbon isotopes in mollusk shell carbonates. Geo-Mar. Lett. 28, 287–299. https://doi.org/10.1007/s00367-008-0116-4
Milano, S., Prendergast, A.L., Schöne, B.R., 2016. Effects of cooking on mollusk shell structure and chemistry: Implications for archeology and paleoenvironmental reconstruction. J. Archaeol. Sci. Rep. 7, 14–26. https://doi.org/10.1016/j.jasrep.2016.03.045
Mills, J.V., DePaolo, D.J., Lammers, L.N., 2021. The influence of Ca:CO_3 stoichiometry on Ca isotope fractionation: Implications for process-based models of calcite growth. Geochim. Cosmochim. Acta 298, 87–111. https://doi.org/10.1016/j.gca.2021.01.016
Moriya, K., Nishi, H., Kawahata, H., Tanabe, K., Takayanagi, Y., 2003. Demersal habitat of Late Cretaceous ammonoids: Evidence from oxygen isotopes for the Campanian (Late Cretaceous) northwestern Pacific thermal structure. Geology 31, 167–170.
Mutvei, H., 1972. Ultrastructural relationships between the prismatic and nacreous layers in *Nautilus* (Cephalopoda). Biomineralization 4, 81–86.

Mutvei, H., 1964. On the shells of *Nautilus* and *Spirula*: with notes on shell secretion in non-cephalopod molluscs. Ark. För Zool. 16, 221–278.
Myers, C.E., Bergmann, K.D., Sun, C.-Y., Boekelheide, N., Knoll, A.H., Gilbert, P.U.P.A., 2018. Exceptional preservation of organic matrix and shell microstructure in a Late Cretaceous *Pinna* fossil revealed by photoemission electron spectromicroscopy. Geology 46, 711–714. https://doi.org/10.1130/G45271.1
Newsome, S.D., Tinker, M.T., Monson, D.H., Oftedal, O.T., Ralls, K., Staedler, M.M., Fogel, M.L., Estes, J.A., 2009. Using Stable Isotopes to Investigate Individual Diet Specialization in California Sea Otters (*Enhydra lutris nereis*). Ecology 90, 961–974.
Newsome, S.D., Yeakel, J.D., Wheatley, P.V., Tinker, M.T., 2012. Tools for quantifying isotopic niche space and dietary variation at the individual and population level. J. Mammal. 93, 329–341. https://doi.org/10.1644/11-MAMM-S-187.1
Orr, J.C., Fabry, V.J., Aumont, O., Bopp, L., Doney, S.C., Feely, R.A., Gnanadesikan, A., Gruber, N., Ishida, A., Joos, F., Key, R.M., Lindsay, K., Maier-Reimer, E., Matear, R., Monfray, P., Mouchet, A., Najjar, R.G., Plattner, G.-K., Rodgers, K.B., Sabine, C.L., Sarmiento, J.L., Schlitzer, R., Slater, R.D., Totterdell, I.J., Weirig, M.-F., Yamanaka, Y., Yool, A., 2005. Anthropogenic ocean acidification over the twenty-first century and its impact on calcifying organisms. Nature 437, 681. https://doi.org/10.1038/nature04095
Pascal, M., Forges, B.R.D., Guyader, H.L., Simberloff, D., 2008. Mining and Other Threats to the New Caledonia Biodiversity Hotspot. Conserv. Biol. 22, 498–499. https://doi.org/10.1111/j.1523-1739.2008.00889.x
Pederson, C., Mavromatis, V., Dietzel, M., Rollion-Bard, C., Nehrke, G., Jöns, N., Jochum, K.P., Immenhauser, A., 2019. Diagenesis of mollusc aragonite and the role of fluid reservoirs. Earth Planet. Sci. Lett. 514, 130–142. https://doi.org/10.1016/j.epsl.2019.02.038
Pretet, C., Samankassou, E., Felis, T., Reynaud, S., Böhm, F., Eisenhauer, A., Ferrier-Pagès, C., Gattuso, J.-P., Camoin, G., 2013. Constraining calcium isotope fractionation (δ44/40Ca) in modern and fossil scleractinian coral skeleton. Chem. Geol. 340, 49–58. https://doi.org/10.1016/j.chemgeo.2012.12.006
Price, G.D., Twitchett, R.J., Wheeley, J.R., Buono, G., 2013. Isotopic evidence for long term warmth in the Mesozoic. Sci. Rep. 3. https://doi.org/10.1038/srep01438
Qi, D., Chen, L., Chen, B., Gao, Z., Zhong, W., Feely, R.A., Anderson, L.G., Sun, H., Chen, J., Chen, M., Zhan, L., Zhang, Y., Cai, W.-J., 2017. Increase in acidifying water in the western Arctic Ocean. Nat. Clim. Change 7, 195–199. https://doi.org/10.1038/nclimate3228
Ries, J.B., Ghazaleh, M.N., Connolly, B., Westfield, I., Castillo, K.D., 2016. Impacts of seawater saturation state (ΩA=0.4–4.6) and temperature (10, 25°C) on the dissolution kinetics of whole-shell biogenic carbonates. Geochim. Cosmochim. Acta 192, 318–337. https://doi.org/10.1016/j.gca.2016.07.001
Romanek, C.S., Grossman, E.L., Morse, J.W., 1992. Carbon isotopic fractionation in synthetic aragonite and calcite: Effects of temperature and precipitation rate. Geochim. Cosmochim. Acta 56, 419–430. https://doi.org/10.1016/0016-7037(92)90142-6
Saunders, W.B., 2010. The Species of *Nautilus*, in: Nautilus, Topics in Geobiology. Springer, Dordrecht, pp. 35–52. https://doi.org/10.1007/978-90-481-3299-7_3
Saunders, W.B., Ward, P.D., 2010. Ecology, Distribution, and Population Characteristics of *Nautilus*, in: Nautilus, Topics in Geobiology. Springer, Dordrecht, pp. 137–162. https://doi.org/10.1007/978-90-481-3299-7_9

Schimmelmann, A., Albertino, A., Sauer, P.E., Qi, H., Molinie, R., Mesnard, F., 2009. Nicotine, acetanilide and urea multi-level 2H-, 13C- and 15N-abundance reference materials for continuous-flow isotope ratio mass spectrometry. Rapid Commun. Mass Spectrom. 23, 3513–3521. https://doi.org/10.1002/rcm.4277

Schipp, R., Martin, A.W., 2010. The Excretory System of Nautilus, in: Saunders, W.B., Landman, N.H. (Eds.), Nautilus: The Biology and Paleobiology of a Living Fossil, Reprint with Additions, Topics in Geobiology. Springer Netherlands, Dordrecht, pp. 281–304. https://doi.org/10.1007/978-90-481-3299-7_20

Schmitt, A.-D., Stille, P., Vennemann, T., 2003. Variations of the $^{44}Ca/^{40}Ca$ ratio in seawater during the past 24 million years: evidence from $\delta^{44}Ca$ and $\delta^{18}O$ values of Miocene phosphates. Geochim. Cosmochim. Acta 67, 2607–2614. https://doi.org/10.1016/S0016-7037(03)00100-5

Schunter, C., Welch, M.J., Nilsson, G.E., Rummer, J.L., Munday, P.L., Ravasi, T., 2018. An interplay between plasticity and parental phenotype determines impacts of ocean acidification on a reef fish. Nat. Ecol. Evol. 2, 334–342. https://doi.org/10.1038/s41559-017-0428-8

Sessa, J.A., Larina, E., Knoll, K., Garb, M., Cochran, J.K., Huber, B.T., MacLeod, K.G., Landman, N.H., 2015. Ammonite habitat revealed via isotopic composition and comparisons with co-occurring benthic and planktonic organisms. Proc. Natl. Acad. Sci. 112, 15562–15567. https://doi.org/10.1073/pnas.1507554112

Seuss, B., Titschack, J., Seifert, S., Neubauer, J., Nützel, A., 2012. Oxygen and stable carbon isotopes from a nautiloid from the middle Pennsylvanian (Late Carboniferous) impregnation Lagerstätte 'Buckhorn Asphalt Quarry' — Primary paleo-environmental signals versus diagenesis. Palaeogeogr. Palaeoclimatol. Palaeoecol. 319–320, 1–15. https://doi.org/10.1016/j.palaeo.2011.12.008

Sime, N.G., De La Rocha, C.L., Galy, A., 2005. Negligible temperature dependence of calcium isotope fractionation in 12 species of planktonic foraminifera. Earth Planet. Sci. Lett. 232, 51–66. https://doi.org/10.1016/j.epsl.2005.01.011

Skulan, J., DePaolo, D.J., 1999. Calcium isotope fractionation between soft and mineralized tissues as a monitor of calcium use in vertebrates. Proc. Natl. Acad. Sci. 96, 13709–13713. https://doi.org/10.1073/pnas.96.24.13709

Stevenson, J., Dodson, J.R., Prosser, I.P., 2001. A late Quaternary record of environmental change and human impact from New Caledonia. Palaeogeogr. Palaeoclimatol. Palaeoecol. 168, 97–123. https://doi.org/10.1016/S0031-0182(00)00251-0

Swart, P.K., 2015. The geochemistry of carbonate diagenesis: The past, present and future. Sedimentology 62, 1233–1304. https://doi.org/10.1111/sed.12205

Telesca, L., Peck, L.S., Backeljau, T., Heinig, M.F., Harper, E.M., 2021. A century of coping with environmental and ecological changes via compensatory biomineralization in mussels. Glob. Change Biol. 27, 624–639. https://doi.org/10.1111/gcb.15417

Tomašových, A., Schlögl, J., Biroň, A., Hudáčková, N., Mikuš, T., 2017. Taphonomic clock and bathymetric dependence of cephalopod preservation in bathyal, sediment-starved environments. PALAIOS 32, 135–152. https://doi.org/10.2110/palo.2016.039

Urban, M.C., Bocedi, G., Hendry, A.P., Mihoub, J.-B., Pe'er, G., Singer, A., Bridle, J.R., Crozier, L.G., Meester, L.D., Godsoe, W., Gonzalez, A., Hellmann, J.J., Holt, R.D., Huth, A., Johst, K., Krug, C.B., Leadley, P.W., Palmer, S.C.F., Pantel, J.H., Schmitz, A.,

Zollner, P.A., Travis, J.M.J., 2016. Improving the forecast for biodiversity under climate change. Science 353. https://doi.org/10.1126/science.aad8466

Urey, H.C., Lowenstam, H.A., Epstein, S., McKinney, C.R., 1951. Measurement of paleotemperatures and temperatures of the Upper Cretaceous of England, Denmark, and the Southeastern United States. GSA Bull. 62, 399–416. https://doi.org/10.1130/0016-7606(1951)62[399:MOPATO]2.0.CO;2

Waldbusser, G.G., Salisbury, J.E., 2014. Ocean acidification in the Coastal Zone from an organism's perspective: Multiple system parameters, frequency domains, and habitats. Annu. Rev. Mar. Sci. 6, 221–247. https://doi.org/10.1146/annurev-marine-121211-172238

Wang, J., Jacobson, A.D., Sageman, B.B., Hurtgen, M.T., 2020. Stable Ca and Sr isotopes support volcanically triggered biocalcification crisis during Oceanic Anoxic Event 1a. Geology. https://doi.org/10.1130/G47945.1

Ward, P.D., 1985. Periodicity of chamber formation in chambered cephalopods: Evidence from *Nautilus macromphalus* and *Nautilus pompilius*. Paleobiology 11, 438–450.

Weiner, S., Lowenstam, H.A., Hood, L., 1976. Characterization of 80-million-year-old mollusk shell proteins. Proc. Natl. Acad. Sci. 73, 2541–2545. https://doi.org/10.1073/pnas.73.8.2541

Witts, J.D., Newton, R.J., Mills, B.J.W., Wignall, P.B., Bottrell, S.H., Hall, J.L.O., Francis, J.E., Alistair Crame, J., 2018. The impact of the Cretaceous–Paleogene (K–Pg) mass extinction event on the global sulfur cycle: Evidence from Seymour Island, Antarctica. Geochim. Cosmochim. Acta 230, 17–45. https://doi.org/10.1016/j.gca.2018.02.037

Wu, H.C., Dissard, D., Douville, E., Blamart, D., Bordier, L., Tribollet, A., Le Cornec, F., Pons-Branchu, E., Dapoigny, A., Lazareth, C.E., 2018. Surface ocean pH variations since 1689 CE and recent ocean acidification in the tropical South Pacific. Nat. Commun. 9, 2543. https://doi.org/10.1038/s41467-018-04922-1

Young, J.Z., 2010. The Central Nervous System, in: Saunders, W.B., Landman, N.H. (Eds.), Nautilus: The Biology and Paleobiology of a Living Fossil, Reprint with Additions, Topics in Geobiology. Springer Netherlands, Dordrecht, pp. 215–221. https://doi.org/10.1007/978-90-481-3299-7_13

Zhao, L., Yang, F., Milano, S., Han, T., Walliser, E.O., Schöne, B.R., 2018. Transgenerational acclimation to seawater acidification in the Manila clam Ruditapes philippinarum: Preferential uptake of metabolic carbon. Sci. Total Environ. 627, 95–103. https://doi.org/10.1016/j.scitotenv.2018.01.225

Figure 1. Overview of Nautilus macromphalus shell (ROM 48997) with annotation of major shell structures, growth direction, and shell layers. Nautilus shells grow in a spiral and septa are secreted to allow for the maintenance of neutral buoyance during growth through the removal of liquid from the chambers (Ward, 1985). *Shells comprise two major structural forms of aragonite, the inner nacreous layer and outer spherulitic prismatic layer* (Mutvei, 1972, 1964).

Figure 2. Map showing the sample collection location and major currents surrounding New Caledonia. A) World map showing the location of New Caledonia in the Southwestern Pacific Ocean. B) Local map showing New Caledonia and the surrounding islands with a star indicating the location of the cenote. All cenote specimens are from Lifou, Loyalty Islands (Landman et al., 2014). *All other specimens were collected near the large island and are recent museum specimens. Reliable accounts of the live collection of Nautilus macromphalus are mostly known from areas close to New Caledonia, and the species is thought to have a geographic range restricted to that region* (House, 2010). *Local currents are overlain for reference* (Cravatte et al., 2015).

Figure 3. Timeline for sample ages, pCO_2, and human settlement of New Caledonia. Purple vertical bars represent the age of specimens used in this study with the museum repositories noted. Yellow vertical bar represents indigenous settlement as demonstrated by landscape change observed in lacustrine sediments ~3000 BCE (Stevenson et al., 2001). *French invasion occurred in the mid-1800's. The comparative record of pCO_2 change showing industrial increases is from Law Dome, East Antarctica* (Etheridge et al., 1998).

Figure 4. Crossplots of Sr/Ca and Mg/Ca ratios vs isotope ratios measured on shell carbonate. A) $\delta^{44/40}$Ca vs Mg/Ca, B) $\delta^{44/40}$Ca vs Sr/Ca, C) $\delta^{13}C_{shell}$ vs Mg/Ca, D) $\delta^{13}C_{shell}$ vs Sr/Ca, E) $\delta^{18}O_{shell}$ vs Mg/Ca, F) $\delta^{18}O_{shell}$ vs Mg/Ca, G) δ^{15}N vs Mg/Ca, H) δ^{15}N vs Sr/Ca, I) $\delta^{13}C_{org}$ vs Mg/Ca, and J) $\delta^{13}C_{org}$ vs Sr/Ca. The cenote and historical samples distinctly differ along Mg/Ca, Sr/Ca, δ^{15}N, $\delta^{13}C_{org}$, and $\delta^{44/40}$Ca axes.

Figure 5. Crossplot of carbon and oxygen isotope ratios measured on shell carbonate.

Figure 6. Stable isotope ratios through time. A) Shell $\delta^{44/40}$Ca through time. B) The δ^{13}C of shell carbonate through time. C) The δ^{18}O of shell carbonate through time. D) The δ^{15}N of shell organic matter though time. E) The δ^{13}C of shell organic matter through time.

Figure 7. Isotope ratios of organic matter and the C/N ratio of the organic matter. A) $\delta^{13}C_{org}$ vs $\delta^{15}N_{org}$, B) C/N vs $\delta^{15}N_{org}$.

Figure 8. Crossplots of isotope ratios measured in shell organic matter vs shell carbonate. A) $\delta^{13}C_{org}$ vs $\delta^{13}C_{shell}$, B) $\delta^{15}N$ vs $\delta^{13}C_{shell}$, C) $\delta^{13}C_{org}$ vs $\delta^{18}O_{shell}$, D) $\delta^{15}N$ vs $\delta^{18}O_{shell}$, E) $\delta^{13}C_{org}$ vs $\delta^{44/40}Ca$, and F) $\delta^{15}N$ vs $\delta^{44/40}Ca$.

Figure 9. Crossplot of the calculated metabolic carbon contribution vs time.

Click here to access/download;Figure;Fig1.tif

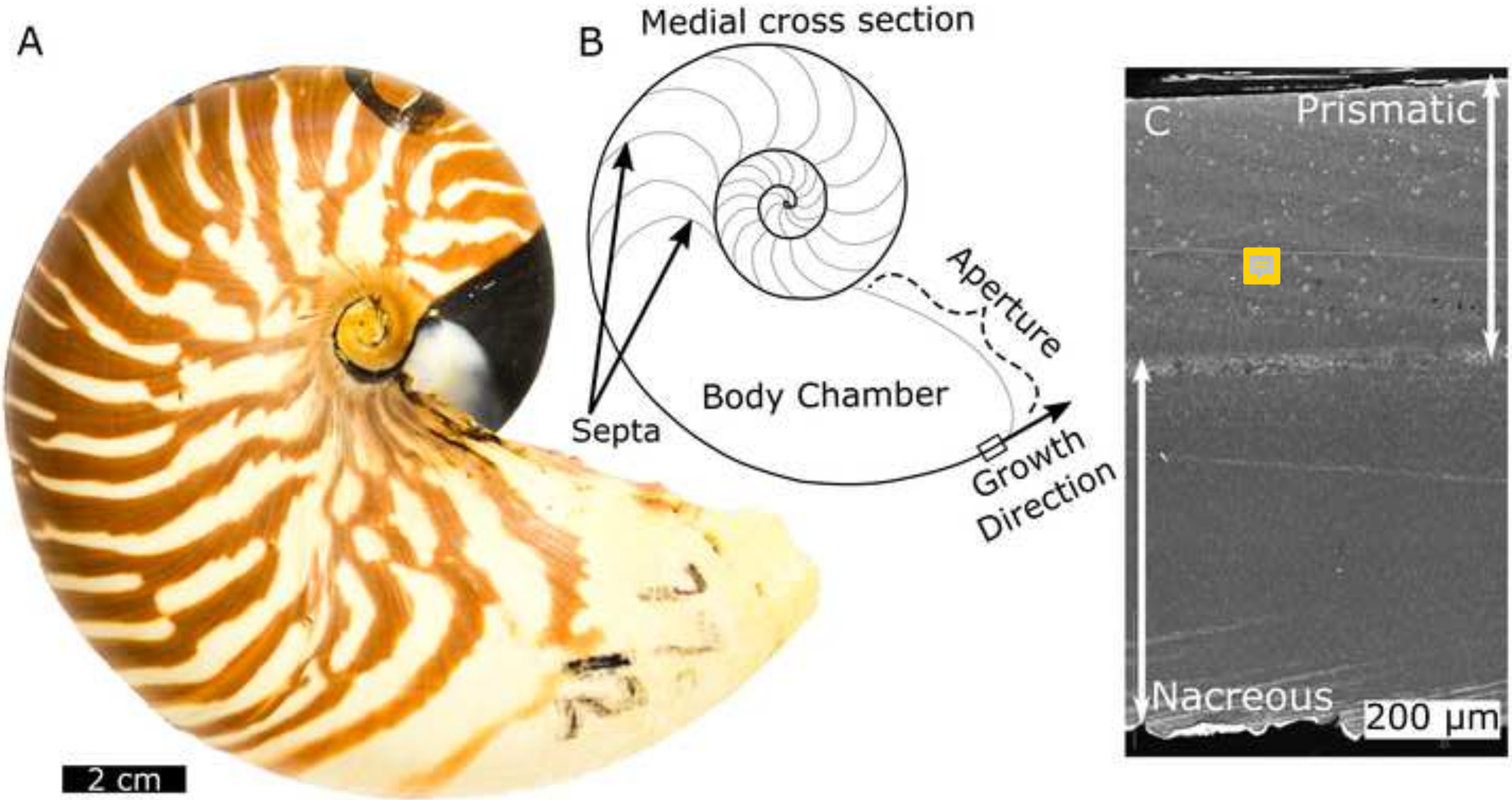

Fig2

Click here to access/download;Figure;Fig2.tif

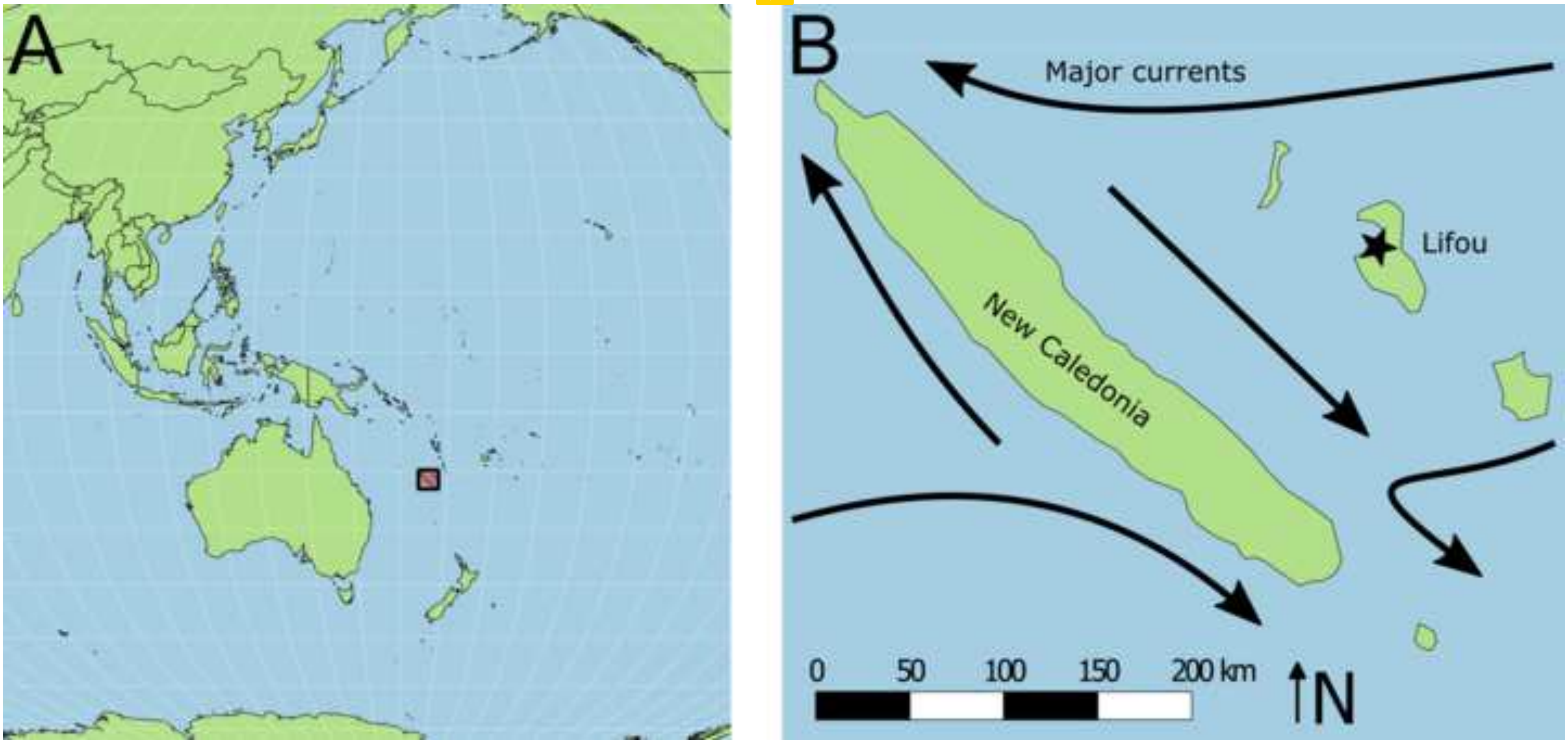

Click here to access/download;Figure;Fig3.tif

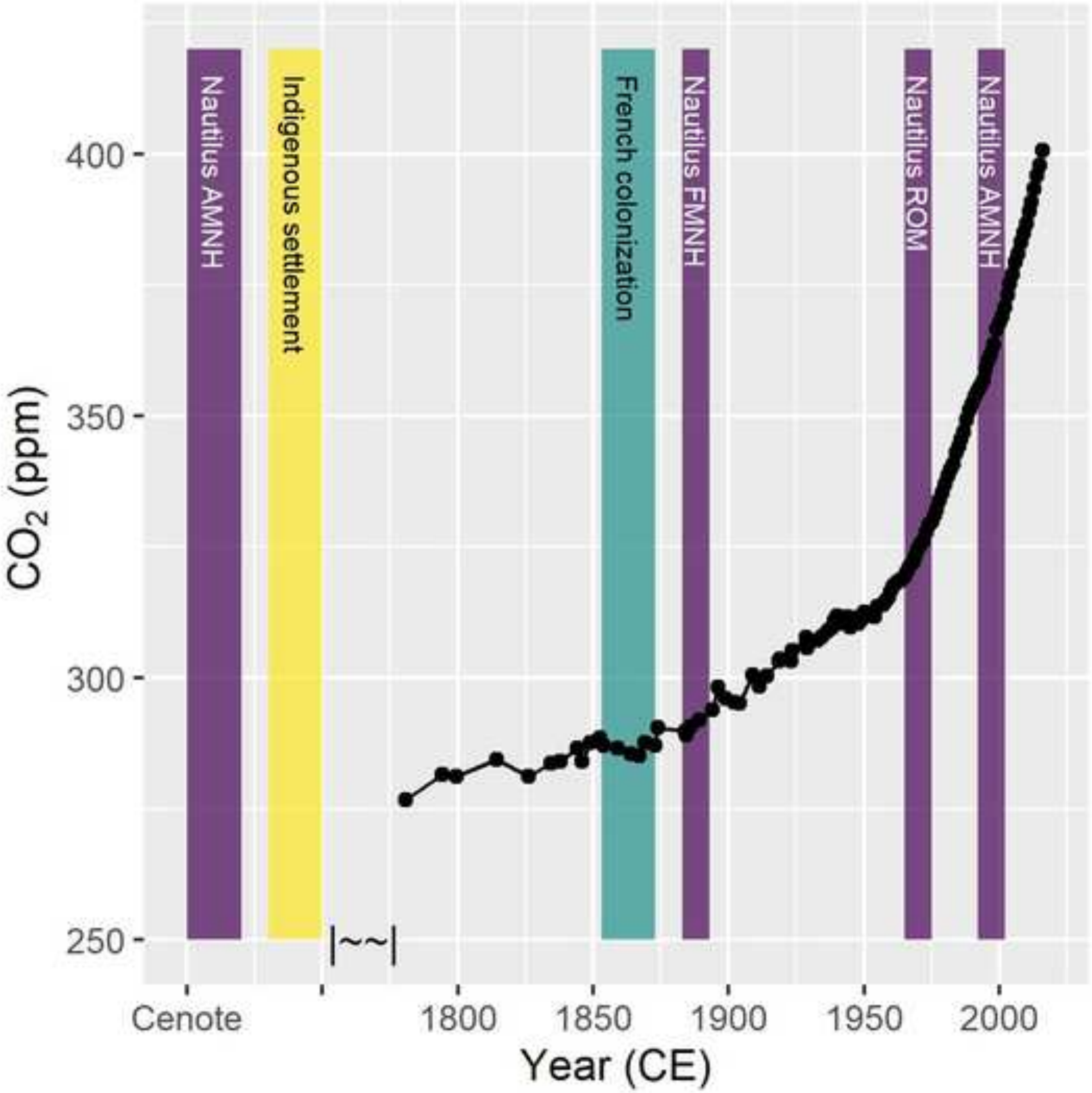

Fig4

Click here to access/download;Figure;Fig4.tif

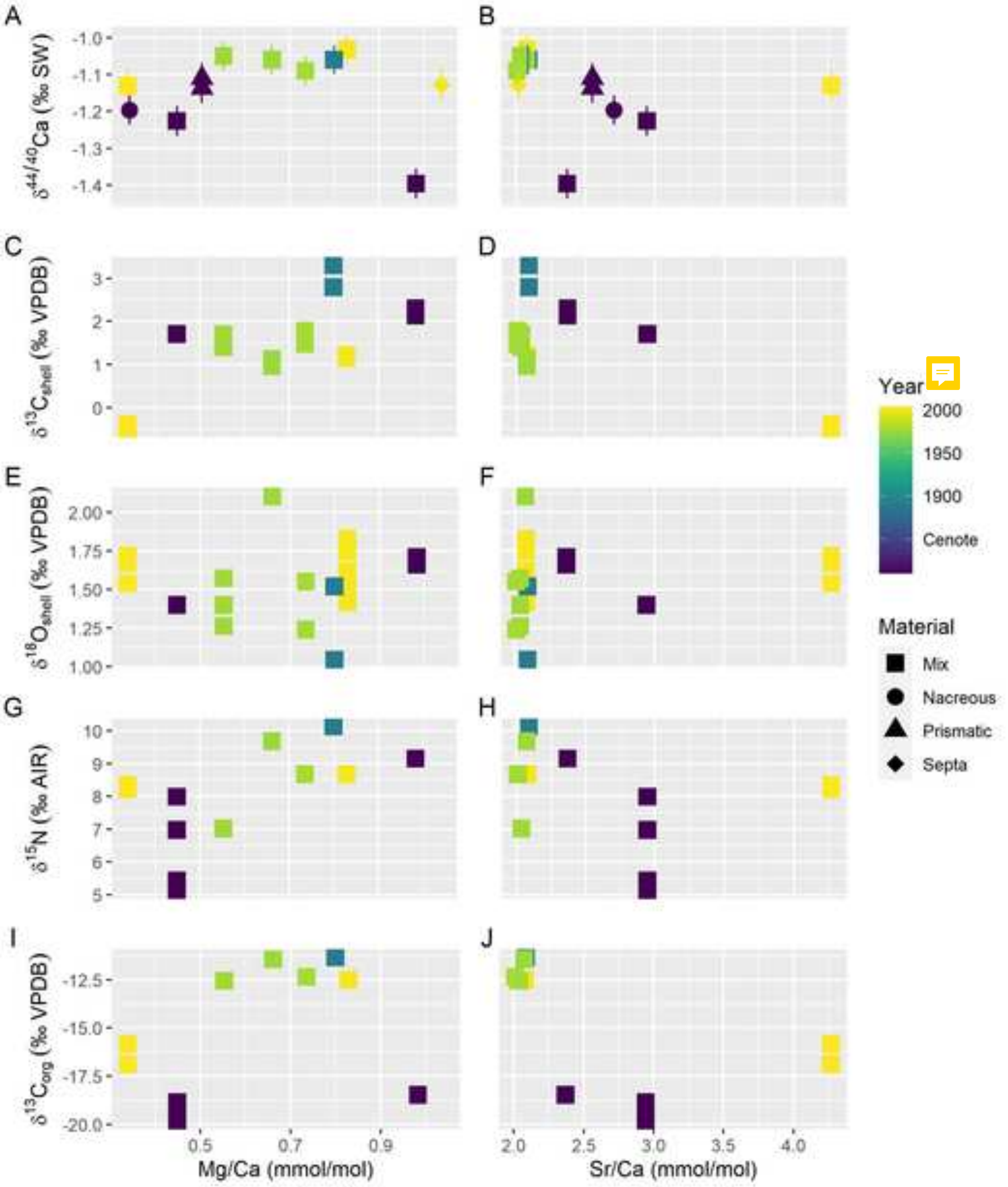

Fig5

Click here to access/download;Figure;Fig5.tif

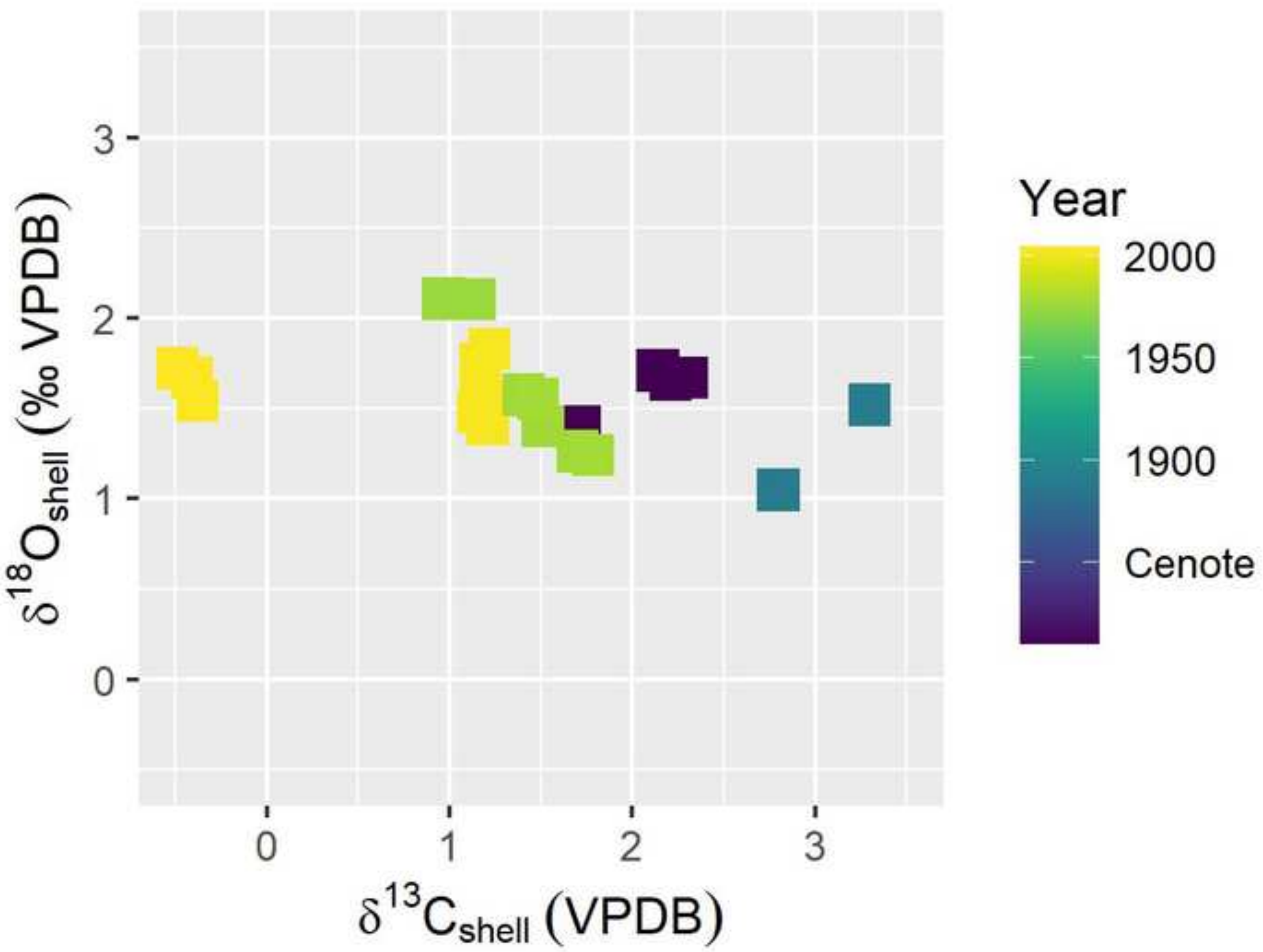

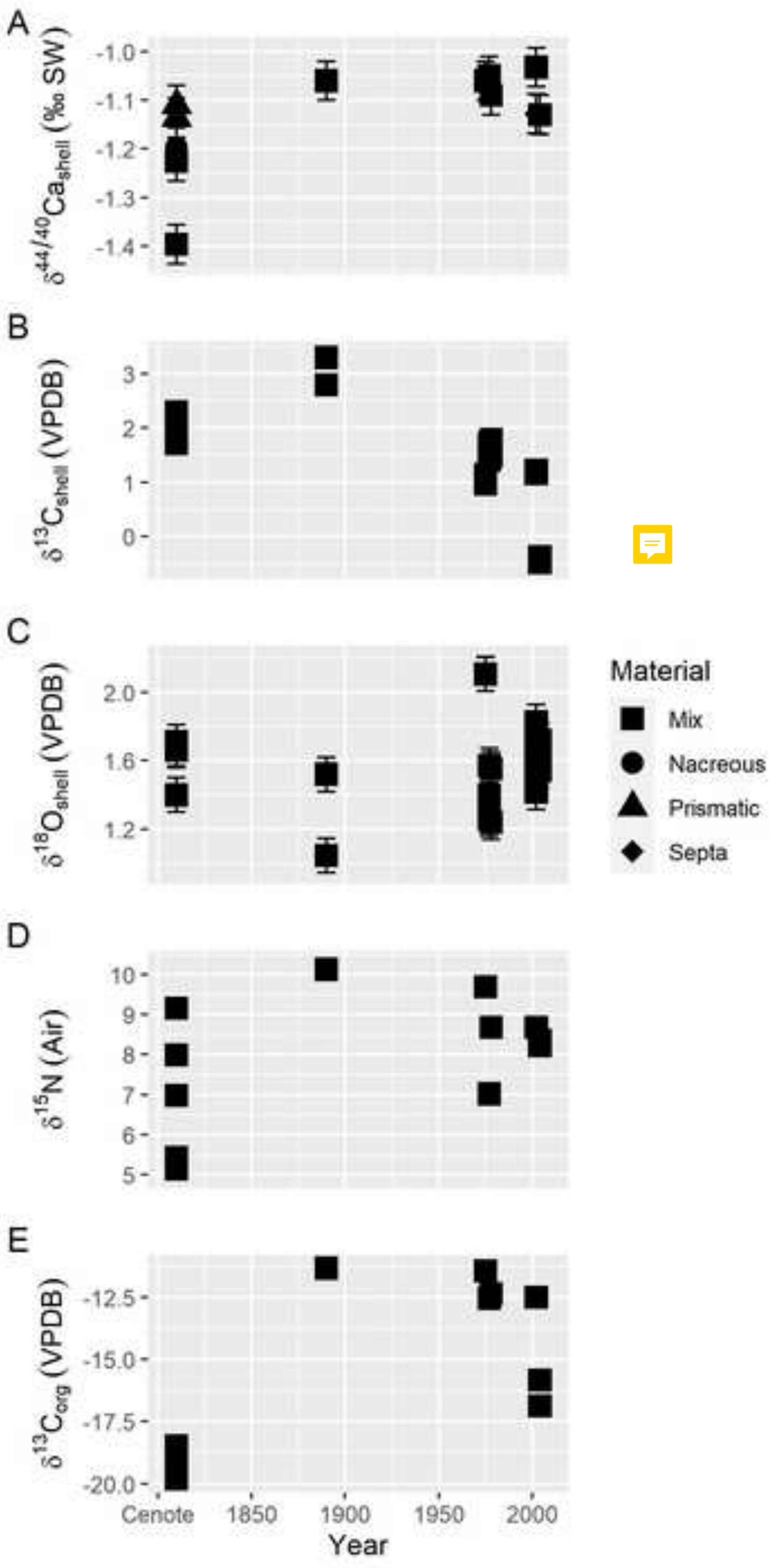
A
B
C
D
E
$\delta^{44/40}Ca_{shell}$ (‰ SW)
-1.0
-1.1
-1.2
-1.3
-1.4
$\delta^{13}C_{shell}$ (VPDB)
3
2
1
0
$\delta^{18}O_{shell}$ (VPDB)
2.0
1.6
1.2
$\delta^{15}N$ (Air)
10
9
8
7
6
5
$\delta^{13}C_{org}$ (VPDB)
-12.5
-15.0
-17.5
-20.0
Cenote
1850
1900
1950
2000
Year
Material
Mix
Nacreous
Prismatic
Septa

Fig7

Click here to access/download;Figure;Fig7.tif

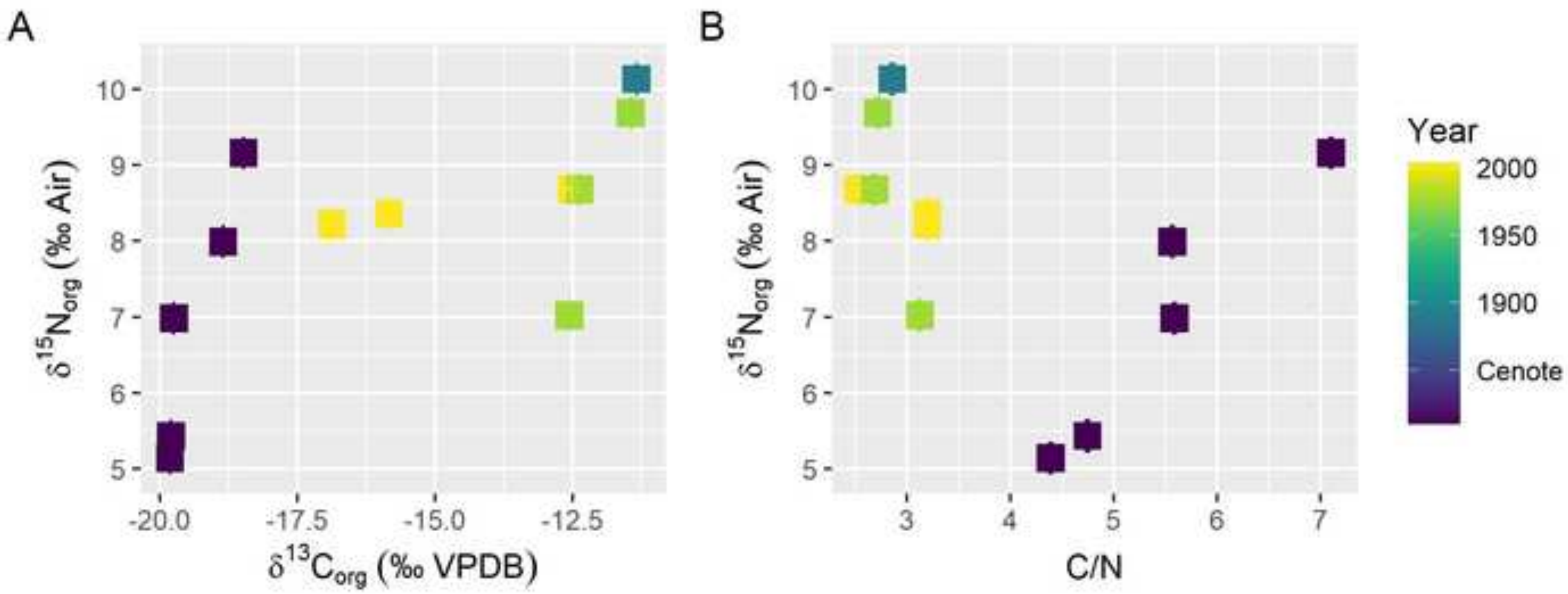

 Click here to access/download;Figure;Fig8.tif

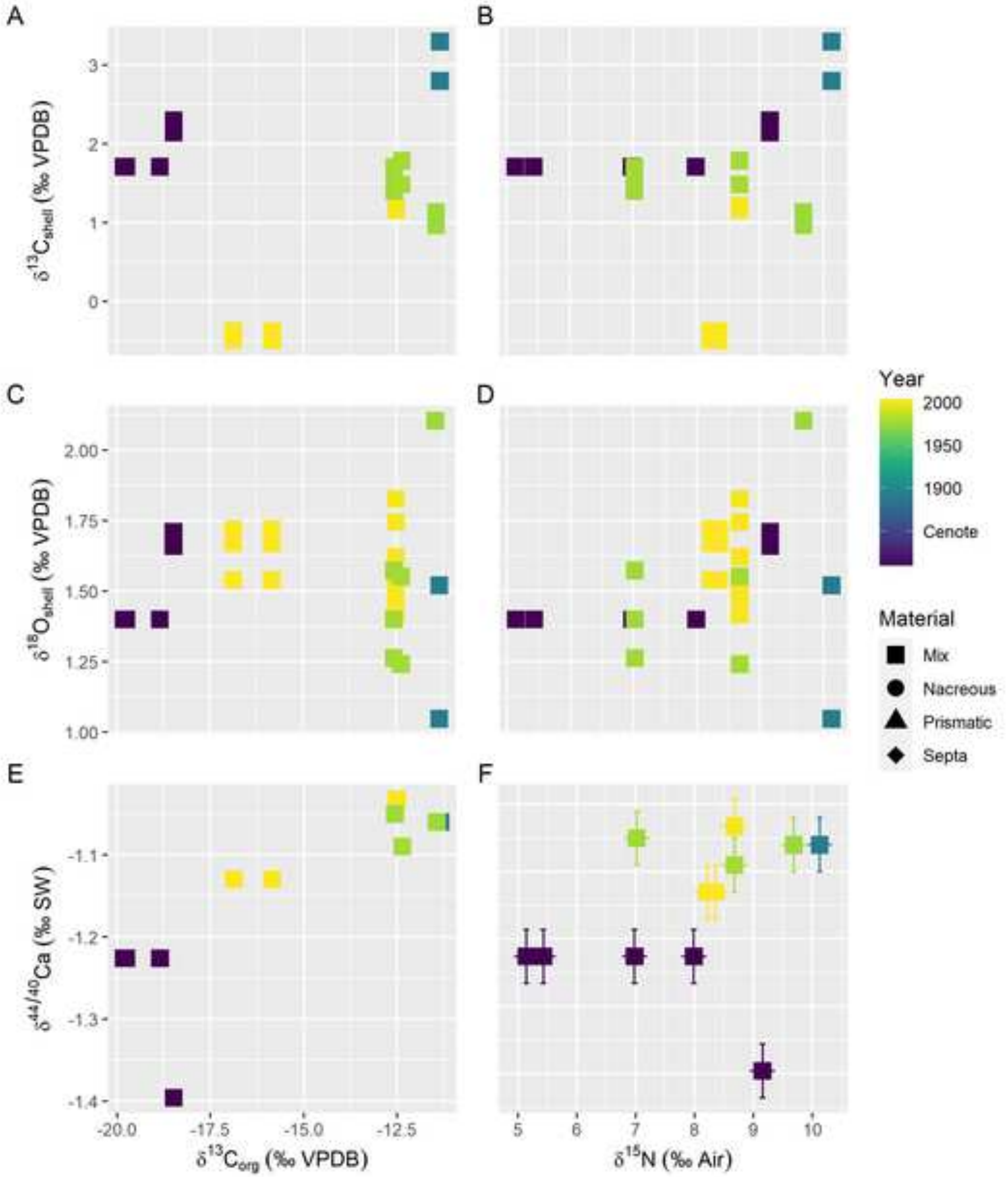

Fig9

Click here to access/download;Figure;Fig9.tif

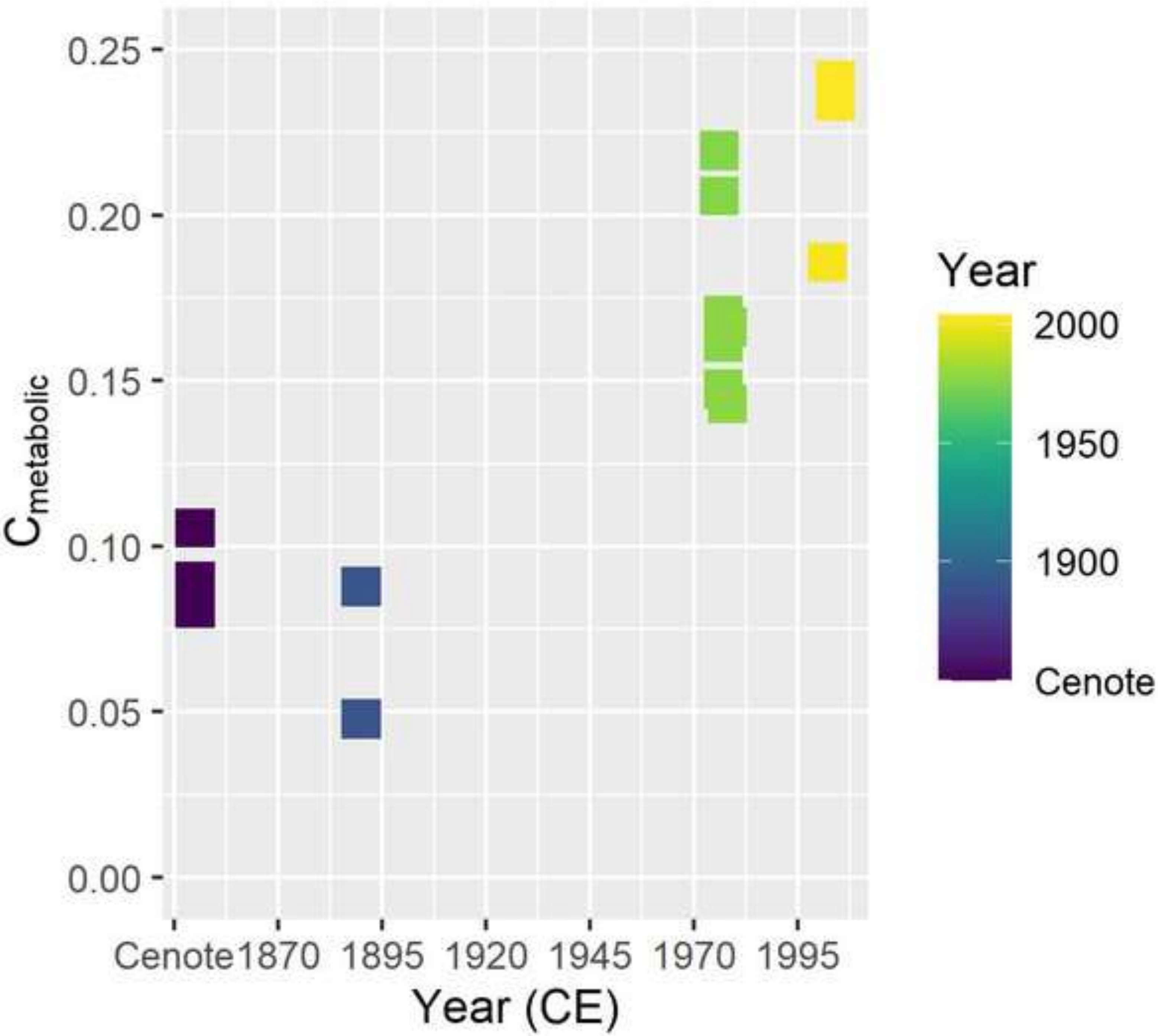

Supporting Data Table SI1

Click here to access/download
Supporting Information
SI_DataTable.xlsx
