# Supplementary material for: Isotope systematics of subfossil, historical, and modern Nautilus macromphalus from New Caledonia
Source: PLoS One. 2022 Dec 28;17(12):e0277666. doi: 10.1371/journal.pone.0277666 (PMC9797077; doi:10.1371/journal.pone.0277666)
Supplement: Attachment — Submitted filename: PONE-D-21-31441_R1.pdf [file pone.0277666.s004.pdf]

PLOS ONE

Isotope systematics of subfossil, historical, and modern Nautilus macromphalus from New Caledonia

--Manuscript Draft--

**Manuscript Number:**	PONE-D-21-31441R1
**Article Type:**	Research Article
**Full Title:**	Isotope systematics of subfossil, historical, and modern Nautilus macromphalus from New Caledonia
**Short Title:**	Isotope systematics of Nautilus macromphalus
**Corresponding Author:**	Benjamin J. Linzmeier, Ph.D.<br>University of South Alabama<br>Mobile, Alabama UNITED STATES
**Keywords:**	Nautilus; calcium isotopes; ocean acidification; nitrogen isotopes; carbon isotopes
**Abstract:**	Cephalopod carbonate geochemistry underpins studies ranging from Phanerozoic, global-scale change to outcrop-scale paleoecological reconstructions. Interpreting these data hinges on assumed similarity to model organisms, such as Nautilus , and generalization from other molluscan biomineralization processes. Aquarium rearing and capture of wild Nautilus suggest shell carbonate precipitates quickly (35 µm/day) in oxygen isotope equilibrium with seawater. Other components of Nautilus shell chemistry are less well-studied but have potential to serve as proxies for paleobiology and paleoceanography.<br>To calibrate the geochemical response of cephalopod $\delta^{15}N_{org}$, $\delta^{13}C_{org}$, $\delta^{13}C_{carb}$, $\delta^{18}O_{carb}$, and $\delta^{44/40}Ca_{carb}$ to modern anthropogenic environmental change, we analyzed modern, historical, and subfossil Nautilus macromphalus from New Caledonia. Samples span initial human habitation, colonialization, and industrial pCO_2 increase. This sampling strategy is advantageous because it avoids the shock response that can affect geochemical change in aquarium experiments. Given the range of living depths and more complex ecology of Nautilus , however, some anthropogenic signals, such as ocean acidification, may not have propagated to their living depths.<br>Our data suggest some environmental changes are more easily preserved than others given variability in cephalopod average living depth. Calculation of the percent respired carbon incorporated into the shell using $\delta^{13}C_{org}$, $\delta^{13}C_{carb}$, and Suess-effect corrected $\delta^{13}C_{DIC}$ suggests an increase in the last 130 years that may have been caused by increasing carbon dioxide concentration or decreasing oxygen concentration at the depths these individuals inhabited. This pattern is consistent with increasing atmospheric CO_2 and/or eutrophication offshore of New Caledonia. We find that $\delta^{44/40}Ca$ remains stable across the last 130 years. The subfossil shell from a cenote may exhibit early $\delta^{44/40}Ca$ diagenesis. Questions remain about the proportion of dietary vs ambient seawater calcium incorporation into the Nautilus shell. Values of $\delta^{15}N$ do not indicate trophic level change in the last 130 years, and the subfossil shell may show diagenetic alteration of $\delta^{15}N$ toward lower values. Future work using historical collections of Sepia and Spirula may provide additional calibration of fossil cephalopod geochemistry.
**Order of Authors:**	Benjamin J. Linzmeier, Ph.D.
	Andrew D. Jacobson, PhD
	Bradley B. Sageman, PhD
	Matthew T. Hurtgen, PhD
	Meagan E. Ankney, PhD
	Andrew Masterson, PhD
	Neil H. Landman, PhD
**Opposed Reviewers:**	

Powered by Editorial Manager® and ProduXion Manager® from Aries Systems Corporation

Response to Reviewers:	Dear Dr. Pucéat and reviewers,<br><br>We have addressed comments in both the cover letter and the response to reviewer document that have been uploaded. All authors wish to thank you for your time and expertise in evaluating this manuscript. We apologize for the delay in submission of revisions, but look forward to your feedback on this revised version of the manuscript.<br><br>Sincerely,<br>Benjamin Linzmeier
Additional Information:	
Question	Response
**Financial Disclosure**<br><br>Enter a financial disclosure statement that describes the sources of funding for the work included in this submission. Review the submission guidelines for detailed requirements. View published research articles from *PLOS ONE* for specific examples.<br><br>This statement is required for submission and **will appear in the published article** if the submission is accepted. Please make sure it is accurate.<br><br>**Unfunded studies**<br>Enter: *The author(s) received no specific funding for this work.*<br><br>**Funded studies**<br>Enter a statement with the following details:<br>• Initials of the authors who received each award<br>• Grant numbers awarded to each author<br>• The full name of each funder<br>• URL of each funder website<br>• Did the sponsors or funders play any role in the study design, data collection and analysis, decision to publish, or preparation of the manuscript?<br>• **NO** - Include this sentence at the end of your statement: *The funders had no role in study design, data collection and analysis, decision to publish, or preparation of the manuscript.*<br>• **YES** - Specify the role(s) played.<br><br>* typeset	Ubben Program for Climate and Carbon Science at Northwestern University to BJL, ADJ, BBS, and MTH<br><br>David and Lucile Packard Foundation (2007–31757) to ADJ<br><br>The funders had no role in study design, data collection and analysis, decision to publish, or preparation of the manuscript.
**Competing Interests**<br><br>Use the instructions below to enter a	The authors have declared that no competing interests exist.

competing interest statement for this submission. On behalf of all authors, disclose any competing interests that could be perceived to bias this work—acknowledging all financial support and any other relevant financial or non-financial competing interests.<br><br>This statement is required for submission and **will appear in the published article** if the submission is accepted. Please make sure it is accurate and that any funding sources listed in your Funding Information later in the submission form are also declared in your Financial Disclosure statement.<br><br>View published research articles from *PLOS ONE* for specific examples.<br><br>**NO authors have competing interests**<br><br>Enter: *The authors have declared that no competing interests exist.*<br><br>**Authors with competing interests**<br><br>Enter competing interest details beginning with this statement:<br><br>*I have read the journal's policy and the authors of this manuscript have the following competing interests: [insert competing interests here]*<br><br>* typeset	
**Ethics Statement**<br><br>Enter an ethics statement for this submission. This statement is required if the study involved:<br><br>• Human participants<br>• Human specimens or tissue<br>• Vertebrate animals or cephalopods<br>• Vertebrate embryos or tissues<br>• Field research	All specimens of Nautilus macromphalus (Mollusca: Cephalopoda) used in this study were collected for morphological study of Nautilus and deposited at various museums (American Museum of Natural History, Field Museum of Natural History, Royal Ontario Museum). The methods of capture and euthanasia of Nautilus before the 2000's specimens are unknown, but all specimens were collected before local protected status was granted in 2008. The two individuals collected in 2002 and 2004 were captured in a baited trap by Dr. Royal H. Mapes, euthanized, and kept for a morphological study of Nautilus macromphalus.

Powered by Editorial Manager® and ProduXion Manager® from Aries Systems Corporation

Write "N/A" if the submission does not require an ethics statement.

General guidance is provided below. Consult the submission guidelines for detailed instructions. **Make sure that all information entered here is included in the Methods section of the manuscript.**

Format for specific study types

Human Subject Research (involving human participants and/or tissue)

- Give the name of the institutional review board or ethics committee that approved the study
- Include the approval number and/or a statement indicating approval of this research
- Indicate the form of consent obtained (written/oral) or the reason that consent was not obtained (e.g. the data were analyzed anonymously)

Animal Research (involving vertebrate animals, embryos or tissues)

- Provide the name of the Institutional Animal Care and Use Committee (IACUC) or other relevant ethics board that reviewed the study protocol, and indicate whether they approved this research or granted a formal waiver of ethical approval
- Include an approval number if one was obtained
- If the study involved *non-human primates*, add *additional details* about animal welfare and steps taken to ameliorate suffering
- If anesthesia, euthanasia, or any kind of animal sacrifice is part of the study, include briefly which substances and/or methods were applied

Field Research

Include the following details if this study involves the collection of plant, animal, or other materials from a natural setting:

- Field permit number
- Name of the institution or relevant body that granted permission

Powered by Editorial Manager® and ProduXion Manager® from Aries Systems Corporation

**Data Availability**<br><br>Authors are required to make all data underlying the findings described fully available, without restriction, and from the time of publication. PLOS allows rare exceptions to address legal and ethical concerns. See the PLOS Data Policy and FAQ for detailed information.<br><br>A Data Availability Statement describing where the data can be found is required at submission. Your answers to this question constitute the Data Availability Statement and **will be published in the article**, if accepted.<br><br>**Important:** Stating 'data available on request from the author' is not sufficient. If your data are only available upon request, select 'No' for the first question and explain your exceptional situation in the text box.<br><br>Do the authors confirm that all data underlying the findings described in their manuscript are fully available without restriction?	Yes - all data are fully available without restriction
**Describe where the data may be found in full sentences. If you are copying our sample text, replace any instances of XXX with the appropriate details.**<br><br>• If the data are **held or will be held in a public repository**, include URLs, accession numbers or DOIs. If this information will only be available after acceptance, indicate this by ticking the box below. For example: *All XXX files are available from the XXX database (accession number(s) XXX, XXX.*).<br>• If the data are all contained **within the manuscript and/or Supporting Information files**, enter the following: *All relevant data are within the manuscript and its Supporting Information files.*<br>• If neither of these applies but you are able to provide **details of access elsewhere**, with or without limitations, please do so. For example:	All relevant data are within the manuscript and its Supporting Information files.

Data cannot be shared publicly because of [XXX]. Data are available from the XXX Institutional Data Access / Ethics Committee (contact via XXX) for researchers who meet the criteria for access to confidential data.<br><br>*The data underlying the results presented in the study are available from (include the name of the third party and contact information or URL).*<br>• This text is appropriate if the data are owned by a third party and authors do not have permission to share the data.<br><br>* typeset	
Additional data availability information:	

Dear Dr. Pucéat,

Thank you for handling our manuscript. The authors also thank the reviewers for their thorough comments and review of our manuscript. We have responded to comments in the “Response to reviewers” document and in a revised version of the manuscript.

We have revised the figures to group samples into Cenote, 1890’s, 1970’s, and 2000’s and reduced the number of colors as suggested by the reviewers. This retains the major groups but loses some information given the slight difference in ages within each decade. This information is retained in the supplemental tables and as the reviewers stated, was not visible in the previous versions of the figures.

We have expanded and clarified our hypotheses in the introduction and the discussion. Hypothesis testing in this manuscript is focused mostly on the timing of change in proxy values vs a quantitative prediction based on field datasets. This is because some of these (e.g. pH since 1850 at *Nautilus* living depths) are unavailable at New Caledonia. The presence or absence of shifts in measured values and the timing of the shifts allows deduction about the proxy response to environmental change.

There were several points to address from the editorial decision letter:

We would like to add the following sentence to the financial disclosure statement. The funders had no role in study design, data collection and analysis, decision to publish, or preparation of the manuscript.

We would like to change the statement of competing interests to include the following: The authors have declared that no competing interests exist.

The map in Figure 2 contains shapefiles from Natural Earth. The data are freely available for use and their terms of use can be found here (https://www.naturalearthdata.com/about/terms-of-use/).

References and formatting have been changed to comply with the requirements of PLoS ONE.

Thank you for your consideration of this revised manuscript and we look forward to hearing your decision.

Sincerely,

Benjamin J. Linzmeier

Manuscript

Click here to access/download;Manuscript;Isotope systematics of Nautilus Revised Manuscript.docx

Isotope systematics of subfossil, historical, and modern *Nautilus macromphalus* from New Caledonia

Benjamin J. Linzmeier[1*], Andrew D. Jacobson[2], Bradley B. Sageman[2], Matthew T. Hurtgen[2], Meagan E. Ankney[3], Andrew Masterson[2], and Neil H. Landman[4]

[1] Department of Earth Sciences, University of South Alabama, Mobile, Alabama, United States of America

[2] Department of Earth and Planetary Sciences, Northwestern University, Evanston, Illinois, United States of America

[3] Wisconsin State Laboratory of Hygiene, University of Wisconsin – Madison, Madison, Wisconsin, United States of America

[4] Division of Paleontology, American Museum of Natural History, New York, New York, United States of America

* Corresponding author

Email: blinzmeier@southalabama.edu

Abstract

Cephalopod carbonate geochemistry underpins studies ranging from Phanerozoic, global-scale change to outcrop-scale paleoecological reconstructions. Interpreting these data hinges on assumed similarity to model organisms, such as *Nautilus*, and generalization from other molluscan biomineralization processes. Aquarium rearing and capture of wild *Nautilus* suggest shell carbonate precipitates quickly (35 µm/day) in oxygen isotope equilibrium with seawater. Other components of *Nautilus* shell chemistry are less well-studied but have potential to serve as proxies for paleobiology and paleoceanography.

To calibrate the geochemical response of cephalopod $\delta^{15}N_{org}$, $\delta^{13}C_{org}$, $\delta^{13}C_{carb}$, $\delta^{18}O_{carb}$, and $\delta^{44/40}Ca_{carb}$ to modern anthropogenic environmental change, we analyzed modern, historical, and subfossil *Nautilus macromphalus* from New Caledonia. Samples span initial human habitation, colonialization, and industrial pCO_2 increase. This sampling strategy is advantageous because it

avoids the shock response that can affect geochemical change in aquarium experiments. Given the range of living depths and more complex ecology of *Nautilus*, however, some anthropogenic signals, such as ocean acidification, may not have propagated to their living depths.

Our data suggest some environmental changes are more easily preserved than others given variability in cephalopod average living depth. Calculation of the percent respired carbon incorporated into the shell using $\delta^{13}C_{org}$, $\delta^{13}C_{carb}$, and Suess-effect corrected $\delta^{13}C_{DIC}$ suggests an increase in the last 130 years that may have been caused by increasing carbon dioxide concentration or decreasing oxygen concentration at the depths these individuals inhabited. This pattern is consistent with increasing atmospheric CO_2 and/or eutrophication offshore of New Caledonia. We find that $\delta^{44/40}Ca$ remains stable across the last 130 years. The subfossil shell from a cenote may exhibit early $\delta^{44/40}Ca$ diagenesis. Questions remain about the proportion of dietary vs ambient seawater calcium incorporation into the *Nautilus* shell. Values of $\delta^{15}N$ do not indicate trophic level change in the last 130 years, and the subfossil shell may show diagenetic alteration of $\delta^{15}N$ toward lower values. Future work using historical collections of *Sepia* and *Spirula* may provide additional calibration of fossil cephalopod geochemistry.

Introduction

The geochemistry of cephalopod mollusks has been used to quantify paleoclimate [1,2] and investigate paleobiology [3–9] throughout their long fossil record [10]. Modern *Nautilus* is a model organism for the isotope geochemistry of extinct cephalopods, including the ammonoids and nautiloids, because it is the only remaining externally shelled cephalopod (Fig 1). *Nautilus* are forereef scavengers that cross hundreds of meters of water depth within individual days while continuously growing shell . Extensive work has been done to quantify the systematics of light

stable isotopes ($\delta^{13}C$ and $\delta^{18}O$) in *Nautilus* shell carbonate. Aquarium rearing experiments suggest *Nautilus* shells precipitate in $\delta^{18}O$ equilibrium with seawater [11,12]. Carbon isotope values ($\delta^{13}C_{shell}$) suggest both metabolic and dissolved inorganic carbon (DIC) sources [11] similar to that for other mollusks [13]. Analyses of $\delta^{15}N$ from shell organic matter, also known as conchiolin, show trophic change associated with hatching and maturity [14]. The carbon isotope value of conchiolin, like organic matter in other mollusk shells, likely reflects the $\delta^{13}C$ of respired carbon [13,15,16].

Fig 1. Overview of *Nautilus m.* shell (ROM 48997) with annotation of major shell structures, growth direction, and shell layers. *Nautilus* shells grow in a spiral and septa are secreted to allow for the maintenance of neutral buoyance during growth through the removal of liquid from the chambers [17]. Shells comprise two major structural forms of aragonite, the inner nacreous layer and outer spherulitic prismatic layer [18,19].

Other isotope systems, particularly metal isotopes, have received less investigation. The calcium isotope value ($\delta^{44/40}Ca$) of *Nautilus* shell has only recently been measured from a handful of modern shells (Gussone and Heuser, 2016, and references therein) and may reflect changing *Nautilus* fractionation factor ($\Delta^{44/40}Ca_{shell\text{-}SW}$, [21], changing $\delta^{44/40}Ca$ value of seawater [22], or dietary change [20,23].

Proxies respond to anthropogenic forcings of environmental change. Some of these shifts are particularly dramatic and likely to impact *Nautilus*, including the rise of coastal ‘dead zones’ [24], overfishing [25], and acidification [26]. For most organisms, these environmental changes are unfolding on a generational timescale, so phenotypic plasticity and ecosystem feedbacks may allow for faster adaptation, potentially mitigating the worst possible effects [27–30]; however, it

is difficult to assess deep-time analogs for change on timescales of 100's to 10,000's of years due to the time-averaged and punctuated nature of the fossil record [31,32]. Careful, high precision analyses of proxies across modern to historical samples provide an opportunity to calibrate the geochemical changes expected in fossil archives and provide insight into variations expected to be preserved within fossil assemblages during abrupt climate events.

Here, we test for hypothesized anthropogenic forcings (e.g. temperature change, ocean acidification, trophic change) and diagenetic vulnerability present in a suite of analyses (Sr/Ca, Mg/Ca, $\delta^{13}C_{org}$, $\delta^{15}N_{org}$, $\delta^{13}C_{carb}$, $\delta^{18}O_{carb}$, and $\delta^{44/40}Ca_{carb}$) of *Nautilus macromphalus* from New Caledonia (Fig 2). Samples range in age from modern to subfossil and span indigenous settlement [33], European colonization, and industrial ocean acidification and warming (Fig 3). We hypothesize abrupt changes in ecologically sensitive proxies (e.g. $\delta^{18}O_{carb}$, $\delta^{15}N_{org}$) are stepwise due to ecological restructuring, while gradual changes are forced due to continuous industrial environmental effects. These data highlight the utility of museum collections and the subfossil record for better quantifying past environmental change to predict biological resiliency in the face of anthropogenic pressures.

Fig 2. Map showing the sample collection location and major currents surrounding New Caledonia. Data are from www.naturalearthdata.com. A) World map showing the location of New Caledonia in the Southwestern Pacific Ocean. B) Local map showing New Caledonia and the surrounding islands with a star indicating the location of the cenote. Water shallower than 1000 m is in light blue and approximates the habitat of Nautilus m. around New Caledonia. All cenote specimens are from the star location of Lifou, Loyalty Islands [34]. All other specimens were collected near the large island and are recent museum specimens. Reliable accounts of the live collection of Nautilus m. are mostly known from areas close to New Caledonia, and the

species is thought to have a geographic range restricted to that region [35]. Local currents are overlain for reference [36].

Fig 3. Timeline for sample ages, pCO_2, and human settlement of New Caledonia. Purple vertical bars represent the age of specimens used in this study with the museum repositories noted. Yellow vertical bar represents indigenous settlement as demonstrated by landscape change observed in lacustrine sediments ~3000 BCE [33]. French invasion occurred in the mid-1800's. The comparative record of pCO_2 change showing industrial increases is from Law Dome, East Antarctica [37].

Materials and Methods

Ethics statement

All specimens of *Nautilus macromphalus* (Mollusca: Cephalopoda) used in this study were collected for morphological study of *Nautilus* and deposited at various museums (American Museum of Natural History, Field Museum of Natural History, Royal Ontario Museum). The methods of capture and sacrifice of Nautilus before the 2000's specimens are unknown, but all specimens were collected before local protected status was granted in 2008. The two individuals collected in 2002 and 2004 were captured in a baited trap by Dr. Royal H. Mapes, sacrificed, and kept for a morphological study.

Nautilus macromphalus samples

Nautilus m. is endemic to New Caledonia and the Loyalty Islands and can be distinguished by the prominent, open umbilicus with inward sloping walls ([38], Fig 1). Individual *Nautilus* likely grow to maturity over 2.5 to 6 years, although considerable uncertainty

remains in their total lifespan [39]. The oldest samples used in this study are AMNH 112600 and AMNH 112595. These samples were recovered from a cenote on Lifou (Fig 2) and date to 6455 ±30 (AMNH 112600) and 6895 ±30 (AMNH 112595) years before present using radiocarbon [34]. Multiple fragments from near the aperture of this shell were powdered to provide adequate material for analyses. The second oldest sample is from the Field Museum of Natural History in Chicago (FM 41) and was part of the original collection purchased from Wards Scientific to start the collection after the World's Columbian Exposition in 1893. A single 2 cm^2 chip from near the aperture was removed for analysis. Three samples from the Royal Ontario Museum (ROM 60177, 48997, and 49000) were collected in 1975, 1977, and 1978 respectively. Small chips of approximately 2 cm^2 were removed from the aperture. The most recent samples, housed at the American Museum of Natural History (AMNH 105621, AMNH 123397), were collected in 2002 and 2004, respectively. One sample, AMNH 105621, was previously studied for $\delta^{18}O_{carb}$ variability by secondary ion mass spectrometry [12]. For this sample, powder was drilled from a chip that was embedded in epoxy.

Organic matter $\delta^{13}C$ and $\delta^{15}N$

Processing of carbonate to extract organic matter was done in the Sedimentary Geochemistry Laboratory at Northwestern University. Powdered shell was reacted with room temperature 1 N HCl overnight. Smaller amounts of recent shells with abundant organic matter were reacted with smaller volumes of HCl necessary for complete dissolution and then dried down to isolate the organic matter. The subfossil shells from the cenote required ~1-2 g of shell for the analyses presented here. Organic matter was collected from solution using ashed glass filters. Either organic matter or organic matter plus filters were loaded into tin capsules and combusted at 980 ^{o} C on a Costech 4010 Elemental Analyzer, coupled to a ThermoFinnigan

Delta V Plus mass spectrometer via a ConFlo IV interface in the Stable Isotope Laboratory at Northwestern University. Long term precision of $\delta^{13}C$ and $\delta^{15}N$ analyses (±0.4‰ and ±0.6‰, $2\sigma_{SD}$) was assessed by comparison with isotope standards IU acetanilide ($\delta^{13}C$ = -29.52±0.02‰, $\delta^{15}N$ = +1.18‰) and IU urea ($\delta^{13}C$ = -8.02±0.05‰, $\delta^{15}N$ = +20.17±0.06‰) supplied by Indiana University [40]. This method of measurement does not distinguish between carbonate-bound organic matter (intracrystalline) and non-carbonate bound organic matter (intercrystalline) but produces average $\delta^{13}C$ and $\delta^{15}N$ values for all organic matter isolated from the *Nautilus* shell.

Carbonate $\delta^{13}C$ and $\delta^{18}O$

Analyses of carbonate powder were conducted in the Stable Isotope Laboratory at Northwestern University using a Thermo Gasbench II coupled with continuous flow of He carrier gas to a Thermo Delta V IRMS. Values were standardized to the Vienna Pee Dee Belemnite (VPDB) scale using NBS-18 (National Bureau of Standards, $\delta^{18}O$ = -23.2 ‰, $\delta^{13}C$ = -5.01 ‰, VPDB) and an in-house carbonate standard, Carrera Lago Marble (CLM $\delta^{18}O$ = -3.66 ‰, $\delta^{13}C$ = 2.31 ‰, VPDB). The CLM standard has been calibrated alongside NBS-19 and IAEA-603. A two-point calibration using CLM and NBS-18 was employed for the analyses presented here. Analytical uncertainties ($2\sigma_{SD}$) for these analyses are ±0.2‰ for $\delta^{18}O_{carb}$ and ±0.1‰ for $\delta^{13}C_{carb}$.

Elemental analysis

Approximately 50 mg of powdered shell was placed in acid-washed HDPE test tubes and dissolved in ~10 mL of 5 wt% HNO_3. The mixtures were agitated on a rocker table for ~24 hours, centrifuged, and then filtered through 0.45 µm syringe filters. Solutions were diluted with 5 wt% HNO_3 to ensure elemental concentrations were within calibration ranges of standards

(assuming near-to-pure calcium carbonate). Measurements were done using a Thermo Scientific iCAP 6500 ICP-OES using Argon carrier gas and a Cetac U-6000AT+ Ultrasonic Nebulizer in the Aqueous Geochemistry Laboratory at Northwestern University. Repeated measurements of NIST SRM 1643f were used to both monitor instrument stability and confirm data quality for Ca, Mg, Mn, Na, and Sr. Three replicates of 3mL of solution were averaged for standards, blanks, and samples. Instrument drift was monitored using a standard, sample, standard bracketing technique with no more than 15 samples analyzed between standards. Standard measurements suggest an instrumental precision of ±5% (RSD, relative standard deviation) for each element (Ca ~0.5 ppm , Mg ~0.1 ppb, Mn ~0.1 ppb, Na ~6 ppb, and Sr ~5 ppb).

Carbonate $\delta^{44/40}$Ca

Calcium isotope values were measured in the Radiogenic Isotope Laboratory at Northwestern University using an optimized ^{43}Ca-^{42}Ca double spike method implemented on a Thermo-Fisher Triton Multi-Collector Thermal Ionization Mass Spectrometer (TIMS) [41]. Sample solutions containing 50 µg of Ca were weighed into acid-cleaned Teflon vials, spiked, and equilibrated. Solutions were dried down overnight at 90 °C. Residues were then dissolved in 0.5 mL of 1.55N HCl, and Ca was separated from other elements by passing solutions through Teflon columns filled with Bio-Rad AG MP-50 cation exchange resin. Solutions were dried down once more and subsequently treated with two drops of 35 wt% H_2O_2 to oxidize organic compounds and finally two drops of 16N HNO_3 to convert Ca to nitrate form. Samples were then dissolved in 0.4 µL of 8N HNO_3 and split into 4 beads. One bead was loaded onto a single degassed tantalum filament assembly, between ~0.5 mm wide parafilm dams, and subsequently dried at 3.5 amps after adding 0.5 µL of 10 wt% H_3PO_4.

A stable, 20V ^{40}Ca ion beam was obtained after a 0.5 hr warm-up sequence. Measurement of $^{40}Ca/^{42}Ca$, $^{43}Ca/^{42}Ca$, and $^{43}Ca/^{44}Ca$ ratios was accomplished using a three-hop collector cup configuration. Sample (SMP) $^{44}Ca/^{40}Ca$ ratios are reported in delta notation relative to OSIL seawater (SW), where $\delta^{44/40}Ca_{SMP} = [(^{44}Ca/^{40}Ca)_{SMP} / (^{44}Ca/^{40}Ca)_{SW} - 1] \times 1000$. Analyses of the OSIL SW standard yielded a reproducibility of ±0.04‰ ($2\sigma_{SD}$) during the analytical sessions including these samples. Analyses of NIST 915b produced an average $\delta^{44/40}Ca$ value of -1.14‰ ± 0.07‰ ($2\sigma_{SD}$) during the duration of the study. All stable isotope and elemental data are available in the Supplemental data file (SI 1).

Difference estimates

The difference estimates between smaller subsets of data are based on a Monte Carlo approach. We add a random error term to each analysis assuming the instrumental precision describes two standard deviations of a normal distribution for expected variability around our measured values. We then calculate the difference between groups for this new dataset and repeat this operation 1,000,000 times. Error estimates are reported as the 5th to 95th percentiles of the differences between the groups from the Monte Carlo method.

Results

Carbonate trace elements

Recent samples show no discernible temporal trends in either Mg/Ca or Sr/Ca ratios and display similar variation to the subfossil specimens (Fig 4). Material from subfossil cenote specimens has higher Sr/Ca (3.0 mmol/mol) and lower Mg/Ca (0.5 mmol/mol) than most historical samples (Fig 4).

Fig 4. Crossplots of Sr/Ca and Mg/Ca ratios vs isotope values measured on shell carbonate. Error bars are 2 standard deviation instrumental precision for isotope values and the 5% RSD propagated to the elemental ratios. A) $\delta^{44/40}$Ca vs Mg/Ca, B) $\delta^{44/40}$Ca vs Sr/Ca, C) $\delta^{13}C_{shell}$ vs Mg/Ca, D) $\delta^{13}C_{shell}$ vs Sr/Ca, E) $\delta^{18}O_{shell}$ vs Mg/Ca, F) $\delta^{18}O_{shell}$ vs Mg/Ca, G) δ^{15}N vs Mg/Ca, H) δ^{15}N vs Sr/Ca, I) $\delta^{13}C_{org}$ vs Mg/Ca, and J) $\delta^{13}C_{org}$ vs Sr/Ca. The cenote and historical samples distinctly differ along Mg/Ca, Sr/Ca, δ^{15}N, $\delta^{13}C_{org}$, and $\delta^{44/40}$Ca axes.

Carbonate isotopes

The cenote specimens fall within the range of δ^{18}O and δ^{13}C of shell carbonate measured for modern specimens (Fig 5). No change in $\delta^{44/40}$Ca is apparent in these samples less than 120 years old; however, the subfossil cenote specimens are 0.12‰ lower (0.11 to 0.15‰) than modern samples (Fig 6 A). For samples collected in the last 120 years, $\delta^{13}C_{shell}$ decreases by 2.09‰ (2.03 to 2.15‰) toward the present (Fig 6 B), while $\delta^{18}O_{shell}$ increases by 0.32‰ (0.45 – 0.20‰) (Fig 6 C).

Fig 5. Crossplot of carbon and oxygen isotope values measured on shell carbonate. Error bars are 2 standard deviation instrumental precision.

Fig 6. Stable isotope values through time. Error bars are 2 standard deviation instrumental precision. A) Shell $\delta^{44/40}$Ca through time. B) The δ^{13}C of shell carbonate through time. C) The δ^{18}O of shell carbonate through time. D) The δ^{15}N of shell organic matter though time. E) The δ^{13}C of shell organic matter through time.

Organic matter isotopes

Samples spanning the last 130 years show no temporal trends in either $\delta^{13}C_{org}$ or $\delta^{15}N_{org}$ (Fig 6 D and E and Fig 7). Subfossil cenote specimens have lower $\delta^{13}C_{org}$ and $\delta^{15}N_{org}$ by 6.06‰ (6.25 to 5.87‰) and 1.74‰ (2.03 to 1.45‰), respectively. Recent samples also consistently have lower C/N (<3) compared to subfossil specimens (>4, Fig 7 B).

Fig 7. Isotope values of organic matter and the C/N ratio of the organic matter. Error bars are 2 standard deviation instrumental precision. A) $\delta^{13}C_{org}$ vs $\delta^{15}N_{org}$, B) C/N vs $\delta^{15}N_{org}$.

Discussion

Diagenesis

Elemental ratios

Elemental ratios of biogenic carbonates are vulnerable to diagenetic alteration. Increases in Sr/Ca from 2 mmol/mol to 5 mmol/mol have been correlated with microstructural diagenetic alteration of fossil cephalopod shell and correspond to change in $^{87}Sr/^{86}Sr$, $\delta^{18}O_{carb}$, and $\delta^{13}C_{carb}$. The cenote samples do not appear to be outliers in either Sr/Ca or Mg/Ca compared to modern samples, however. This suggests that diagenetic alteration of Sr/Ca and Mg/Ca is not detectable in the cenote samples.

Organic matter isotopes

Although organic matter within mollusk shells has been reported for multiple fossil specimens, pristine preservation is rare [42–45]. During dissolution of these shells for isolation of organic matter, it is apparent that cenote specimens have considerably less organic matter than modern specimens, but the effects on the isotope composition of residual organic matter is

important to explore. Degradation of organic matter within shells generally removes more labile organic matter (e.g. amino acids, glycoproteins, sugars) and preserves refractory organic matter, although the exact effect on $\delta^{13}C_{org}$ and $\delta^{15}N_{org}$ values is unknown. Two sources of organic matter in the shell, carbonate-bound organic matter (intracrystalline) and non-carbonate bound organic matter (intercrystalline), likely behave differently during diagenesis due to the differing volumes and connectedness of the organic matter for microbial degradation. Some authors have noted much lower $\delta^{15}N$ (1-4‰) in significantly older fossil nautiloids (*Cymatoceras sakalavus*, Albian ~113 to 100 Ma) and have suggested these low $\delta^{15}N$ values reflect diagenetic alteration [14]. Sedimentary organic matter degradation drives $\delta^{15}N$ toward lower values while C/N increases [46]. We see a similar pattern between the recent and subfossil specimens (Fig 7 B) and therefore suggest that the $\delta^{15}N$ of the cenote specimens is altered and does not reflect a primary trophic level signature. The $\delta^{13}C_{org}$ values in these samples are also lower than the more recent specimens (Fig 7 A). Diagenetic alteration of $\delta^{13}C_{org}$ in mollusks also likely drives isotope values lower due to a loss of the hydrophilic fraction of organic matter and potentially transformation to hydrophobic organic matter. The transformation involves preferentially losing ^{13}C by not reincorporating it in hydrophobic organic matter.

Carbonate isotopes

Diagenetic alteration of light stable isotopes is of particular interest for constraining the fidelity of paleoclimatological records [47–49]. The subfossil specimens from the cenote show signs that shell nacre recrystallized [34], and higher Sr/Ca in these shells is consistent with addition of either strontianite [47] or authigenic aragonite (Fig 4B). The lack of clear $\delta^{13}C$ or $\delta^{18}O$ outliers is notable and suggests that any recrystallization did not incorporate large volumes of additional diagenetic carbonate. Shell $\delta^{44/40}Ca$ may have been altered toward lower values, as

has been observed in Cretaceous mollusks from Antarctica [21], where trends toward higher Sr/Ca (5 up to 12 mmol/mol) correlate with lower $\delta^{44/40}$Ca values (from -1.5 to -2‰). This could point to the addition of small domains of authigenic aragonite or strontianite, but the variation observed in the Sr/Ca in the most recent *Nautilus m.* (Fig 4) suggests that the pattern may not be consistent with diagenesis.

If any diagenesis occurred, it happened at low temperatures (~28 °C), ambient pressures, and within the last 7000 years [34] given the location of the cenote and previously published radiocarbon ages. Carbonate recrystallization/precipitation was likely tied to microbial degradation of organic matter causing variable carbonate saturation state within or near shells [50,51]. Future work contrasting these diagenetic pathways with higher temperature hydrothermal experiments [52,53] may help provide a more detailed ‘taphonomic chronometer’ for use in deep time [54].

Isotope and elemental interpretation

Elemental ratios

The elemental ratios of *Nautilus* shell potentially reflect taxonomic or geographic differences rather than temperatures, as has been observed in bivalve mollusks. We observe a wide range of Sr/Ca and Mg/Ca across these samples with no clear trend towards modern samples. This variation within modern specimens suggests that more work on Mg/Ca and Sr/Ca variation within *Nautilus* is needed to understand the limits of intraspecific variation and potential thresholds for identification of diagenetic alteration.

Organic matter isotopes

Modern and historical shells suggest slight variation in prey preference or availability for the individuals measured (Fig 7 A). In other work on *Nautilus*, $\delta^{15}N_{org}$ changes with hatching and early growth and variation of ~1-2‰ within and between individuals has been observed [14]. Similar individual – to – individual and within individual variability may be linked to differing prey choice [55,56] and is likely to be present in *Nautilus* given neurological complexity [57]. There are no strong trends through time in the recent $\delta^{13}C_{org}$ or $\delta^{15}N_{org}$ (Fig 6 D and E and Fig 7 A) that would suggest major trophic changes for these *Nautilus m.* in the last ~120 years. Future work using compound specific $\delta^{15}N_{org}$ analysis of amino acids within samples like these could support trophic level stability.

Calcium isotopes

Previous studies have proposed that $\delta^{44/40}Ca$ can indicate trophic level for vertebrates in modern and ancient terrestrial and marine ecosystems [58,59]. Ingestion of hard tissue material and subsequent use of calcium from that source in biomineralization is likely necessary to cause a $\delta^{44/40}Ca$ trophic level effect [60]. It is possible that the difference measured between the oldest *Nautilus m.* and the more recent samples (Figs 6 A and 8 F) reflects changing composition of scavenged prey, including crustaceans and fish [61], perhaps due to ecosystem changes coupled to fishing pressures [62,63]. Shifting dietary composition causing different $\delta^{44/40}Ca$ is an unlikely explanation, however, because no strong correlation with either $\delta^{15}N_{org}$ or $\delta^{13}C_{org}$ is observed, and moreover, recent individuals show measurable differences in both isotope systems indicative of differing diets (Fig 8 F [55]). In addition, comparing $\delta^{44/40}Ca$ to average $\delta^{15}N$ and trophic level across other modern *Nautilus*, *Sepia*, and *Spirula* suggests a lack of trophic response in cephalopods [23].

Fig 8. Crossplots of isotope values measured in shell organic matter vs shell carbonate. Error bars are 2 standard deviation instrumental precision. A) $\delta^{13}C_{org}$ vs $\delta^{13}C_{shell}$, B) $\delta^{15}N$ vs $\delta^{13}C_{shell}$, C) $\delta^{13}C_{org}$ vs $\delta^{18}O_{shell}$, D) $\delta^{15}N$ vs $\delta^{18}O_{shell}$, E) $\delta^{13}C_{org}$ vs $\delta^{44/40}Ca$, and F) $\delta^{15}N$ vs $\delta^{44/40}Ca$.

The calcium isotope composition of mollusk material may vary with the calcium isotope composition of seawater ($\delta^{44/40}Ca_{SW}$) or changes in the fractionation between seawater and precipitated shell ($\Delta^{44/40}Ca_{SW\text{-}Shell}$) [21]. Bivalve mollusks derive ~80% or more of their shell calcium from seawater sources [64] and can make use of only water sources for 100% of calcium in shell precipitation [65]. While shifts in local-scale Ca cycling dynamics are possible [22], changes in the isotopic composition of the global ocean unlikely explain the observed variations, given the long residence time of Ca in seawater. Evidence from the geologic record, however, suggests that $\Delta^{44/40}Ca_{SW\text{-}Shell}$ is sensitive to seawater carbonate state. Culturing experiments show that the pH of extrapallial fluid from *Arctica islandica* bivalves is at least partially regulated during shell formation, but can vary with ambient pH [66]. If *Nautilus* similarly regulates the pH and potentially the saturation state of extrapallial fluid during shell precipitation, then variation in $\Delta^{44/40}Ca_{SW\text{-}Shell}$ would be driven by mass-dependent isotope effects during transport into the extrapallial fluid rather than with varying saturation state and precipitation rate at the site of precipitation, as has been observed in inorganic systems. Temperature may affect $\Delta^{44/40}Ca_{SW\text{-}Shell}$ in bivalves [67], although temperature covaries with carbonate saturation state, which can be modulated by the ratio of photosynthesis to respiration [68,69], and in general, the temperature sensitivity of $\Delta^{44/40}Ca_{SW\text{-}Shell}$ for other carbonate producers is low [20,70–73].

Seawater likely constitutes the largest source of calcium for the precipitation of *Nautilus m.* shell [64,65], but complex precipitation and resorption of intermediate carbonate reservoirs could influence shell $\delta^{44/40}Ca$ [74]. Uroliths represent one proposed intermediary carbonate

structure between seawater and shell precipitation [75]. These structures are amorphous calcium phosphate and have higher Sr contents than the shell wall [76]. The calcium fractionation factor for this material is unknown, but may be close to -1‰, similar to peloidal calcium phosphate [77,78].

Fixed $\Delta^{44/40}Ca_{SW\text{-}Shell}$ in cephalopods (mostly for belemnites) is typically assumed in the reconstruction of $\delta^{44/40}Ca_{SW}$ change through time [23,79–81]. Recent evidence suggests that $\Delta^{44/40}Ca_{SW\text{-}Shell}$ for other types of carbonate producers varies with seawater saturation state [21,82,83] in a way consistent with theoretical models for inorganic calcite formation [84,85]. Any variation of cephalopod $\Delta^{44/40}Ca_{SW\text{-}Shell}$ may reflect additional controls, such as changes in the transport of Ca into the extrapallial fluid (EPF). One possibility is that proportions of Ca transported by selective intracellular calcium channels, non-selective intercellular pathways, or active enzymatic transport (Ca^{2+}-ATPase and carbonic anhydrase) can change. However, under most conditions, selective intracellular calcium channels are thought to dominate transportation of Ca into the EPF through high diffusive fluxes that are selective for Ca [86]. Another possibility is that any Ca isotope fractionation imparted by one or more of these transport mechanisms varies with seawater saturation state. Future well-controlled aquarium rearing experiments will be necessary to determine if $\Delta^{44/40}Ca_{SW\text{-}Shell}$ changes with carbonate saturation state or another environmental condition.

For anthropogenic ocean acidification to change $\Delta^{44/40}Ca$ of these *Nautilus*, higher CO_2 concentrations must propagate to their living depths, which in turn must remain relatively stable throughout the study interval. The observed change in $\delta^{13}C$ of shell carbonate (Fig 6B) suggests propagation of anthropogenic carbon into the habitat of the *Nautilus* but does not guarantee a change in the concentration of CO_2 necessary to cause acidification. Boron isotope

measurements from shallow water (7-8 m) coral suggests a decrease of ~0.15 pH near New Caledonia since the 1890's, but also indicate similar variations on decadal timescales [87]. Deeper waters, like those that the *Nautilus* inhabit, are generally expected to acidify more slowly, although organic matter export can sometimes enhance acidification at depth depending on the interaction between deep water ventilation, organic matter export, and general ocean circulation patterns [88,89]. Because the oceans are still in the early stages of acidification, it is likely that the amount of pH change at these depths has not been sufficient to cause detectable alteration in the $\delta^{44/40}$Ca of the studied *Nautilus* specimens.

Respired CO_2

The proportion of respired carbon incorporated into the shells of mollusks depends on changes in ambient O_2:CO_2 in surrounding waters [13,90] and the metabolic rate [91]. Use of the proxy assumes that all carbon used for the precipitation of biogenic carbonates derives from either seawater dissolved inorganic carbon (DIC) or respired CO_2 derived from food. The δ^{13}C metabolic rate proxy has been explored using aquarium-reared cephalopods, including *Sepia pharaonis* [92,93]. The quantity of respired carbon contributing to carbonate precipitation can be calculated following a simple relationship outlined by [13,90]:

$$C_{metabolic} = \frac{\delta^{13}C_{shell} - \varepsilon_{aragonite} - \delta^{13}C_{DIC}}{\delta^{13}C_{metabolic} - \delta^{13}C_{DIC}} \qquad (1)$$

where $\delta^{13}C_{shell}$ refers to the measured value for carbonate shell material. We assume $\delta^{13}C_{metabolic}$ is equivalent to $\delta^{13}C_{org}$ measured in the shell organic matter—given similar findings for other mollusks [16]. The value of $\varepsilon_{aragonite}$ is assumed to be 2.7‰, which is the value for inorganic aragonite [94]. To constrain $\delta^{13}C_{DIC}$, we use published estimates of $\delta^{13}C_{DIC}$ for preindustrial (1.2‰, VPDB) and recent (1.0‰, VPDB) water near New Caledonia [95,96] at the average

Nautilus m. living depth of ~400 m [97]. This depth is chosen even though shell grows across a range of at least 400 m water depth [12] due to time averaging of bulk samples like those used in this study [98]. We assume no ontogenetic change in $\delta^{13}C_{carb}$ biases our results. All samples were from near at the aperture of the body chamber of large shells, and most change in $\delta^{13}C_{carb}$ has been observed in the shell near to hatching rather than later in ontogeny, as is observed in bivalves.

Calculations using these metrics show increasing amounts of respired carbon are being incorporated into the shells of *Nautilus m.* during the last 130 years (Fig 9). The observed increase (from ~8 to ~22%) likely reflects changing metabolic rates caused by transgenerational acclimation to ocean acidification, as has been seen in *Mya arenaria* bivalves [91]. The increase is much less than the observed metabolic carbon increase (27% to 61%) in the experimentally reared bivalves over what is likely to be a much larger pH range (8.1 to 7.7 pH) [91]. Other experimentation with *Arctica islandica* bivalves has suggested that ontogenetic variation in the amount of respired carbon incorporated into shells is minimal [99]. Because the *Nautilus* live relatively deep, CO_2 incorporation from the atmosphere is likely to be lower than that experienced by shallower organisms. The decrease of pH at the living depths of these *Nautilus m.* may be facilitated by dampened deep water oxygenation related to continued organic matter export [88]. The observed increase in respired carbon could also be modulated by changes in the ratio of dissolved oxygen to carbon dioxide through expanding oxygen minimum zones offshore of New Caledonia due to increased agricultural intensity and nutrient-rich runoff [100,101], dramatic changes to vegetation [102], increasing [CO_2] due to anthropogenic injection into the atmosphere-ocean system [103], pressure from mining runoff [104], or changing preferred habitat depths due to changing ecosystem composition from fishing other organisms (Fig 3). It is

also unlikely that the observed increase in metabolic carbon is caused by ontogenetic change (e.g., because shells represent mature *Nautilus* and most $\delta^{13}C_{carb}$ change occurs very early in ontogeny.

Fig 9. Crossplot of the calculated metabolic carbon contribution vs time. Note the increase in the amount of metabolic carbon incorporated into the shell since the 1970's.

Future work

One of the primary challenges to clearly calibrating geochemical proxies in biogenic carbonates is the 'shock' of changing environmental conditions during the experimental treatment of individuals [105,106]. Although the magnitude of anthropogenic and natural environmental change can be large, both are slow relative the generational timescale of organisms. This means that the best calibrations for proxies preserved in biogenic carbonates may be found in historical or spatially distributed samples that inhabited conditions across several generations [29,105]. Studies in modern settings may benefit from cross-calibration with low-cost real-time environmental logging [107]. Focus on mollusk shell is of particular merit due to wide ranging methods for diagenetic assessment [47,48,53,108,109] and preservation of shell organic matter [43,44]. Future work on these isotope systems in cephalopods and other mollusks should leverage museum collections spanning collection time and geographic range to avoid confounding factors of shock and to allow for adaptive responses. Further work on internally shelled mollusks (*Sepia* and *Spirula*) may be informative for interrogating belemnite archives given newfound microstructural complexity [110,111].

Conclusions

Geochemical analyses of subfossil, historical, and modern *Nautilus m.* show the importance of calibrating geochemical signals across generations of organisms. The low temperature, potential diagenetic alteration also points to the importance of robust diagenetic assessment of samples from different environments. Our data suggest that there is intergenerational increase in *Nautilus m.* metabolism near New Caledonia, which is likely caused by ocean acidification. Future work using historical and modern marine mollusk shell material will be important for calibrating geochemical proxies across events that can cause both selection and/or phenotypic plasticity response.

Acknowledgements

We would like to thank Jochen Gerber (Field Museum) and Bushra Hussaini (AMNH) for assistance in accessing samples. We also thank three anonymous reviewers for their constructive comments to improve this manuscript.

References

1. Price GD, Twitchett RJ, Wheeley JR, Buono G. Isotopic evidence for long term warmth in the Mesozoic. Sci Rep. 2013;3. doi:10.1038/srep01438

2. Urey HC, Lowenstam HA, Epstein S, McKinney CR. Measurement of paleotemperatures and temperatures of the Upper Cretaceous of England, Denmark, and the Southeastern United States. GSA Bull. 1951;62: 399–416. doi:10.1130/0016-7606(1951)62[399:MOPATO]2.0.CO;2

3. Ellis NM, Tobin TS. Evidence for seasonal variation in $\delta^{13}C$ and $\delta^{18}O$ profiles of *Baculites* and implications for growth rate. Palaeontology. 2019;62: 583–598. doi:10.1111/pala.12416

4. Ferguson K, Macleod KG, Landman NH, Sessa JA. Evaluating growth and ecology in baculitid and scaphitid ammonites using stable isotope sclerochronology. PALAIOS. 2019;34: 317–329. doi:10.2110/palo.2019.005

5. Landman NH, Cochran JK, Slovacek M, Larson NL, Garb MP, Brezina J, et al. Isotope sclerochronology of ammonites (*Baculites compressus*) from methane seep and non-seep sites in the Late Cretaceous Western Interior Seaway, USA: Implications for ammonite habitat and mode of life. Am J Sci. 2018;318: 603–639. doi:10.2475/06.2018.01

6. Linzmeier BJ, Landman NH, Peters SE, Kozdon R, Kitajima K, Valley JW. Ion microprobe–measured stable isotope evidence for ammonite habitat and life mode during early ontogeny. Paleobiology. 2018;44: 684–708. doi:10.1017/pab.2018.21

7. Lukeneder A, Harzhauser M, Müllegger S, Piller WE. Ontogeny and habitat change in Mesozoic cephalopods revealed by stable isotopes ($\delta^{18}O$, $\delta^{13}C$). Earth Planet Sci Lett. 2010;296: 103–114. doi:10.1016/j.epsl.2010.04.053

8. Moriya K, Nishi H, Kawahata H, Tanabe K, Takayanagi Y. Demersal habitat of Late Cretaceous ammonoids: Evidence from oxygen isotopes for the Campanian (Late Cretaceous) northwestern Pacific thermal structure. Geology. 2003;31: 167–170.

9. Sessa JA, Larina E, Knoll K, Garb M, Cochran JK, Huber BT, et al. Ammonite habitat revealed via isotopic composition and comparisons with co-occurring benthic and planktonic organisms. Proc Natl Acad Sci. 2015;112: 15562–15567. doi:10.1073/pnas.1507554112

10. Kröger B, Vinther J, Fuchs D. Cephalopod origin and evolution: A congruent picture emerging from fossils, development and molecules. BioEssays. 2011;33: 602–613. doi:10.1002/bies.201100001

11. Landman NH, Cochran JK, Rye DM, Tanabe K, Arnold JM. Early life history of *Nautilus*: evidence from isotopic analyses of aquarium-reared specimens. Paleobiology. 1994;20: 40–51.

12. Linzmeier BJ, Kozdon R, Peters SE, Valley JW. Oxygen isotope variability within *Nautilus* shell growth bands. PLOS ONE. 2016;11: e0153890. doi:10.1371/journal.pone.0153890

13. McConnaughey TA, Gillikin DP. Carbon isotopes in mollusk shell carbonates. Geo-Mar Lett. 2008;28: 287–299. doi:10.1007/s00367-008-0116-4

14. Kashiyama Y, Ogawa NO, Chikaraishi Y, Kashi N, Sakai S, Tanabe K, et al. Reconstructing the life history of modern and fossil nautiloids based on the nitrogen isotopic composition of shell organic matter and amino acids. In: Tanabe K, Shigeta Y, Sasaki T, Hirano H, editors. Cephalopods - Present and Past. Tokyo: Tokai University Press; 2010. pp. 67–75.

15. Crocker KC, DeNiro MJ, Ward PD. Stable isotopic investigations of early development in extant and fossil chambered cephalopods I. Oxygen isotopic composition of eggwater and carbon isotopic composition of siphuncle organic matter in Nautilus. Geochim Cosmochim Acta. 1985;49: 2527–2532. doi:10.1016/0016-7037(85)90120-6

16. Gillikin DP, Lorrain A, Meng L, Dehairs F. A large metabolic carbon contribution to the $\delta^{13}C$ record in marine aragonitic bivalve shells. Geochim Cosmochim Acta. 2007;71: 2936–2946. doi:10.1016/j.gca.2007.04.003

17. Ward PD. Periodicity of chamber formation in chambered cephalopods: Evidence from *Nautilus macromphalus* and *Nautilus pompilius*. Paleobiology. 1985;11: 438–450.

18. Mutvei H. Ultrastructural relationships between the prismatic and nacreous layers in *Nautilus* (Cephalopoda). Biomineralization. 1972;4: 81–86.

19. Mutvei H. On the shells of *Nautilus* and *Spirula*: with notes on shell secretion in non-cephalopod molluscs. Ark För Zool. 1964;16: 221–278.

20. Gussone N, Heuser A. Biominerals and Biomaterial. Calcium Stable Isotope Geochemistry. Berlin, Heidelberg: Springer Berlin Heidelberg; 2016. pp. 111–144. doi:10.1007/978-3-540-68953-9_4

21. Linzmeier BJ, Jacobson AD, Sageman BB, Hurtgen MT, Ankney ME, Petersen SV, et al. Calcium isotope evidence for environmental variability before and across the Cretaceous-Paleogene mass extinction. Geology. 2020;48: 34–38. doi:10.1130/G46431.1

22. Holmden C, Papanastassiou DA, Blanchon P, Evans S. $\delta^{44/40}Ca$ variability in shallow water carbonates and the impact of submarine groundwater discharge on Ca-cycling in marine environments. Geochim Cosmochim Acta. 2012;83: 179–194. doi:10.1016/j.gca.2011.12.031

23. Hoffmann R, Riechelmann S, Liebetrau V, Eisenhauer A, Immenhauser A. Calcium isotope values of modern and fossil cephalopod shells – Trophic level or proxy for seawater geochemistry? Chem Geol. 2021;583: 120466. doi:10.1016/j.chemgeo.2021.120466

24. Fennel K, Testa JM. Biogeochemical Controls on Coastal Hypoxia. Annu Rev Mar Sci. 2019;11: 105–130. doi:10.1146/annurev-marine-010318-095138

25. Barord GJ, Dooley F, Dunstan A, Ilano A, Keister KN, Neumeister H, et al. Comparative Population Assessments of *Nautilus* sp. in the Philippines, Australia, Fiji, and American Samoa Using Baited Remote Underwater Video Systems. PLoS ONE. 2014;9: e100799. doi:10.1371/journal.pone.0100799

26. Doney SC, Fabry VJ, Feely RA, Kleypas JA. Ocean Acidification: The Other CO2 Problem. Annu Rev Mar Sci. 2009;1: 169–192. doi:10.1146/annurev.marine.010908.163834

27. Kroeker KJ, Kordas RL, Harley CDG. Embracing interactions in ocean acidification research: confronting multiple stressor scenarios and context dependence. Biol Lett. 2017;13: 20160802. doi:10.1098/rsbl.2016.0802

28. Schunter C, Welch MJ, Nilsson GE, Rummer JL, Munday PL, Ravasi T. An interplay between plasticity and parental phenotype determines impacts of ocean acidification on a reef fish. Nat Ecol Evol. 2018;2: 334–342. doi:10.1038/s41559-017-0428-8

29. Telesca L, Peck LS, Backeljau T, Heinig MF, Harper EM. A century of coping with environmental and ecological changes via compensatory biomineralization in mussels. Glob Change Biol. 2021;27: 624–639. doi:https://doi.org/10.1111/gcb.15417

30. Urban MC, Bocedi G, Hendry AP, Mihoub J-B, Pe'er G, Singer A, et al. Improving the forecast for biodiversity under climate change. Science. 2016;353. doi:10.1126/science.aad8466

31. Kidwell SM. Time-averaged molluscan death assemblages: Palimpsests of richness, snapshots of abundance. Geology. 2002;30: 803–806. doi:10.1130/0091-7613(2002)030<0803:TAMDAP>2.0.CO;2

32. Kowalewski M, Casebolt S, Hua Q, Whitacre KE, Kaufman DS, Kosnik MA. One fossil record, multiple time resolutions: Disparate time-averaging of echinoids and mollusks on a Holocene carbonate platform. Geology. 2018;46: 51–54. doi:10.1130/G39789.1

33. Stevenson J, Dodson JR, Prosser IP. A late Quaternary record of environmental change and human impact from New Caledonia. Palaeogeogr Palaeoclimatol Palaeoecol. 2001;168: 97–123. doi:10.1016/S0031-0182(00)00251-0

34. Landman NH, Mapes RH, Cochran JK, Lignier V, Hembree DI, Goiran C, et al. An unusual occurrence of *Nautilus macromphalus* in a cenote in the Loyalty Islands (New Caledonia). PLOS ONE. 2014;9: e113372. doi:10.1371/journal.pone.0113372

35. House MR. Geographic Distribution of Nautilus Shells. In: Saunders WB, Landman NH, editors. Nautilus: The Biology and Paleobiology of a Living Fossil, Reprint with additions. Dordrecht: Springer Netherlands; 2010. pp. 53–64. doi:10.1007/978-90-481-3299-7_4

36. Cravatte S, Kestenare E, Eldin G, Ganachaud A, Lefèvre J, Marin F, et al. Regional circulation around New Caledonia from two decades of observations. J Mar Syst. 2015;148: 249–271. doi:10.1016/j.jmarsys.2015.03.004

37. Etheridge DM, Steele LP, Langenfelds RL, Francey RJ, Barnola JM, Morgan VI. Historical CO2 records from the Law Dome DE08, DE08-2, and DSS ice cores. Trends Compend Data Glob Change. 1998; 351–364.

38. Saunders WB. The Species of *Nautilus*. Nautilus. Springer, Dordrecht; 2010. pp. 35–52. doi:10.1007/978-90-481-3299-7_3

39. Landman NH, Cochran JK. Growth and Longevity of *Nautilus*. Nautilus. Springer, Dordrecht; 2010. pp. 401–420. doi:10.1007/978-90-481-3299-7_28

40. Schimmelmann A, Albertino A, Sauer PE, Qi H, Molinie R, Mesnard F. Nicotine, acetanilide and urea multi-level ^{2}H-, ^{13}C- and ^{15}N-abundance reference materials for continuous-flow isotope ratio mass spectrometry. Rapid Commun Mass Spectrom. 2009;23: 3513–3521. doi:https://doi.org/10.1002/rcm.4277

41. Lehn GO, Jacobson AD, Holmden C. Precise analysis of Ca isotope ratios ($\delta^{44/40}Ca$) using an optimized ^{43}Ca–^{42}Ca double-spike MC-TIMS method. Int J Mass Spectrom. 2013;351: 69–75. doi:10.1016/j.ijms.2013.06.013

42. Buchardt B, Weiner S. Diagenesis of aragonite from Upper Cretaceous ammonites: a geochemical case-study. Sedimentology. 1981;28: 423–438. doi:10.1111/j.1365-3091.1981.tb01691.x

43. Clark GRI. Organic matrix taphonomy in some molluscan shell microstructures. Palaeogeogr Palaeoclimatol Palaeoecol. 1999;149: 305–312.

44. Myers CE, Bergmann KD, Sun C-Y, Boekelheide N, Knoll AH, Gilbert PUPA. Exceptional preservation of organic matrix and shell microstructure in a Late Cretaceous *Pinna* fossil revealed by photoemission electron spectromicroscopy. Geology. 2018;46: 711–714. doi:10.1130/G45271.1

45. Weiner S, Lowenstam HA, Hood L. Characterization of 80-million-year-old mollusk shell proteins. Proc Natl Acad Sci. 1976;73: 2541–2545. doi:10.1073/pnas.73.8.2541

46. Junium CK, Arthur MA. Nitrogen cycling during the Cretaceous, Cenomanian-Turonian Oceanic Anoxic Event II. Geochem Geophys Geosystems. 2007;8. doi:10.1029/2006GC001328

47. Cochran JK, Kallenberg K, Landman NH, Harries PJ, Weinreb D, Turekian KK, et al. Effect of diagenesis on the Sr, O, and C isotope composition of Late Cretaceous mollusks from the Western Interior Seaway of North America. Am J Sci. 2010;310: 69–88. doi:10.2475/02.2010.01

48. Immenhauser A, Schöne BR, Hoffmann R, Niedermayr A. Mollusc and brachiopod skeletal hard parts: Intricate archives of their marine environment. Sedimentology. 2016;63: 1–59. doi:10.1111/sed.12231

49. Swart PK. The geochemistry of carbonate diagenesis: The past, present and future. Sedimentology. 2015;62: 1233–1304. doi:10.1111/sed.12205

50. Eyre BD, Cyronak T, Drupp P, Carlo EHD, Sachs JP, Andersson AJ. Coral reefs will transition to net dissolving before end of century. Science. 2018;359: 908–911. doi:10.1126/science.aao1118

51. Ries JB, Ghazaleh MN, Connolly B, Westfield I, Castillo KD. Impacts of seawater saturation state (ΩA=0.4–4.6) and temperature (10, 25°C) on the dissolution kinetics of whole-shell biogenic carbonates. Geochim Cosmochim Acta. 2016;192: 318–337. doi:10.1016/j.gca.2016.07.001

52. Milano S, Prendergast AL, Schöne BR. Effects of cooking on mollusk shell structure and chemistry: Implications for archeology and paleoenvironmental reconstruction. J Archaeol Sci Rep. 2016;7: 14–26. doi:10.1016/j.jasrep.2016.03.045

53. Pederson C, Mavromatis V, Dietzel M, Rollion-Bard C, Nehrke G, Jöns N, et al. Diagenesis of mollusc aragonite and the role of fluid reservoirs. Earth Planet Sci Lett. 2019;514: 130–142. doi:10.1016/j.epsl.2019.02.038

54. Tomašových A, Schlögl J, Biroň A, Hudáčková N, Mikuš T. Taphonomic clock and bathymetric dependence of cephalopod preservation in bathyal, sediment-starved environments. PALAIOS. 2017;32: 135–152. doi:10.2110/palo.2016.039

55. Newsome SD, Yeakel JD, Wheatley PV, Tinker MT. Tools for quantifying isotopic niche space and dietary variation at the individual and population level. J Mammal. 2012;93: 329–341. doi:10.1644/11-MAMM-S-187.1

56. Newsome SD, Tinker MT, Monson DH, Oftedal OT, Ralls K, Staedler MM, et al. Using Stable Isotopes to Investigate Individual Diet Specialization in California Sea Otters (*Enhydra lutris nereis*). Ecology. 2009;90: 961–974.

57. Young JZ. The Central Nervous System. In: Saunders WB, Landman NH, editors. Nautilus: The Biology and Paleobiology of a Living Fossil, Reprint with additions. Dordrecht: Springer Netherlands; 2010. pp. 215–221. doi:10.1007/978-90-481-3299-7_13

58. Martin JE, Vincent P, Tacail T, Khaldoune F, Jourani E, Bardet N, et al. Calcium Isotopic Evidence for Vulnerable Marine Ecosystem Structure Prior to the K/Pg Extinction. Curr Biol. 2017;27: 1641-1644.e2. doi:10.1016/j.cub.2017.04.043

59. Skulan J, DePaolo DJ. Calcium isotope fractionation between soft and mineralized tissues as a monitor of calcium use in vertebrates. Proc Natl Acad Sci. 1999;96: 13709–13713. doi:10.1073/pnas.96.24.13709

60. Heuser A, Tütken T, Gussone N, Galer SJG. Calcium isotopes in fossil bones and teeth — Diagenetic versus biogenic origin. Geochim Cosmochim Acta. 2011;75: 3419–3433. doi:10.1016/j.gca.2011.03.032

61. Saunders WB, Ward PD. Ecology, Distribution, and Population Characteristics of *Nautilus*. Nautilus. Springer, Dordrecht; 2010. pp. 137–162. doi:10.1007/978-90-481-3299-7_9

62. Cinner JE, Zamborain-Mason J, Gurney GG, Graham NAJ, MacNeil MA, Hoey AS, et al. Meeting fisheries, ecosystem function, and biodiversity goals in a human-dominated world. 2020; 6.

63. Guillemot N, Léopold M, Cuif M, Chabanet P. Characterization and management of informal fisheries confronted with socio-economic changes in New Caledonia (South Pacific). Fish Res. 2009;98: 51–61. doi:10.1016/j.fishres.2009.03.013

64. Bevelander G. Calcification in mollusks III. Intake and deposition of Ca^{45} and P^{32} in relation to shell formation. Biol Bull. 1952;102: 9–15. doi:10.2307/1538617

65. Borght OVD, Puymbroeck SV. Calcium Metabolism in a Freshwater Mollusc : Quantitative Importance of Water and Food as Supply for Calcium During Growth. Nature. 1966;210: 791. doi:10.1038/210791a0

66. Liu Y-W, Aciego SM, Wanamaker AD. Environmental controls on the boron and strontium isotopic composition of aragonite shell material of cultured *Arctica islandica*. Biogeosciences. 2015;12: 3351–3368. doi:10.5194/bg-12-3351-2015

67. Hippler D, Witbaard R, van Aken HM, Buhl D, Immenhauser A. Exploring the calcium isotope signature of *Arctica islandica* as an environmental proxy using laboratory- and field-cultured specimens. Palaeogeogr Palaeoclimatol Palaeoecol. 2013;373: 75–87. doi:10.1016/j.palaeo.2011.11.015

68. Kelly MW, Hofmann GE. Adaptation and the physiology of ocean acidification. Funct Ecol. 2013;27: 980–990. doi:10.1111/j.1365-2435.2012.02061.x

69. Waldbusser GG, Salisbury JE. Ocean acidification in the coastal zone from an organism's perspective: Multiple system parameters, frequency domains, and habitats. Annu Rev Mar Sci. 2014;6: 221–247. doi:10.1146/annurev-marine-121211-172238

70. Gussone N, Langer G, Thoms S, Nehrke G, Eisenhauer A, Riebesell U, et al. Cellular calcium pathways and isotope fractionation in *Emiliania huxleyi*. Geology. 2006;34: 625–628. doi:10.1130/G22733.1

71. Gussone N, Greifelt T. Incorporation of Ca isotopes in carapaxes of marine ostracods. Chem Geol. 2019;510: 130–139. doi:10.1016/j.chemgeo.2019.02.012

72. Pretet C, Samankassou E, Felis T, Reynaud S, Böhm F, Eisenhauer A, et al. Constraining calcium isotope fractionation (δ44/40Ca) in modern and fossil scleractinian coral skeleton. Chem Geol. 2013;340: 49–58. doi:10.1016/j.chemgeo.2012.12.006

73. Sime NG, De La Rocha CL, Galy A. Negligible temperature dependence of calcium isotope fractionation in 12 species of planktonic foraminifera. Earth Planet Sci Lett. 2005;232: 51–66. doi:10.1016/j.epsl.2005.01.011

74. Boucher-Rodoni R, Boucher G. Respiratory quotient and calcification of *Nautilus macromphalus* (Cephalopoda: Nautiloidea). Mar Biol. 1993;117: 629–633.

75. Schipp R, Martin AW. The Excretory System of Nautilus. In: Saunders WB, Landman NH, editors. Nautilus: The Biology and Paleobiology of a Living Fossil, Reprint with additions. Dordrecht: Springer Netherlands; 2010. pp. 281–304. doi:10.1007/978-90-481-3299-7_20

76. Lowenstam HA, Traub W, Weiner S. *Nautilus* hard parts: a study of the mineral and organic constitutents. Paleobiology. 1984;10: 268–279.

77. Gussone N, Dietzel M. Calcium Isotope Fractionation During Mineral Precipitation from Aqueous Solution. Calcium Stable Isotope Geochemistry. Berlin, Heidelberg: Springer Berlin Heidelberg; 2016. pp. 75–110. doi:10.1007/978-3-540-68953-9_3

78. Schmitt A-D, Stille P, Vennemann T. Variations of the $^{44}Ca/^{40}Ca$ ratio in seawater during the past 24 million years: evidence from $\delta^{44}Ca$ and $\delta^{18}O$ values of Miocene phosphates. Geochim Cosmochim Acta. 2003;67: 2607–2614. doi:10.1016/S0016-7037(03)00100-5

79. Blättler CL, Henderson GM, Jenkyns HC. Explaining the Phanerozoic Ca isotope history of seawater. Geology. 2012;40: 843–846. doi:10.1130/G33191.1

80. Farkaš J, Böhm F, Wallmann K, Blenkinsop J, Eisenhauer A, van Geldern R, et al. Calcium isotope record of Phanerozoic oceans: Implications for chemical evolution of seawater and its causative mechanisms. Geochim Cosmochim Acta. 2007;71: 5117–5134. doi:10.1016/j.gca.2007.09.004

81. Farkaš J, Buhl D, Blenkinsop J, Veizer J. Evolution of the oceanic calcium cycle during the late Mesozoic: Evidence from $\delta^{44/40}Ca$ of marine skeletal carbonates. Earth Planet Sci Lett. 2007;253: 96–111. doi:10.1016/j.epsl.2006.10.015

82. Kitch GD, Jacobson AD, Harper DT, Hurtgen MT, Sageman BB, Zachos JC. Calcium isotope composition of *Morozovella* over the latePaleocene–early Eocene. Geology. 2021 [cited 27 Mar 2021]. doi:10.1130/G48619.1

83. Wang J, Jacobson AD, Sageman BB, Hurtgen MT. Stable Ca and Sr isotopes support volcanically triggered biocalcification crisis during Oceanic Anoxic Event 1a. Geology. 2020 [cited 27 Mar 2021]. doi:10.1130/G47945.1

84. DePaolo DJ. Surface kinetic model for isotopic and trace element fractionation during precipitation of calcite from aqueous solutions. Geochim Cosmochim Acta. 2011;75: 1039–1056. doi:10.1016/j.gca.2010.11.020

85. Mills JV, DePaolo DJ, Lammers LN. The influence of $Ca:CO_3$ stoichiometry on Ca isotope fractionation: Implications for process-based models of calcite growth. Geochim Cosmochim Acta. 2021;298: 87–111. doi:10.1016/j.gca.2021.01.016

86. Carré M, Bentaleb I, Bruguier O, Ordinola E, Barrett NT, Fontugne M. Calcification rate influence on trace element concentrations in aragonitic bivalve shells: Evidences and mechanisms. Geochim Cosmochim Acta. 2006;70: 4906–4920. doi:10.1016/j.gca.2006.07.019

87. Wu HC, Dissard D, Douville E, Blamart D, Bordier L, Tribollet A, et al. Surface ocean pH variations since 1689 CE and recent ocean acidification in the tropical South Pacific. Nat Commun. 2018;9: 2543. doi:10.1038/s41467-018-04922-1

88. Chen C-TA, Lui H-K, Hsieh C-H, Yanagi T, Kosugi N, Ishii M, et al. Deep oceans may acidify faster than anticipated due to global warming. Nat Clim Change. 2017;7: 890. doi:10.1038/s41558-017-0003-y

89. Qi D, Chen L, Chen B, Gao Z, Zhong W, Feely RA, et al. Increase in acidifying water in the western Arctic Ocean. Nat Clim Change. 2017;7: 195–199. doi:10.1038/nclimate3228

90. McConnaughey TA, Burdett J, Whelan JF, Paull CK. Carbon isotopes in biological carbonates: Respiration and photosynthesis. Geochim Cosmochim Acta. 1997;61: 611–622. doi:10.1016/S0016-7037(96)00361-4

91. Zhao L, Yang F, Milano S, Han T, Walliser EO, Schöne BR. Transgenerational acclimation to seawater acidification in the Manila clam Ruditapes philippinarum: Preferential uptake of metabolic carbon. Sci Total Environ. 2018;627: 95–103. doi:10.1016/j.scitotenv.2018.01.225

92. Chung M-T, Trueman CN, Godiksen JA, Holmstrup ME, Grønkjær P. Field metabolic rates of teleost fishes are recorded in otolith carbonate. Commun Biol. 2019;2: 24. doi:10.1038/s42003-018-0266-5

93. Chung M-T, Trueman CN, Godiksen JA, Grønkjær P. Otolith $\delta^{13}C$ values as a metabolic proxy: approaches and mechanical underpinnings. Mar Freshw Res. 2019 [cited 23 Oct 2019]. doi:10.1071/MF18317

94. Romanek CS, Grossman EL, Morse JW. Carbon isotopic fractionation in synthetic aragonite and calcite: Effects of temperature and precipitation rate. Geochim Cosmochim Acta. 1992;56: 419–430. doi:10.1016/0016-7037(92)90142-6

95. Eide M, Olsen A, Ninnemann US, Johannessen T. A global ocean climatology of preindustrial and modern ocean δ^{13} C: Climatology of Preindustrial δ^{13} C. Glob Biogeochem Cycles. 2017;31: 515–534. doi:10.1002/2016GB005473

96. Eide M, Olsen A, Ninnemann US, Eldevik T. A global estimate of the full oceanic 13C Suess effect since the preindustrial. Glob Biogeochem Cycles. 2017;31: 492–514. doi:10.1002/2016GB005472

97. Auclair A-C, Lecuyer C, Bucher H, Sheppard SMF. Carbon and oxygen isotope composition of *Nautilus macromphalus*: a record of thermocline waters off New Caledonia. Chem Geol. 2004;207: 91–100. doi:10.1016/j.chemgeo.2004.02.006

98. Linzmeier BJ. Refining the interpretation of oxygen isotope variability in free-swimming organisms. Swiss J Palaeontol. 2019;138: 109–121. doi:10.1007/s13358-019-00187-3

99. Beirne EC, Wanamaker AD, Feindel SC. Experimental validation of environmental controls on the $\delta^{13}C$ of *Arctica islandica* (ocean quahog) shell carbonate. Geochim Cosmochim Acta. 2012;84: 395–409. doi:10.1016/j.gca.2012.01.021

100. Duwig C, Becquer T, Clothier BE, Vauclin M. Nitrate leaching through oxisols of the Loyalty Islands (New Caledonia) under intensified agricultural practices. Geoderma. 1998;84: 29–43. doi:10.1016/S0016-7061(97)00119-5

101. Gaillard C, Manner HI. Yam Cultivation on the East Coast of New Caledonia: adaptation of agriculture to social and economic changes. Aust Geogr. 2010;41: 485–505. doi:10.1080/00049182.2010.519697

102. Bouchet P, Jaffre T, Veillon J-M. Plant extinction in New Caledonia: protection of sclerophyll forests urgently needed. Biodivers Conserv. 1995;4: 415–428. doi:10.1007/BF00058425

103. Orr JC, Fabry VJ, Aumont O, Bopp L, Doney SC, Feely RA, et al. Anthropogenic ocean acidification over the twenty-first century and its impact on calcifying organisms. Nature. 2005;437: 681. doi:10.1038/nature04095

104. Pascal M, Forges BRD, Guyader HL, Simberloff D. Mining and Other Threats to the New Caledonia Biodiversity Hotspot. Conserv Biol. 2008;22: 498–499. doi:https://doi.org/10.1111/j.1523-1739.2008.00889.x

105. Clark MS, Peck LS, Arivalagan J, Backeljau T, Berland S, Cardoso JCR, et al. Deciphering mollusc shell production: the roles of genetic mechanisms through to ecology, aquaculture and biomimetics. Biol Rev. 2020;95: 1812–1837. doi:https://doi.org/10.1111/brv.12640

106. Hahn S, Rodolfo-Metalpa R, Griesshaber E, Schmahl WW, Buhl D, Hall-Spencer JM, et al. Marine bivalve shell geochemistry and ultrastructure from modern low pH environments: Environmental effect versus experimental bias. 2012 [cited 13 Sep 2018]. doi:10.5194/bg-9-1897-2012

107. Beddows PA, Mallon EK. Cave Pearl Data Logger: A Flexible Arduino-Based Logging Platform for Long-Term Monitoring in Harsh Environments. Sensors. 2018;18: 530. doi:10.3390/s18020530

108. Ullmann CV, Korte C. Diagenetic alteration in low-Mg calcite from macrofossils: a review. Geol Q. 2015;59: 3–20. doi:10.7306/gq.1217

109. Witts JD, Newton RJ, Mills BJW, Wignall PB, Bottrell SH, Hall JLO, et al. The impact of the Cretaceous–Paleogene (K–Pg) mass extinction event on the global sulfur cycle: Evidence from Seymour Island, Antarctica. Geochim Cosmochim Acta. 2018;230: 17–45. doi:10.1016/j.gca.2018.02.037

110. Benito MI, Reolid M, Viedma C. On the microstructure, growth pattern and original porosity of belemnite rostra: insights from calcitic Jurassic belemnites. J Iber Geol. 2016;42: 201–226. doi:10.5209/revBJIGE.2016.v42.n2.53232

111. Hoffmann R, Richter DK, Neuser RD, Jöns N, Linzmeier BJ, Lemanis RE, et al. Evidence for a composite organic–inorganic fabric of belemnite rostra: Implications for palaeoceanography and palaeoecology. Sediment Geol. 2016;341: 203–215. doi:10.1016/j.sedgeo.2016.06.001

Supporting information

S1 File. Spreadsheet with all analytical data for all samples. Each tab contains one group of analyses.

Fig1

Click here to access/download;Figure;Fig1.tif

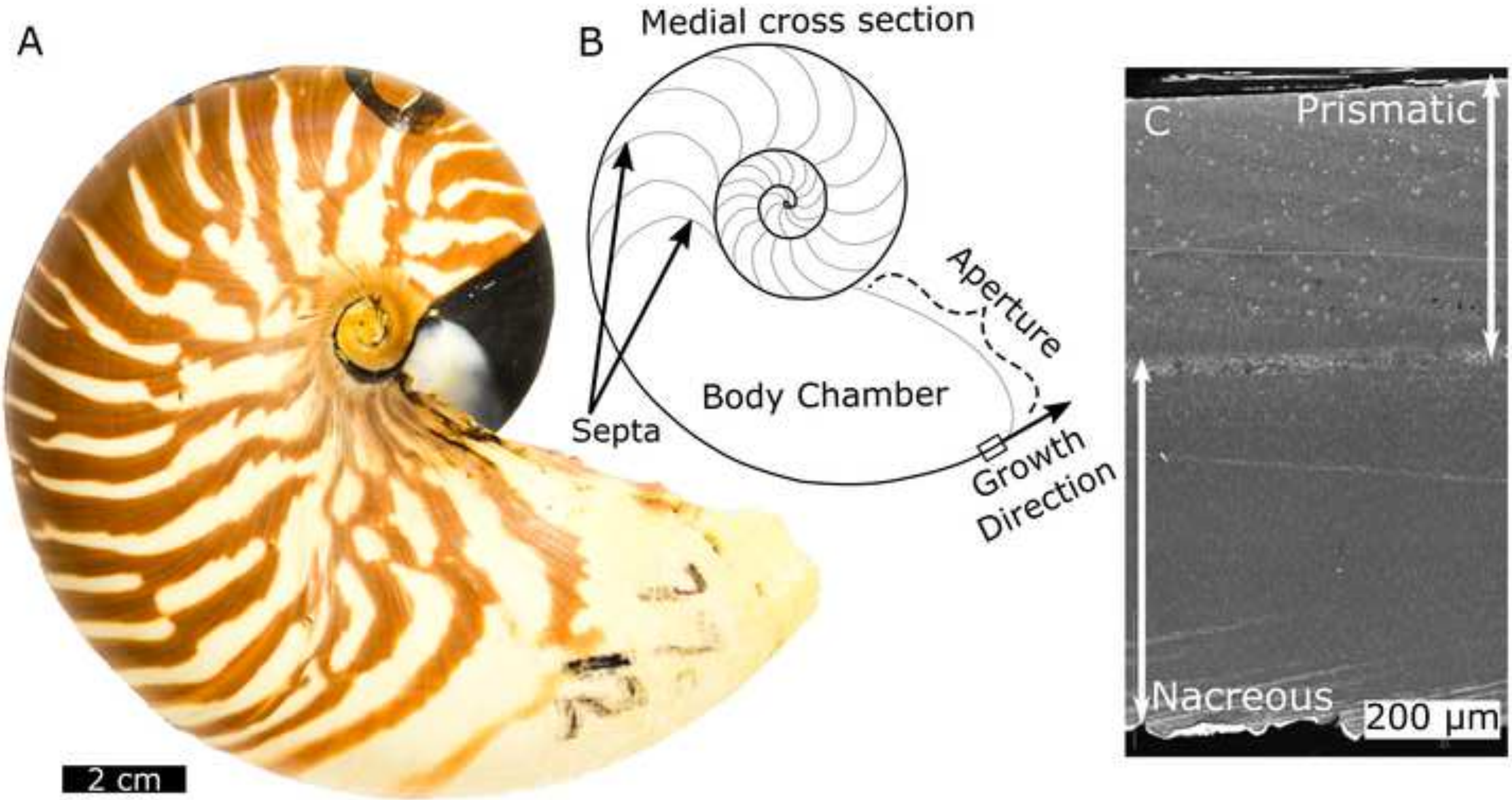

Fig2

Click here to access/download;Figure;Fig2.tif

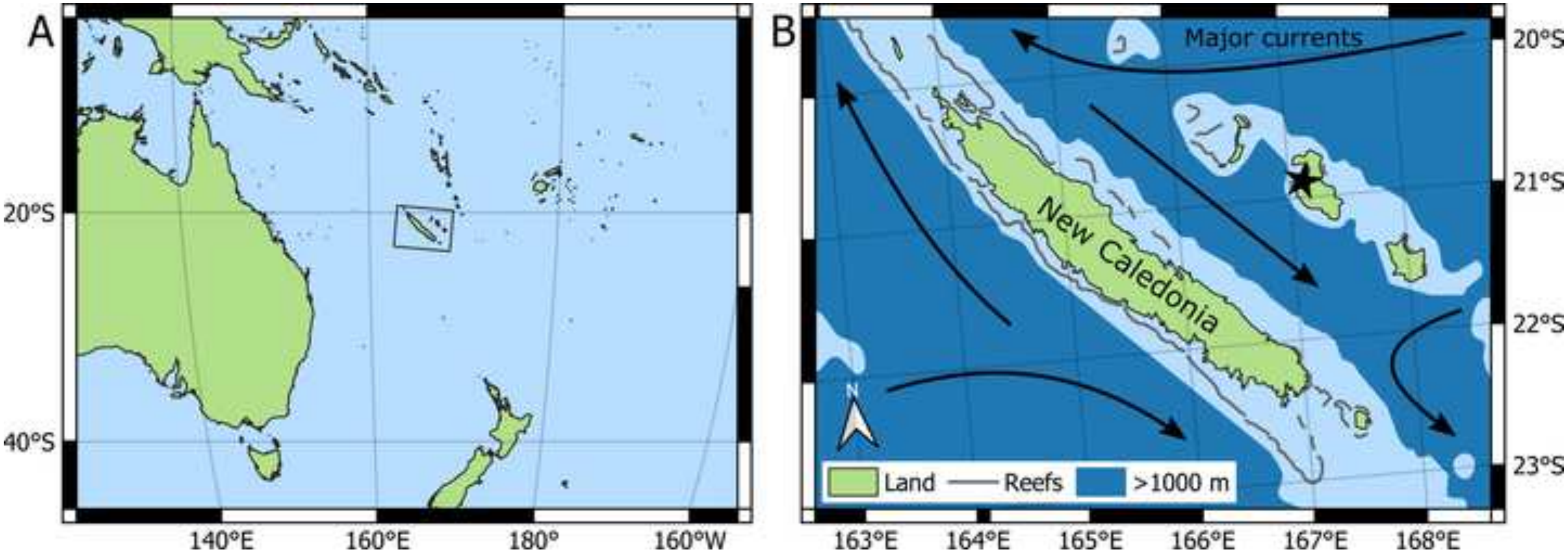

Fig3 Click here to access/download;Figure;Fig3.tif

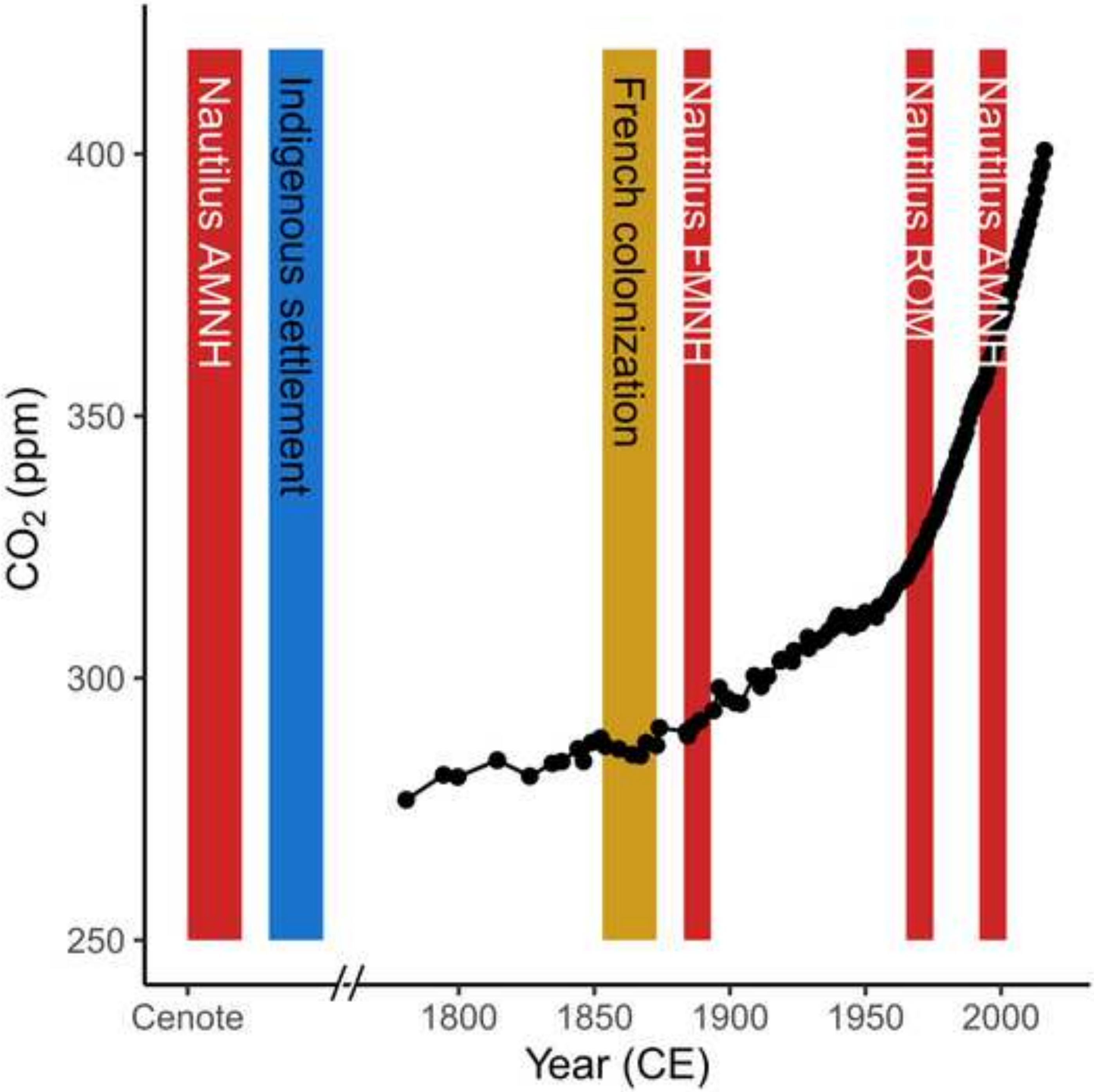

Click here to access/download;Figure;Fig4.tif

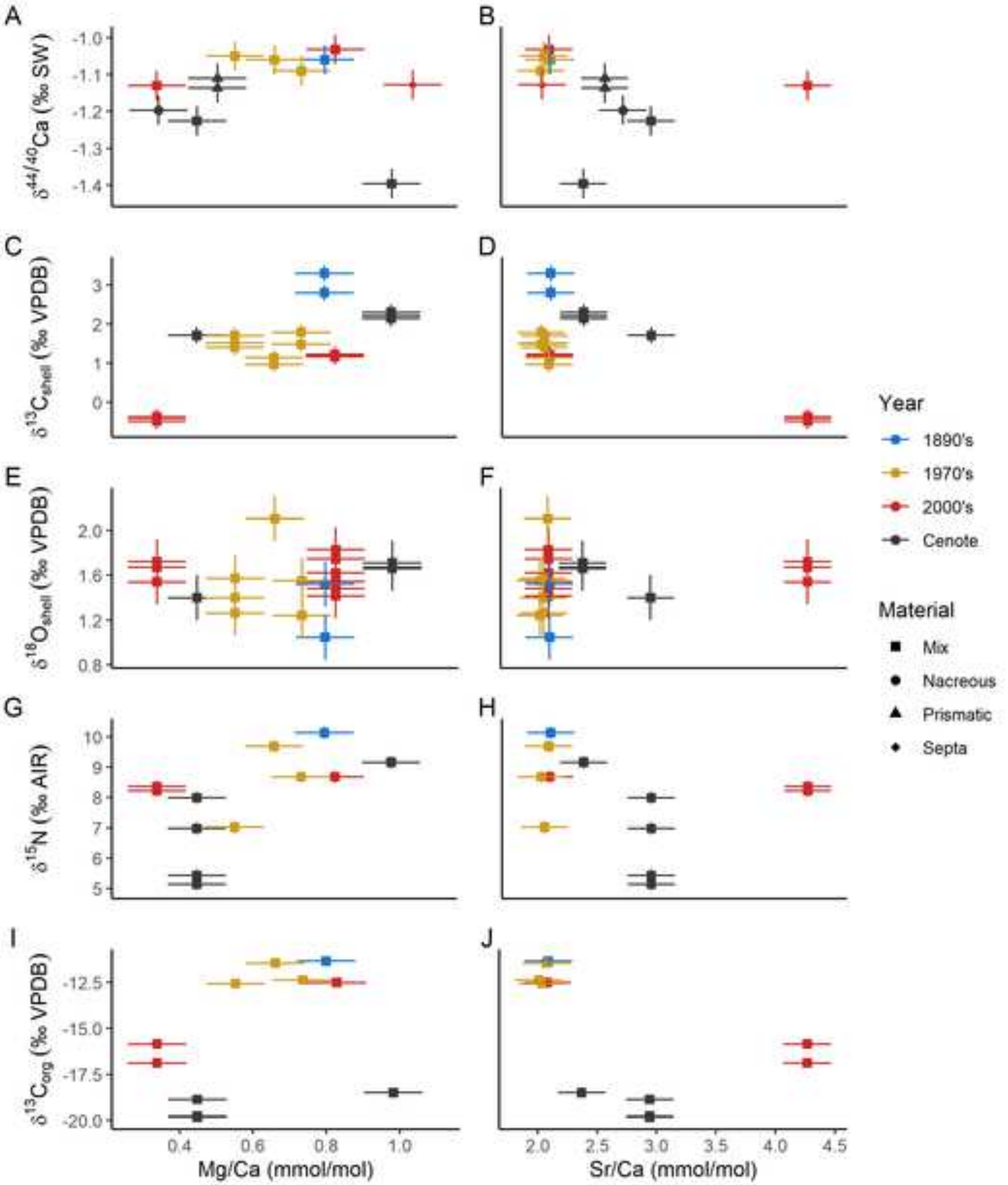

 Click here to access/download;Figure;Fig5.tif

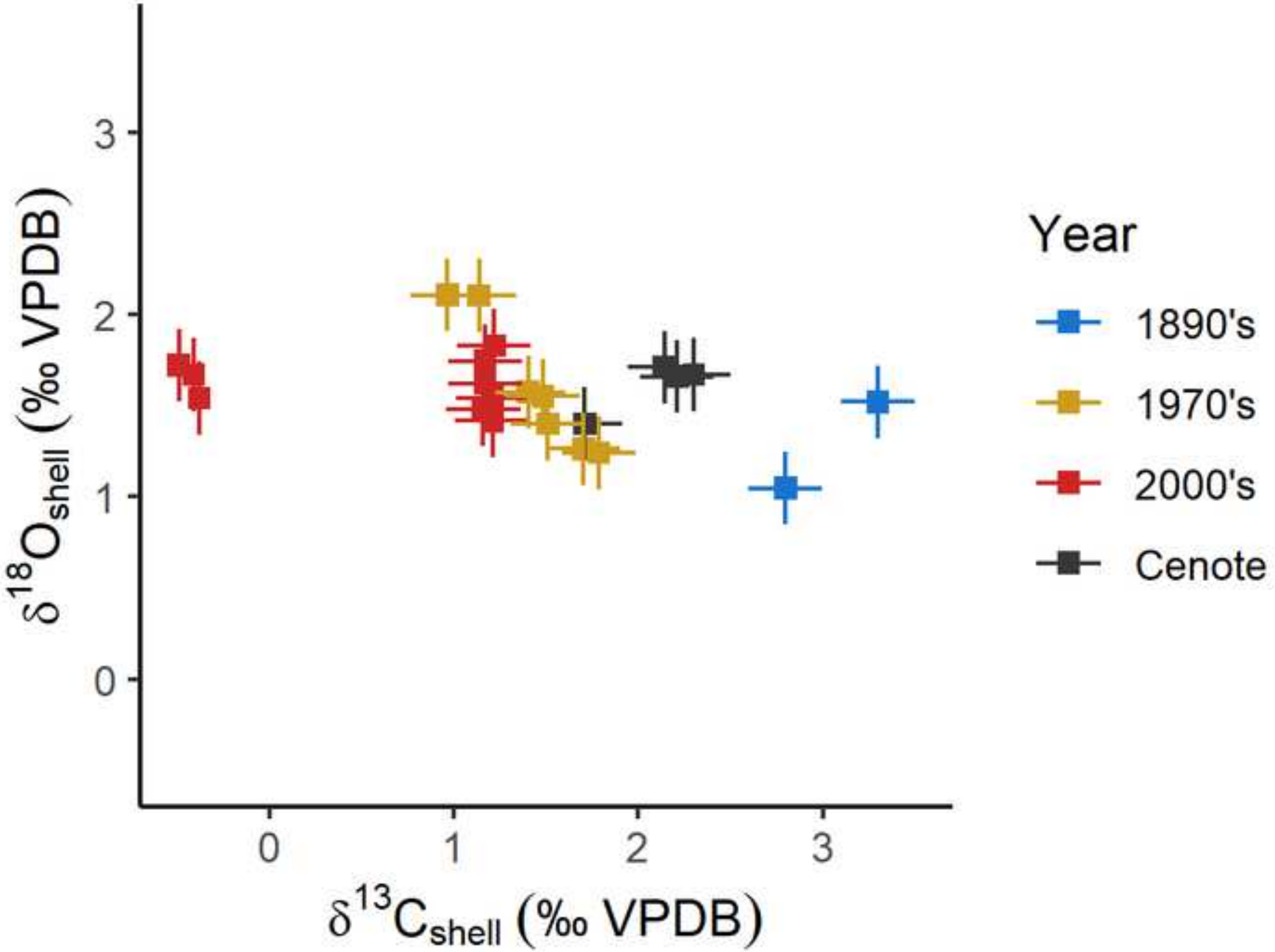

Fig6 Click here to access/download;Figure;Fig6.tif

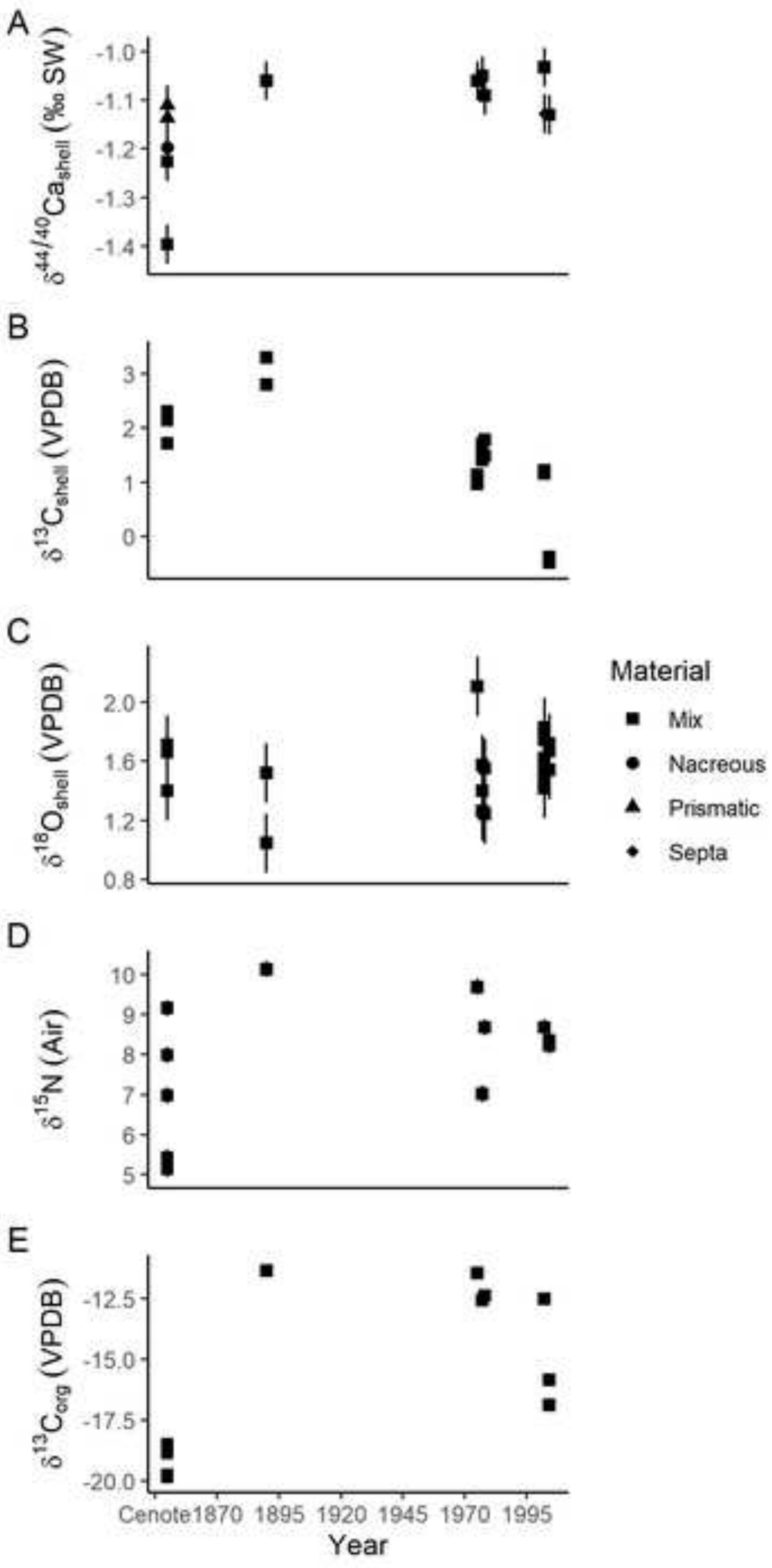

Fig7

Click here to access/download;Figure;Fig7.tif

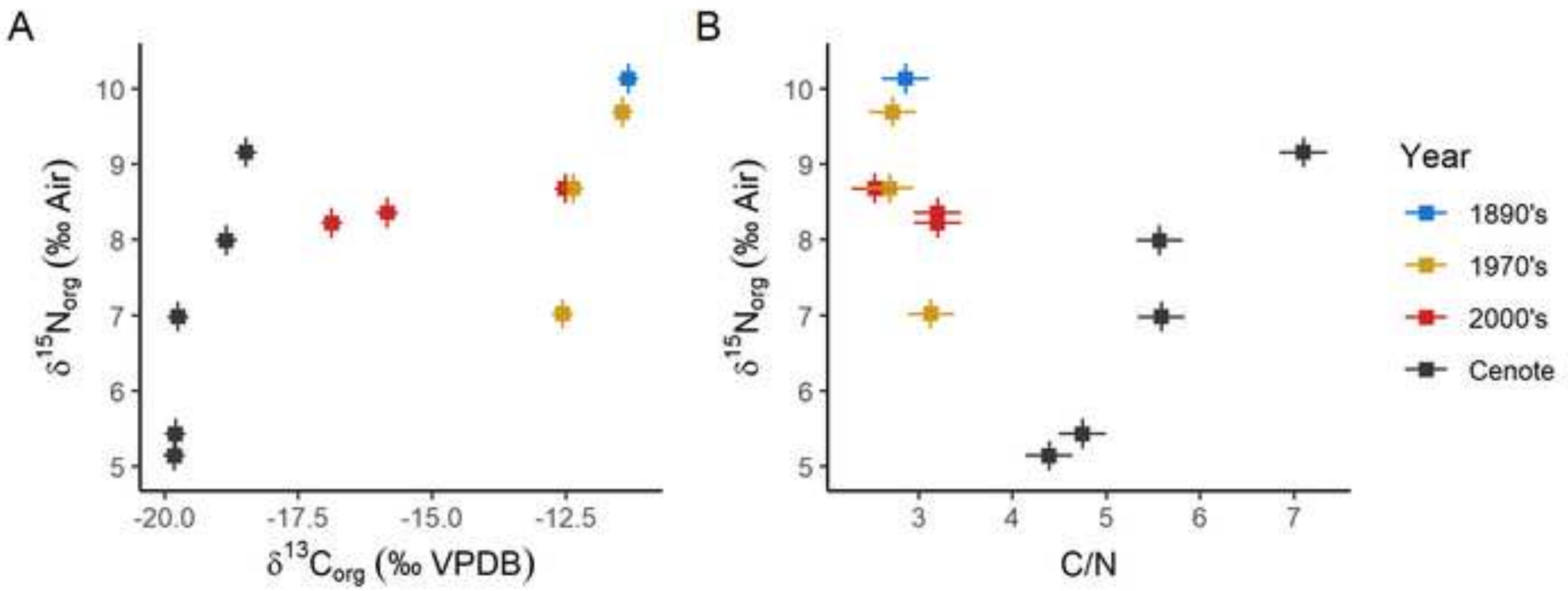

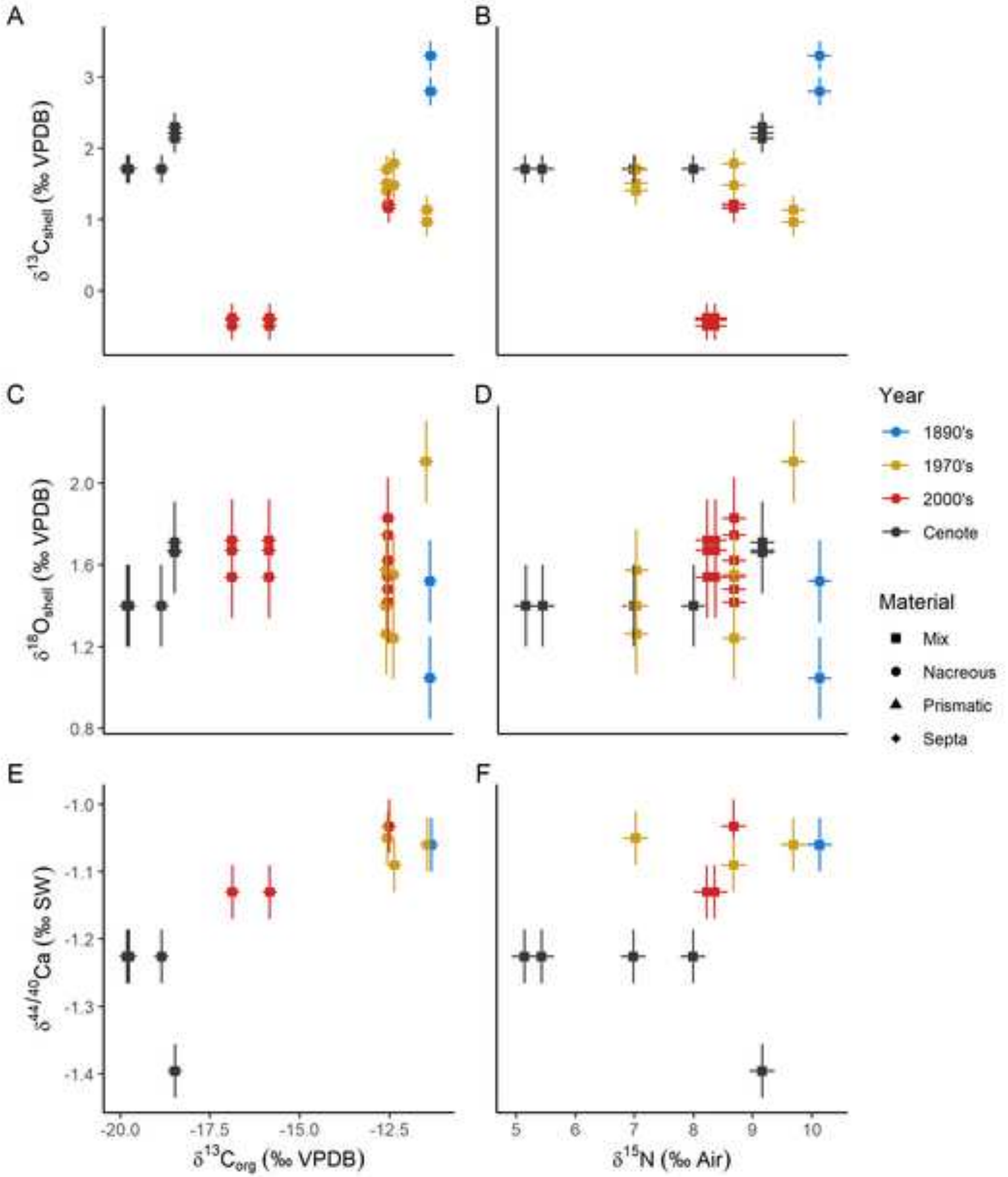
A
B
C
D
E
F
δ13Cshell (‰ VPDB)
δ18Oshell (‰ VPDB)
δ44/40Ca (‰ SW)
δ13Corg (‰ VPDB)
δ15N (‰ Air)
3
2
1
0
2.0
1.6
1.2
0.8
-1.0
-1.1
-1.2
-1.3
-1.4
-20.0
-17.5
-15.0
-12.5
5
6
7
8
9
10
Year
1890's
1970's
2000's
Cenote
Material
Mix
Nacreous
Prismatic
Septa

Fig9

Click here to access/download;Figure;Fig9.tif

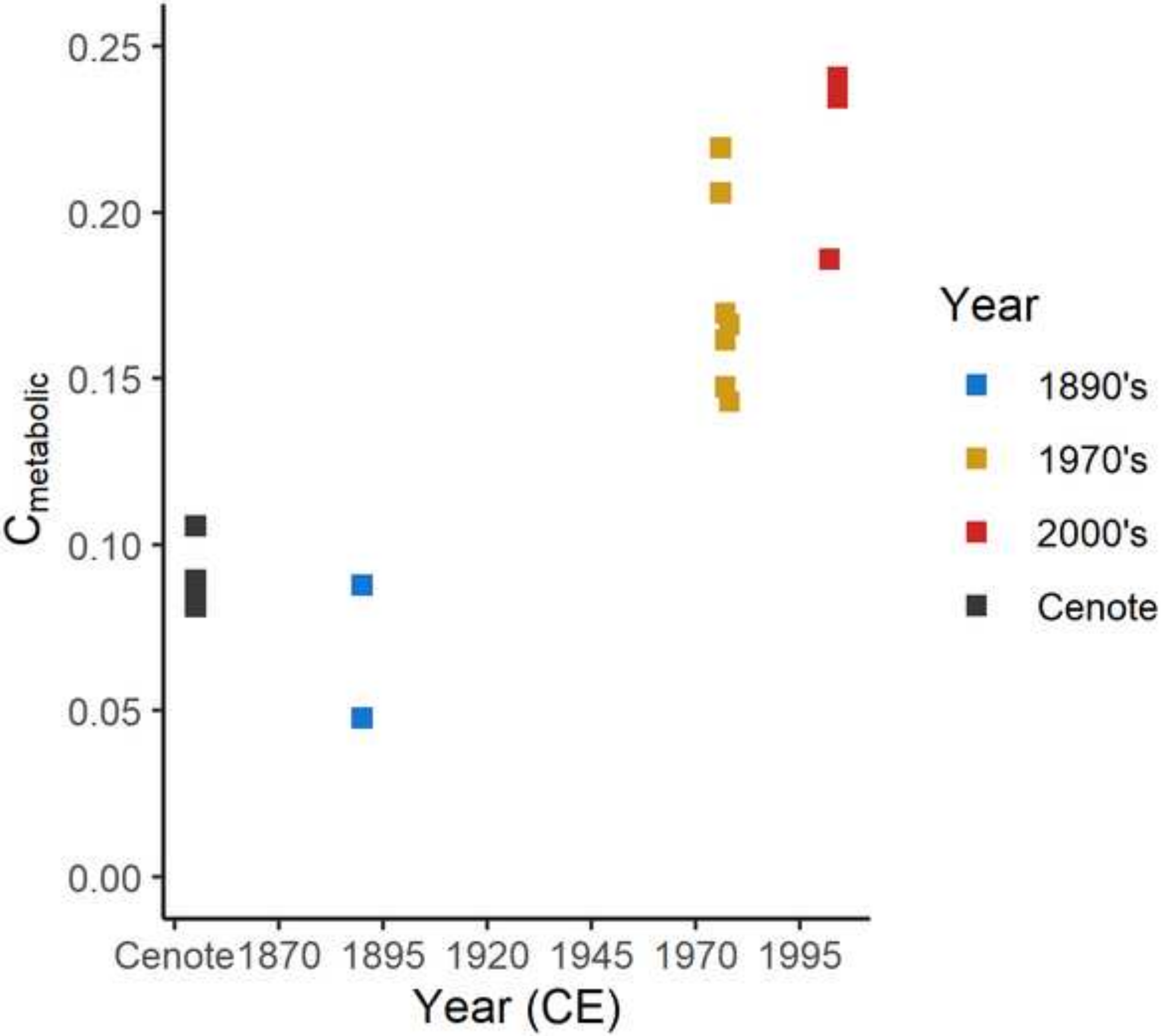

Supporting Data Table SI1

Click here to access/download
Supporting Information
SI_DataTable.xlsx

Revised Manuscript with Track Changes

Preprint for submission to: PLoS One

Isotope systematics of subfossil, historical, and modern *Nautilus macromphalus* from New Caledonia

Benjamin J. Linzmeier[1*]~~,~~, Andrew D. Jacobson[2], Bradley B. Sageman[2], Matthew T. Hurtgen[2], Meagan E. Ankney[3]~~Ankney[2]~~, Andrew Masterson[2], and Neil H. Landman[4]~~Landman[3]~~

[1] Department of Earth Sciences, University of South Alabama, Mobile, Alabama, United States of America~~USA~~

[2] Department of Earth and Planetary Sciences, Northwestern University, Evanston, Illinois, United States of America~~USA~~

[3] Wisconsin State Laboratory of Hygiene, University of Wisconsin – Madison, Madison, Wisconsin, United States of America

[4] Division of Paleontology~~Paleobiology~~, American Museum of Natural History, New York, New York, United States of America~~USA~~

* Corresponding author

Email:

Style Definition: Bibliography: Indent: Left: 0", Hanging: 0.5", Space After: 0 pt, Tab stops: Not at 0.43"

Abstract

Formatted: Font: 18 pt

Cephalopod carbonate geochemistry underpins studies ranging from Phanerozoic, global-scale change to outcrop-scale paleoecological reconstructions. Interpreting these data hinges on assumed similarity to model organisms, such as *Nautilus*, and generalization from other molluscan biomineralization processes. Aquarium rearing and capture of wild *Nautilus* suggest shell carbonate precipitates quickly (35 µm/day) in oxygen isotope equilibrium with seawater. Other components of *Nautilus* shell chemistry are less well-studied but have potential to serve as proxies for paleobiology and paleoceanography.

To calibrate the geochemical response of cephalopod $\delta^{15}N_{org}$, $\delta^{13}C_{org}$, $\delta^{13}C_{carb}$, $\delta^{18}O_{carb}$, and $\delta^{44/40}Ca_{carb}$ to modern anthropogenic environmental change, we analyzed modern, historical, and subfossil *Nautilus macromphalus* from New Caledonia. Samples span initial human habitation, colonialization, and industrial pCO_2 increase. This sampling strategy is advantageous because it

avoids the shock response that can affect geochemical change in aquarium experiments. Given the range of living depths and more complex ecology of *Nautilus*, however, some anthropogenic signals, such as ocean acidification, may not have propagated to their living depths.

Our data suggest some environmental changes are more easily preserved than others given variability in cephalopod average living depth. Calculation of the percent respired carbon incorporated into the shell using $\delta^{13}C_{org}$, $\delta^{13}C_{carb}$, and Suess-effect corrected $\delta^{13}C_{DIC}$ suggests an increase in the last 130 years that may have been caused by increasing carbon dioxide concentration or decreasing oxygen concentration at the depths these individuals inhabited. This pattern is consistent with increasing atmospheric CO_2 and/or eutrophication offshore of New Caledonia. We find that $\delta^{44/40}Ca$ remains stable across the last 130 years. The subfossil shell from a cenote may exhibit early $\delta^{44/40}Ca$ diagenesis. Questions remain about the proportion of dietary vs ambient seawater calcium incorporation into the *Nautilus* shell. Values of $\delta^{15}N$ do not indicate trophic level change in the last 130 years, and the subfossil shell may show diagenetic alteration of $\delta^{15}N$ toward lower values. Future work using historical collections of *Sepia* and *Spirula* may provide additional calibration of fossil cephalopod geochemistry.

~~1.~~ Introduction

Formatted: Font: 18 pt

Formatted: Normal, No bullets or numbering

The geochemistry of cephalopod mollusks has been used to quantify paleoclimate (Price et al., 2013; Urey et al., 1951) and investigate paleobiology (Ellis and Tobin, 2019; Ferguson et al., 2019; Landman et al., 2018; Linzmeier et al., 2018; Lukeneder et al., 2010; Moriya et al., 2003; Sessa et al., 2015) throughout their long fossil record (Kröger et al., 2011). Modern *Nautilus* is a model organism for the isotope geochemistry of extinct cephalopods, including the ammonoids and nautiloids, because it is the only remaining externally shelled cephalopod (Fig 1). *Nautilus*

are forereef scavengers that cross hundreds of meters of water depth within individual days while continuously growing shell .~~. 1).~~ Extensive work has been done to quantify the systematics of light stable isotopes~~isotope systematics of light stable isotopes~~ ($\delta^{13}C$ and $\delta^{18}O$) in *Nautilus* shell carbonate. Aquarium rearing experiments suggest *Nautilus* shells precipitate in $\delta^{18}O$ equilibrium with seawater (Landman et al., 1994; Linzmeier et al., 2016)~~.~~. Carbon isotope values~~ratio data~~ ($\delta^{13}C_{shell}$) suggest both metabolic and dissolved inorganic carbon (DIC) sources (Landman et al., 1994) similar to that for other mollusks (McConnaughey and Gillikin, 2008)~~.~~. Analyses of $\delta^{15}N$ from shell~~intercrystalline~~ organic matter, also known as conchiolin, show trophic change associated with hatching and maturity (Kashiyama et al., 2010)~~.~~. The carbon isotope value~~ratio~~ of conchiolin, like organic matter in other mollusk shells, likely reflects the $\delta^{13}C$ of respired carbon (Crocker et al., 1985; Gillikin et al., 2007; McConnaughey and Gillikin, 2008)~~.~~.

Fig 1. Overview of *Nautilus m.* shell (ROM 48997) with annotation of major shell structures, growth direction, and shell layers. *Nautilus* shells grow in a spiral and septa are secreted to allow for the maintenance of neutral buoyance during growth through the removal of liquid from the chambers . Shells comprise two major structural forms of aragonite, the inner nacreous layer and outer spherulitic prismatic layer .

Other isotope systems, particularly metal isotopes, have received less investigation. The calcium isotope value~~ratio~~ ($\delta^{44/40}Ca$) of *Nautilus* shell has only recently been measured from a handful of modern shells (Gussone and Heuser, 2016, and references therein) and may reflect changing *Nautilus* fractionation factor ($\Delta^{44/40}Ca_{shell-SW}$, (Linzmeier et al., 2020)~~,~~, changing $\delta^{44/40}Ca$ value of seawater (Holmden et al., 2012)~~,~~, or dietary change (Gussone and Heuser, 2016; Hoffmann et al., 2021)~~.~~.

Proxies respond to anthropogenic forcingsAnthropogenic changes have impacted ocean chemistry and ecosystems through a variety of environmental change.pathways. Some of these shiftschanges are particularly dramatic and likely to impact *Nautilus*, including the rise of coastal ‘dead zones’ (Fennel and Testa, 2019),, overfishing (Barord et al., 2014),, and acidification (Doney et al., 2009).. For most organisms, these environmental changes are unfolding on a generational timescale, so phenotypic plasticity and ecosystem feedbacks may allow for faster adaptation, potentially mitigating the worst possible effects (Kroeker et al., 2017; Schunter et al., 2018; Telesca et al., 2021; Urban et al., 2016);; however, it is difficult to assess deep-time analogs for change on timescales of 100’s to 10,000’s of years due to the time-averaged and punctuated nature of the fossil record (Kidwell, 2002; Kowalewski et al., 2018).. Careful, high precision analyses of proxies across modern to historical samples provide an opportunity to calibrate the geochemical changes expected in fossil archives and provide insight into variations expected to be preserved within fossil assemblages during abrupt climate events.

Here, we test for hypothesized anthropogenic forcings (e.g. temperature change, ocean acidification, trophic change) and diagenetic vulnerability present in a suite of isotope analyses (Sr/Ca, Mg/Ca, $\delta^{13}C_{org}$, $\delta^{15}N_{org}$, $\delta^{13}C_{carb}$, $\delta^{18}O_{carb}$, and $\delta^{44/40}Ca_{carb}$) of *Nautilus macromphalus* from New Caledonia (Fig.. 2). Samples range in age from modern to subfossil and span indigenous settlement (Stevenson et al., 2001),, European colonization, and industrial ocean acidification and warming (Fig.. 3). We hypothesize abrupt changes in ecologically sensitive proxies (e.g. $\delta^{18}O_{carb}$, $\delta^{15}N_{org}$) are stepwise dueSamples show analytically resolvable differences that may reflect trophic change, diagenetic alteration, or response to ecological restructuring, while gradual changes are forced due to continuous industrial environmental effects.changing ocean chemistry through this interval. These data highlight the utility of museum collections and

the subfossil record for better quantifying past environmental change to predict biological resiliency in the face of anthropogenic pressures.

Fig 2. Map showing the sample collection location and major currents surrounding New Caledonia. Data are from www.naturalearthdata.com. A) World map showing the location of New Caledonia in the Southwestern Pacific Ocean. B) Local map showing New Caledonia and the surrounding islands with a star indicating the location of the cenote. Water shallower than 1000 m is in light blue and approximates the habitat of Nautilus m. around New Caledonia. All cenote specimens are from the star location of Lifou, Loyalty Islands . All other specimens were collected near the large island and are recent museum specimens. Reliable accounts of the live collection of Nautilus m. are mostly known from areas close to New Caledonia, and the species is thought to have a geographic range restricted to that region . Local currents are overlain for reference .

Fig 3. Timeline for sample ages, pCO_2, and human settlement of New Caledonia. Purple vertical bars represent the age of specimens used in this study with the museum repositories noted. Yellow vertical bar represents indigenous settlement as demonstrated by landscape change observed in lacustrine sediments ~3000 BCE . French invasion occurred in the mid-1800's. The comparative record of pCO_2 change showing industrial increases is from Law Dome, East Antarctica .

~~2.~~ Materials and Methods

~~2.1~~ Ethics statement

Formatted: Indent: First line: 0"

Formatted: Font: Bold, Not Italic

Formatted: Font: Not Italic

Formatted: Font: Bold, Not Italic

Formatted: Font: Not Italic

Formatted: Font: Not Italic

Formatted: Font: 18 pt

Formatted: Normal, No bullets or numbering

Formatted: Font: 16 pt

All specimens of *Nautilus macromphalus* (Mollusca: Cephalopoda) used in this study were collected for morphological study of *Nautilus* and deposited at various museums (American Museum of Natural History, Field Museum of Natural History, Royal Ontario Museum). The methods of capture and ~~euthanasia~~ sacrifice of Nautilus before the 2000's specimens are unknown, but all specimens were collected before local protected status was granted in 2008. The two individuals collected in 2002 and 2004 were captured in a baited trap by Dr. Royal H. Mapes, sacrificed~~euthanized~~sacrificed, and kept for a morphological study~~ of *Nautilus* *macromphalus*~~.

~~2.2~~ *Nautilus macromphalus* samples

Formatted: Font: 16 pt

Formatted: Normal, No bullets or numbering

Nautilus *m.*~~*macromphalus*~~ is endemic to New Caledonia and the Loyalty Islands and can be distinguished~~distinguishable~~ by the prominent, open umbilicus with inward sloping walls (Saunders, 2010)(,~~;~~ Fig~~.~~ 1). Individual *Nautilus* likely grow to maturity over 2.5 to 6 years, although considerable uncertainty remains in their total lifespan (Landman and Cochran, 2010)~~.~~. The oldest samples used in this study are AMNH 112600~~11260~~ and AMNH 112595. These samples were recovered from a cenote~~Cenote~~ on Lifou (Fig~~.~~ 2) and date to 6455 ±30 (AMNH 112600) and 6895 ±30 (AMNH 112595)~~~6800~~ years before present using radiocarbon (Landman et al., 2014)~~.~~. Multiple fragments from near the aperture of this shell were powdered to provide adequate material for analyses. The second oldest sample is from the Field Museum of Natural History in Chicago (FM 41) and was part of the original collection purchased from Wards Scientific to start the collection after the World's~~World's~~ Columbian Exposition in 1893. A single 2 cm^2 chip from near the aperture was removed for analysis. Three samples from the Royal Ontario Museum (ROM 60177, 48997, and 49000) were collected in 1975, 1977, and 1978 respectively. Small chips of approximately 2 cm^2 were removed from the aperture. The

most recent samples, housed at the American Museum of Natural History (AMNH 105621, AMNH 123397), were collected in 2002 and 2004, respectively. One sample, AMNH 105621, was previously studied for $\delta^{18}O_{carb}$ variability by secondary ion mass spectrometry (Linzmeier et al., 2016)~~.~~. For this sample, powder was drilled from a chip that was embedded in epoxy.

~~2.3~~ Organic matter $\delta^{13}C$ and $\delta^{15}N$

Formatted: Font: 16 pt

Formatted: Normal, No bullets or numbering

Processing of carbonate to extract organic matter was done in the Sedimentary Geochemistry Laboratory at Northwestern University. Powdered shell was reacted with room temperature 1 N HCl overnight. Smaller amounts of recent shells with abundant organic matter were reacted with ~~less~~ smaller volumes of HCl necessary for complete dissolution and then dried down to isolate the organic matter. The subfossil shells from the cenote required ~1-2 g of shell for the analyses presented here.~~.~~ Organic matter was collected from solution using ashed glass filters. Either organic~~Organic~~ matter or organic matter plus filters were~~was then~~ loaded into tin capsules and combusted at 980 ° C on a Costech 4010 Elemental Analyzer, coupled to a ThermoFinnigan Delta V Plus mass spectrometer via a ConFlo IV interface in the Stable Isotope Laboratory at Northwestern University. Long term precision of $\delta^{13}C$ and $\delta^{15}N$ analyses (±0.4~~2~~‰ and ±0.6‰, $2\sigma_{SD}$~~3‰, $1\sigma_{SD}$~~) was assessed by comparison with isotope standards IU acetanilide ($\delta^{13}C$ = -29.52±0.02‰, $\delta^{15}N$ = +1.18‰) and IU urea ($\delta^{13}C$ = -8.02±0.05‰, $\delta^{15}N$ = +20.17±0.06‰) supplied by Indiana University (Schimmelmann et al., 2009). This method of measurement does not distinguish between carbonate-bound organic matter (intracrystalline) and non-carbonate bound organic matter (intercrystalline) but produces average $\delta^{13}C$ and $\delta^{15}N$ values for all organic matter isolated from the *Nautilus* shell.~~.~~

~~2.4~~ Carbonate $\delta^{13}C$ and $\delta^{18}O$

Formatted: Font: 16 pt

Formatted: Normal, No bullets or numbering

Analyses of carbonate powder were conducted~~done~~ in the Stable Isotope Laboratory at Northwestern University using a Thermo Gasbench II coupled with continuous flow of He carrier gas to a Thermo Delta V IRMS. Values were standardized to the Vienna Pee Dee Belemnite (VPDB) scale using NBS-18 (National Bureau of Standards, $\delta^{18}O$ = -23.2 ‰, $\delta^{13}C$ = -5.01 ‰, VPDB) and an in-house carbonate standard, Carrera~~Carraera~~ Lago Marble (CLM $\delta^{18}O$ = -3.66 ‰,~~,~~ $\delta^{13}C$ = 2.31 ‰, VPDB). The CLM standard has been calibrated alongside NBS-19 and IAEA-603. A two-point calibration using CLM and NBS-18 was employed for the analyses presented here. Analytical uncertainties ($2\sigma_{SD}$) for these analyses are ±0.2‰ for $\delta^{18}O_{carb}$ and ±0.1‰ for $\delta^{13}C_{carb}$.

~~2.5~~ Elemental analysis

Formatted: Font: 16 pt

Formatted: Normal, No bullets or numbering

Approximately 50 mg of powdered shell was placed in acid-washed HDPE test tubes and dissolved in ~10 mL of 5 wt% HNO_3. The mixtures were agitated on a rocker table for ~24 hours, centrifuged, and then filtered through 0.45 μm syringe filters. Solutions were diluted with 5 wt% HNO_3 to ensure elemental concentrations were within calibration ranges of standards (assuming near-to-pure calcium carbonate). Measurements were done using a Thermo Scientific iCAP 6500 ICP-OES using Argon carrier gas and a Cetac U-6000AT+ Ultrasonic Nebulizer in the Aqueous Geochemistry Laboratory at Northwestern University. Repeated measurements of NIST SRM 1643f were used to both monitor instrument stability and confirm data quality for Ca, Mg, Mn, Na, and Sr. Three replicates of 3mL of solution were averaged for standards, blanks, and samples. Instrument drift was monitored using a standard, sample, standard bracketing technique with no more than 15 samples analyzed between standards.~~.~~ Standard measurements suggest an instrumental precision of ±5% (RSD, relative standard deviation) for each element (Ca ~0.5 ppm , Mg ~0.1 ppb, Mn ~0.1 ppb, Na ~6 ppb, and Sr ~5 ppb).

2.6 Carbonate $\delta^{44/40}$Ca

Formatted: Font: 16 pt

Formatted: Normal, No bullets or numbering

Formatted: Font: 16 pt, Not Bold

Formatted: Font: 16 pt

Calcium isotope valuesratio analyses were measuredperformed in the Radiogenic Isotope Laboratory at Northwestern University using an optimized ^{43}Ca-^{42}Ca double spike method implemented on a Thermo-Fisher Triton Multi-Collector Thermal Ionization Mass Spectrometer (TIMS) (Lehn et al., 2013).. Sample solutions containing 50 µg of Ca were weighed into acid-cleaned Teflon vials, spiked, and equilibrated. Solutions were dried down overnight at 90 °C. Residues were then dissolved in 0.5 mL of 1.55N HCl, and Ca was separated from other elements by passing solutions through Teflon columns filled with Bio-Rad AG MP-50 cation exchange resin. Solutions were dried down once more and subsequently treated with two drops of 35 wt% H_2O_2 to oxidize organic compounds and finally two drops of 16N HNO_3 to convert Ca to nitrate form. Samples were then dissolved in 0.4 µL of 8N HNO_3 and split into 4 beads. One bead was loaded onto a single degassed tantalum filament assembly, between ~0.5 mm wide parafilm dams, and subsequently dried at with 3.5 amps after adding 0.5 µL of 10 wt% H_3PO_4.

A stable, 20V ^{40}Ca ion beam was obtained after a 0.5 hr warm-up sequence. Measurement of ^{40}Ca/^{42}Ca, ^{43}Ca/^{42}Ca, and ^{43}Ca/^{44}Ca ratios was accomplished using a three-hop collector cup configuration. Sample (SMP) ^{44}Ca/^{40}Ca ratios are reported in delta notation relative to OSIL seawater (SW), where $\delta^{44/40}Ca_{SMP} = [(^{44}Ca/^{40}Ca)_{SMP} / (^{44}Ca/^{40}Ca)_{SW} - 1] \times 1000$. AnalysesAnalysis of the OSIL SW standard yielded ahad reproducibility of ±0.04‰ ($2\sigma_{SD}$) during the analytical sessions including these samples. Analyses of NIST 915b producedyield an average $\delta^{44/40}$Ca value of -1.14‰ ± 0.07‰ ($2\sigma_{SD}$) during the period duration of the study. All stable isotope and elemental data are available in the Supplemental data file (SI 1).

All stable isotope and elemental data are available in the Supplemental data file (SI 1).

Difference estimates

The difference estimates between smaller subsets of data are based on a Monte Carlo approach. We add a random error term to each analysis assuming the instrumental precision describes two standard deviations of a normal distribution for expected variability around our measured values. We then calculate the difference between groups for this new dataset and repeat this operation 1,000,000 times. Error estimates are reported as the 5th to 95th percentiles of the differences between the groups from the Monte Carlo method.

~~3.~~ Results

Formatted: Font: 18 pt

Formatted: Normal, No bullets or numbering

~~3.1~~ Carbonate trace elements

Formatted: Font: 16 pt

Recent samples show no discernible temporal trends in either Mg/Ca or Sr/Ca ratios and display similar variation to the subfossil specimens (Fig~~.~~ 4). Material from subfossil cenote specimens has higher Sr/Ca (3.0 mmol/mol) and lower Mg/Ca (0.5 mmol/mol) than most historical samples (Fig~~.~~ 4).

Formatted: Font: 14 pt, Bold

Fig 4. Crossplots of Sr/Ca and Mg/Ca ratios vs isotope values measured on shell carbonate. Error bars are 2 standard deviation instrumental precision for isotope values and the 5% RSD propagated to the elemental ratios. A) $\delta^{44/40}$Ca vs Mg/Ca, B) $\delta^{44/40}$Ca vs Sr/Ca, C) $\delta^{13}C_{shell}$ vs Mg/Ca, D) $\delta^{13}C_{shell}$ vs Sr/Ca, E) $\delta^{18}O_{shell}$ vs Mg/Ca, F) $\delta^{18}O_{shell}$ vs Mg/Ca, G) δ^{15}N vs Mg/Ca, H) δ^{15}N vs Sr/Ca, I) $\delta^{13}C_{org}$ vs Mg/Ca, and J) $\delta^{13}C_{org}$ vs Sr/Ca. The cenote and historical samples distinctly differ along Mg/Ca, Sr/Ca, δ^{15}N, $\delta^{13}C_{org}$, and $\delta^{44/40}$Ca axes.

Formatted: Font: Not Italic

~~3.2~~ Carbonate isotopes

The cenote specimens fall within the range of $\delta^{18}O$ and $\delta^{13}C$ of shell carbonate measured for modern specimens (Fig~~.~~ 5). No change in $\delta^{44/40}Ca$ is apparent in these samples less than 120 years old; however, the subfossil cenote specimens are ~~∼~~0.12‰ lower (0.11 to 0.15‰) than modern samples (Fig~~.~~ 6 A). For samples collected in the last 120 years, $\delta^{13}C_{shell}$ decreases by ~~∼~~2.09‰ (2.03 to 2.15‰)~~‰~~ toward the present (Fig~~.~~ 6 B), while $\delta^{18}O_{shell}$ increases by 0.32‰ (0.45 – 0.20‰)~~∼0.5‰~~ (Fig~~.~~ 6 C).

Fig 5. Crossplot of carbon and oxygen isotope values measured on shell carbonate. Error bars are 2 standard deviation instrumental precision.

Fig 6. Stable isotope values through time. Error bars are 2 standard deviation instrumental precision. A) Shell $\delta^{44/40}Ca$ through time. B) The $\delta^{13}C$ of shell carbonate through time. C) The $\delta^{18}O$ of shell carbonate through time. D) The $\delta^{15}N$ of shell organic matter though time. E) The $\delta^{13}C$ of shell organic matter through time.

~~3.3~~ Organic matter isotopes

Samples spanning the last 130 years show no temporal trends in either $\delta^{13}C_{org}$ or $\delta^{15}N_{org}$ (Fig~~.~~ 6 D and E and Fig~~.~~ 7). Subfossil cenote specimens have lower $\delta^{13}C_{org}$ and $\delta^{15}N_{org}$ by 6.06‰ (6.25 to 5.87‰)~~∼7‰~~ and 1.74‰ (2.03 to 1.45‰),~~∼3‰,~~ respectively. Recent samples also consistently have lower C/N (<3) compared to subfossil specimens (>4, Fig~~.~~ 7 B).

Fig 7. Isotope values of organic matter and the C/N ratio of the organic matter. Error bars are 2 standard deviation instrumental precision. A) $\delta^{13}C_{org}$ vs $\delta^{15}N_{org}$. B) C/N vs $\delta^{15}N_{org}$.

Formatted: Font: 16 pt
Formatted: Normal, No bullets or numbering
Formatted: Font: Bold, Not Italic
Formatted: Font: Not Italic
Formatted: Font: 16 pt
Formatted: Normal, No bullets or numbering
Formatted: Font: 14 pt, Bold

~~4.~~ Discussion

~~4.1~~ Diagenesis

Elemental ratios

Elemental ratios of biogenic carbonates are vulnerable to diagenetic alteration. Increases in Sr/Ca from 2 mmol/mol to 5 mmol/mol have been correlated with microstructural diagenetic alteration of fossil cephalopod shell and correspond to change in $^{87}Sr/^{86}Sr$, $\delta^{18}O_{carb}$, and $\delta^{13}C_{carb}$. The cenote samples do not appear to be outliers in either Sr/Ca or Mg/Ca compared to modern samples, however. This suggests that diagenetic alteration of Sr/Ca and Mg/Ca is not detectable in the cenote samples.

~~4.1.1~~ Organic matter isotopes

Although organic matter within mollusk shells has been reported for multiple fossil specimens, pristine preservation is rare (Buchardt and Weiner, 1981; Clark, 1999; Myers et al., 2018; Weiner et al., 1976). During dissolution of these shells for isolation of organic matter, it is apparent that cenote specimens have considerably less organic matter than modern specimens, but the effects on the isotope composition of residual organic matter is important to explore.~~.~~ Degradation of organic matter within shells generally removes more labile organic~~amino acids~~organic matter (e.g. amino acids, glycoproteins, sugars) and preserves refractory organic matter~~recalcitrant ones~~, although the exact effect on $\delta^{13}C_{org}$ and $\delta^{15}N_{org}$ values is unknown. Two sources of organic matter in the shell, carbonate-bound organic matter (intracrystalline) and non-carbonate bound organic matter (intercrystalline), likely behave differently during diagenesis due to the differing volumes and connectedness of the organic matter for microbial degradation.

Formatted: Font: 18 pt

Formatted: Normal, No bullets or numbering

Formatted: Font: 16 pt

Formatted: Normal, No bullets or numbering

Some authors have noted much lower $\delta^{15}N$ (1-4‰) in significantly~~much~~ older fossil nautiloids (*Cymatoceras sakalavus*, Albian ~113 to 100 Ma) and have suggested these low $\delta^{15}N$ values reflect diagenetic alteration (Kashiyama et al., 2010)~~.~~. Sedimentary organic matter degradation drives $\delta^{15}N$ toward lower values while C/~~:~~N increases (Junium and Arthur, 2007)~~.~~. We see a similar pattern between the recent and subfossil specimens (Fig~~.~~ 7 B) and therefore suggest that the $\delta^{15}N$ of the cenote specimens is altered and does not reflect a primary trophic level signature. The $\delta^{13}C_{org}$ values in these samples are also ~~aberrant~~ lower than~~compared to the~~than the more recent specimens (Fig~~.~~ 7 A). Diagenetic alteration of $\delta^{13}C_{org}$ in mollusks also likely drives isotope values lower due to a loss of the hydrophilic fraction of organic matter and potentially transformation to hydrophobic organic matter. The transformation involves preferentially losing ^{13}C by not reincorporating it in hydrophobic organic matter~~This may be due to different food available in the cenote during the last several months of life, or other unknown oceanographic circulation changes around New Caledonia~~.

Formatted: Font: 12 pt, Not Bold

~~4.1.2~~ Carbonate isotopes

Formatted: Normal, No bullets or numbering

Diagenetic alteration of light stable isotopes is of particular interest for constraining the fidelity of paleoclimatological records (Cochran et al., 2010; Immenhauser et al., 2016; Swart, 2015)~~.~~. The subfossil specimens from the cenote show signs that shell nacre recrystallized (Landman et al., 2014)~~,~~, and higher Sr/Ca in these shells is consistent with addition of either strontianite (Cochran et al., 2010) or authigenic aragonite (Fig~~.~~ 4B). The lack of clear $\delta^{13}C$ or $\delta^{18}O$ outliers is notable and suggests that any recrystallization did not incorporate large volumes of additional diagenetic carbonate. Shell $\delta^{44/40}Ca$ may have been altered toward lower values, as has been observed in Cretaceous mollusks from Antarctica (Linzmeier et al., 2020)~~,~~, where trends toward higher Sr/Ca (5 up to 12 mmol/mol) correlate with lower $\delta^{44/40}Ca$ values (from -

1.5 to -2‰).. This could point to the addition of small domains of authigenic aragonite or strontianite, but the variation observed in the Sr/Ca in the most recent *Nautilus m.* (Fig 4) suggests that the pattern may not be consistent with diagenesis.

If any diagenesisThese apparent alteration effects occurred, it happened at low temperatures (~28 °C), ambient pressures, and within the last 7000 years (Landman et al., 2014) given the location of the cenote and previously published radiocarbon ages.. Carbonate recrystallization/precipitation was likely tied to microbial degradation of organic matter causing variable carbonate saturation state within or near shells (Eyre et al., 2018; Ries et al., 2016)..
Future work contrasting these diagenetic pathways with higher temperature hydrothermal experiments (Milano et al., 2016; Pederson et al., 2019) may help provide a more detailed ‘taphonomic chronometer’ for use in deep time (Tomašových et al., 2017)..

4.2 Isotope and elemental interpretation

Formatted: Font: 16 pt

Formatted: Normal, No bullets or numbering

Formatted: Font: 16 pt

Elemental ratios

The elemental ratios of *Nautilus* shell potentially reflect taxonomic or geographic differences rather than temperatures, as has been observed in bivalve mollusks. We observe a wide range of Sr/Ca and Mg/Ca across these samples with no clear trend towards modern samples. This variation within modern specimens suggests that more work on Mg/Ca and Sr/Ca variation within *Nautilus* is needed to understand the limits of intraspecific variation and potential thresholds for identification of diagenetic alteration.

4.2.1 Organic matter isotopes

Formatted: Normal, No bullets or numbering

Modern and historical shells suggest slight variation in prey preference or availability for the individuals measured (Fig~~.~~ 7 A). In other work on *Nautilus*, $\delta^{15}N_{org}$ changes with hatching and early growth and variation of ~1-2‰ within and between individuals has been observed (Kashiyama et al., 2010)~~.~~. Similar individual – to – individual and within individual variability may be linked to differing prey choice (Newsome et al., 2012, 2009) and is likely to be present in *Nautilus* given neurological complexity (Young, 2010)~~.~~. There are no strong trends through time in the recent $\delta^{13}C_{org}$ or $\delta^{15}N_{org}$ (Fig~~.~~ 6 D and E and Fig~~.~~ 7 A) that would suggest major trophic changes for these *Nautilus m.*~~*macromphalous*~~*macromphalus* in the last ~120 years. Future work using compound specific $\delta^{15}N_{org}$ analysis of amino acids within samples like these could support trophic level stability.

~~4.2.2~~ Calcium isotopes

Formatted: Normal, No bullets or numbering

Previous studies have proposed that~~In vertebrates,~~ $\delta^{44/40}Ca$ can indicate~~has been proposed to be a~~ trophic level for vertebrates~~indicator~~ in modern and ancient terrestrial and marine ecosystems (Martin et al., 2017; Skulan and DePaolo, 1999)~~.~~. Ingestion of hard tissue material and subsequent use of calcium from that source in biomineralization is likely necessary to cause a $\delta^{44/40}Ca$ trophic level effect (Heuser et al., 2011)~~.~~. It is possible that the difference measured between the oldest *Nautilus m.*~~*macromphalus*~~ and the more recent samples (Figs~~.~~ 6 A and~~,~~ 8 F) reflects~~is due to~~ changing composition of scavenged prey, including crustaceans and fish (Saunders and Ward, 2010)~~,~~, perhaps due to ecosystem changes coupled to fishing pressures (Cinner et al., 2020; Guillemot et al., 2009)~~.~~. Shifting dietary composition causing different $\delta^{44/40}Ca$ is an unlikely explanation, however, because no strong correlation with either $\delta^{15}N_{org}$ or $\delta^{13}C_{org}$ is observed, and moreover, recent individuals show measurable differences in both isotope systems indicative of differing diets (Fig~~.~~ 8 F (Newsome et al., 2012))~~.~~. In addition,

comparing $\delta^{44/40}Ca$ to average $\delta^{15}N$ and trophic level across other modern *Nautilus*, *Sepia*, and *Spirula* ~~also~~ suggests a lack of trophic response in cephalopods (Hoffmann et al., 2021).

Fig 8. Crossplots of isotope values measured in shell organic matter vs shell carbonate. Error bars are 2 standard deviation instrumental precision. A) $\delta^{13}C_{org}$ vs $\delta^{13}C_{shell}$, B) $\delta^{15}N$ vs $\delta^{13}C_{shell}$, C) $\delta^{13}C_{org}$ vs $\delta^{18}O_{shell}$, D) $\delta^{15}N$ vs $\delta^{18}O_{shell}$, E) $\delta^{13}C_{org}$ vs $\delta^{44/40}Ca$, and F) $\delta^{15}N$ vs $\delta^{44/40}Ca$.

Formatted: Font: Not Italic

The calcium isotope composition of mollusk material may vary with the calcium isotope composition of seawater ($\delta^{44/40}Ca_{SW}$) or changes in the fractionation between seawater and precipitated shell ($\Delta^{44/40}Ca_{SW\text{-}Shell}$) (Linzmeier et al., 2020). Bivalve mollusks derive ~80% or more of their shell calcium from seawater sources (Bevelander, 1952) and can make use of only water sources for 100% of calcium in shell precipitation (Borght and Puymbroeck, 1966). While shifts in local-scale Ca cycling dynamics are possible (Holmden et al., 2012), changes in the isotopic composition of the global ocean unlikely explain the observed variations, given the long residence time of Ca in seawater. Evidence from the geologic record, however, suggests that $\Delta^{44/40}Ca_{SW\text{-}Shell}$ is sensitive to seawater carbonate state. Culturing experiments show that the pH of extrapallial fluid from *Arctica islandica* bivalves is at least partially regulated during shell formation, but can vary with ambient pH (Liu et al., 2015). If *Nautilus* similarly regulates the pH and potentially the saturation state of extrapallial fluid during shell precipitation, then variation in $\Delta^{44/40}Ca_{SW\text{-}Shell}$ would be driven by mass-dependent isotope effects during transport into the extrapallial fluid rather than with varying saturation state and precipitation rate at the site of precipitation, as has been observed in inorganic systems. Temperature may affect $\Delta^{44/40}Ca_{SW\text{-}Shell}$ in bivalves (Hippler et al., 2013), although temperature covaries with carbonate saturation state, which can be modulated by the ratio of photosynthesis to respiration (Kelly and Hofmann, 2013; Waldbusser and Salisbury, 2014), and in general, the temperature sensitivity of $\Delta^{44/40}Ca_{SW\text{-}Shell}$

for other carbonate producers is low (Gussone et al., 2006; Gussone and Greifelt, 2019; Gussone and Heuser, 2016; Pretet et al., 2013; Sime et al., 2005)~~.~~.

Seawater likely constitutes the largest source of calcium for the precipitation of *Nautilus m.*~~*macromphalus*~~ shell (Bevelander, 1952; Borght and Puymbroeck, 1966)~~.~~, but complex precipitation and resorption of intermediate carbonate reservoirs could influence shell $\delta^{44/40}$Ca (Boucher-Rodoni and Boucher, 1993)~~.~~. Uroliths represent one proposed intermediary carbonate structure between seawater and shell precipitation (Schipp and Martin, 2010)~~.~~. These structures are amorphous calcium phosphate and have higher Sr contents than the shell wall (Lowenstam et al., 1984)~~.~~. The calcium fractionation factor for this material is unknown, but may be close to -1‰, similar to peloidal calcium phosphate (Gussone and Dietzel, 2016; Schmitt et al., 2003)~~.~~.

Fixed $\Delta^{44/40}Ca_{SW\text{-}Shell}$ in cephalopods (mostly for belemnites) is typically assumed in the reconstruction of $\delta^{44/40}Ca_{SW}$ change through time (Blättler et al., 2012; Farkaš et al., 2007a, 2007b; Hoffmann et al., 2021)~~.~~. Recent evidence suggests that $\Delta^{44/40}Ca_{SW\text{-}Shell}$ for other types of carbonate producers varies with seawater saturation state (Kitch et al., 2021; Linzmeier et al., 2020; Wang et al., 2020) in a way consistent with theoretical models for inorganic calcite formation (DePaolo, 2011; Mills et al., 2021)~~.~~. Any variation of cephalopod $\Delta^{44/40}Ca_{SW\text{-}Shell}$ may reflect additional controls, such as changes in the transport of Ca into the extrapallial fluid (EPF). One possibility is that proportions of Ca transported by selective intracellular calcium channels, non-selective intercellular pathways, or active enzymatic transport (Ca^{2+}-ATPase and carbonic anhydrase) can change. However, under most conditions, selective intracellular calcium channels are thought to dominate transportation of Ca into the EPF through high diffusive fluxes that are selective for Ca (Carré et al., 2006)~~.~~. Another possibility is that any Ca~~calcium~~ isotope fractionation imparted by one or more of these transport mechanisms varies with seawater

saturation state. Future well-controlled aquarium rearing experiments will be necessary to determine if $\Delta^{44/40}Ca_{SW\text{-}Shell}$ changes with carbonate saturation state or another environmental condition.

For anthropogenic ocean acidification to change $\Delta^{44/40}$Ca of these *Nautilus*, higher CO_2 concentrations must propagate to their living depths, which in turn must remain relatively stable throughout the study interval. The observed change in δ^{13}C of shell carbonate (Fig~~.~~ 6B) suggests propagation of anthropogenic carbon into the habitat of the *Nautilus* but does not guarantee a change in the concentration of CO_2 necessary to cause acidification. Boron isotope measurements from shallow water (7-8 m) coral suggests a decrease of ~0.15 pH near New Caledonia since the 1890's, but also indicate similar variations on decadal timescales (Wu et al., 2018)~~;~~. Deeper waters, like those that the *Nautilus* inhabit~~live in~~, are generally expected to acidify more slowly, although organic matter export can sometimes enhance acidification at depth depending on the interaction between deep water ventilation, organic matter export, and general ocean circulation patterns (Chen et al., 2017; Qi et al., 2017)~~;~~. Because the oceans are still in the early stages of acidification, it is likely that the amount of pH change at these depths has not been sufficient to cause detectable alteration in the $\delta^{44/40}$Ca of the studied *Nautilus* specimens.

~~4.2.3~~ Respired CO_2

Formatted: Normal, No bullets or numbering

The proportion of respired carbon incorporated into the shells of mollusks depends on changes in ambient O_2:CO_2 in surrounding waters (McConnaughey et al., 1997; McConnaughey and Gillikin, 2008) and the ~~and~~ metabolic rate (Zhao et al., 2018). Use of the proxy assumes that all carbon used for the precipitation of biogenic carbonates derives from either seawater

dissolved inorganic carbon (DIC) or respired CO_2 derived from food.~~.~~ The $\delta^{13}C$ metabolic rate proxy has been explored using aquarium-reared ~~is more advanced in fish but holds promise for~~ cephalopods, including *Sepia pharaonis* (Chung et al., 2021, 2019b, 2019a). The~~. This~~ quantity of respired carbon contributing to carbonate precipitation can be calculated following a simple relationship outlined by (McConnaughey et al., 1997; McConnaughey and Gillikin, 2008)~~:~~:

$$C_{metabolic} = \frac{\delta^{13}C_{shell} - \varepsilon_{aragonite} - \delta^{13}C_{DIC}}{\delta^{13}C_{metabolic} - \delta^{13}C_{DIC}} \qquad (1)$$

where $\delta^{13}C_{shell}$ refers to the measured value for carbonate shell material. We assume $\delta^{13}C_{metabolic}$ is equivalent to $\delta^{13}C_{org}$ measured in the shell organic matter—given similar findings for other mollusks (Gillikin et al., 2007)~~.~~. The value of $\varepsilon_{aragonite}$ is assumed to be 2.7‰, which is the value for inorganic aragonite (Romanek et al., 1992)~~.~~. To constrain $\delta^{13}C_{DIC}$, we use ~~recently~~ published estimates of $\delta^{13}C_{DIC}$ for preindustrial (1.2~~ ~~‰, VPDB) and recent (1.0~~ ~~‰, VPDB) water near New Caledonia (Eide et al., 2017b, 2017a) at the average *Nautilus m.~~macromphalus~~* living depth of ~400 m (Auclair et al., 2004)~~.~~. This depth is chosen even though shell grows across a range of at least 400 m water depth~~depths~~ (Linzmeier et al., 2016) due to time averaging of bulk samples like those used in this study (Linzmeier, 2019). We assume no ontogenetic change in $\delta^{13}C_{carb}$ biases our results. All samples were from near at the aperture of the body chamber of large shells, and most change in $\delta^{13}C_{carb}$ has been observed in the shell near to hatching rather than later in ontogeny, as is observed in bivalves.~~.~~

Calculations using these metrics show increasing amounts of respired carbon are being incorporated into the shells of *Nautilus m.~~macromphalus~~* during the last 130 years (Fig~~.~~ 9). The observed increase (from ~8 to ~22~~(~15~~%) likely reflects changing metabolic rates caused by transgenerational acclimation to ocean acidification, as has been seen in *Mya arenaria* bivalves

(Zhao et al., 2018)~~,~~. The increase is much less than the observed metabolic carbon increase (27% to 61%) in the experimentally reared bivalves over what is likely to be a much larger pH range (8.1 to 7.7 pH) (Zhao et al., 2018)~~,~~. Other experimentation with *Arctica islandica* bivalves has suggested that ontogenetic variation in the amount of respired carbon incorporated into shells is minimal (Beirne et al., 2012). Because the *Nautilus* live relatively deep, CO_2 incorporation from the atmosphere is likely to be lower than that experienced by shallower organisms. The decrease~~.~~ ~~Decrease~~ of pH at the living depths of these *Nautilus* *m.~~macromphalus~~* may be facilitated by dampened deep water oxygenation related to continued organic matter export (Chen et al., 2017)~~,~~. The observed increase in respired carbon could also be modulated by changes in the ratio of dissolved oxygen to carbon dioxide through expanding oxygen minimum zones offshore of New Caledonia due to increased agricultural intensity and nutrient-rich runoff (Duwig et al., 1998; Gaillard and Manner, 2010)~~;~~, dramatic changes to vegetation (Bouchet et al., 1995)~~;~~, increasing [CO_2] due to anthropogenic injection into the atmosphere-ocean system (Orr et al., 2005)~~;~~, pressure from mining runoff (Pascal et al., 2008)~~;~~, or changing preferred habitat depths due to changing ecosystem composition from fishing other organisms (Fig~~.~~ 3). It is also unlikely that the observed increase in metabolic carbon is caused by ontogenetic change (e.g., because shells represent mature *Nautilus* and most $\delta^{13}C_{carb}$ change occurs very early in ontogeny.

Fig 9. Crossplot of the calculated metabolic carbon contribution vs time. Note the increase in the amount of metabolic carbon incorporated into the shell since the 1970's.

~~4.3~~ Future work

One of the primary challenges to clearly calibrating geochemical proxies in biogenic carbonates is the 'shock' of changing environmental conditions during the experimental treatment of individuals (Clark et al., 2020; Hahn et al., 2012)~~,~~. Although the magnitude of

Formatted: Not Highlight

Formatted: Font: 16 pt

Formatted: Normal, No bullets or numbering

anthropogenic and natural environmental change can be large, both are slow relative the generational timescale~~to generation time~~ of organisms. This means that the best calibrations for proxies preserved in biogenic carbonates may be found in historical or spatially distributed samples that inhabited~~have lived in~~ conditions across several generations (Clark et al., 2020; Telesca et al., 2021)~~.~~. Studies in modern settings may benefit from cross-calibration with low-cost real-time environmental logging (Beddows and Mallon, 2018). Focus on mollusk shell is of particular merit due to wide ranging methods for diagenetic assessment and preservation of shell organic matter .~~.~~ Future work on these isotope systems in cephalopods and other mollusks should leverage museum collections spanning collection time and geographic range to avoid confounding factors of shock and to allow for adaptive responses. ~~Focus on aragonitic mollusk shell is of particular merit due to the wider ranging methods for diagenetic assessment~~ (Cochran et al., 2010; Pederson et al., 2019; Witts et al., 2018)~~, common preservation of original mineralogy~~ (Seuss et al., 2012)~~, and preservation of shell organic matter in mollusks~~ (Clark, 1999; Myers et al., 2018)~~.~~ Further work on internally shelled mollusks (*Sepia* and *Spirula*~~*Spirlua*~~) may be informative for interrogating belemnite archives given newfound microstructural complexity (Benito et al., 2016; Hoffmann et al., 2016)~~.~~.

~~5.~~ Conclusions

Geochemical analyses of subfossil, historical, and modern *Nautilus* *m.*~~*macromphalus*~~ show the importance of calibrating geochemical signals across generations of organisms. The low temperature, potential diagenetic alteration ~~of $\delta^{44/40}$Ca, elemental ratios, and δ^{15}N of organic matter~~ also points~~point~~ to the importance of robust diagenetic assessment of samples from different environments. Our data suggest that there is intergenerational increase in *Nautilus*

Formatted: Font: 18 pt

Formatted: Normal, No bullets or numbering

m.~~*macromphalus*~~ metabolism near New Caledonia, which is likely caused by ocean acidification. Future work using historical and modern marine mollusk shell material will be important for calibrating geochemical proxies across events that can cause both selection and/or phenotypic plasticity response.

Acknowledgements

Formatted: Font: 18 pt

Formatted: Indent: Left: 0"

We would like to thank Jochen Gerber (Field Museum) and Bushra Hussaini (AMNH) for assistance in accessing samples. We also thank three anonymous reviewers~~Funding~~ for their constructive comments to improve this manuscript.~~research was from the Ubben Program for Climate and Carbon Science at Northwestern University (Illinois, USA) and the David and Lucile Packard Foundation (2007–31757).~~

References

Formatted: Font: 18 pt

Formatted: Indent: Left: 0"

Auclair, A.-C., Lecuyer, C., Bucher, H., Sheppard, S.M.F., 2004. Carbon and oxygen isotope composition of *Nautilus macromphalus*: a record of thermocline waters off New Caledonia. Chem. Geol. 207, 91–100. https://doi.org/10.1016/j.chemgeo.2004.02.006

Barord, G.J., Dooley, F., Dunstan, A., Ilano, A., Keister, K.N., Neumeister, H., Preuss, T., Schoepfer, S., Ward, P.D., 2014. Comparative Population Assessments of *Nautilus* sp. in the Philippines, Australia, Fiji, and American Samoa Using Baited Remote Underwater Video Systems. PLoS ONE 9, e100799. https://doi.org/10.1371/journal.pone.0100799

Beddows, P.A., Mallon, E.K., 2018. Cave Pearl Data Logger: A Flexible Arduino-Based Logging Platform for Long-Term Monitoring in Harsh Environments. Sensors 18, 530. https://doi.org/10.3390/s18020530

Beirne, E.C., Wanamaker, A.D., Feindel, S.C., 2012. Experimental validation of environmental controls on the $\delta^{13}C$ of *Arctica islandica* (ocean quahog) shell carbonate. Geochim. Cosmochim. Acta 84, 395–409. https://doi.org/10.1016/j.gca.2012.01.021

Benito, M.I., Reolid, M., Viedma, C., 2016. On the microstructure, growth pattern and original porosity of belemnite rostra: insights from calcitic Jurassic belemnites. J. Iber. Geol. 42, 201–226. https://doi.org/10.5209/revBJIGE.2016.v42.n2.53232

Bevelander, G., 1952. Calcification in mollusks III. Intake and deposition of Ca^{45} and P^{32} in relation to shell formation. Biol. Bull. 102, 9–15. https://doi.org/10.2307/1538617

Blättler, C.L., Henderson, G.M., Jenkyns, H.C., 2012. Explaining the Phanerozoic Ca isotope history of seawater. Geology 40, 843–846. https://doi.org/10.1130/G33191.1

Borght, O.V.D., Puymbroeck, S.V., 1966. Calcium Metabolism in a Freshwater Mollusc : Quantitative Importance of Water and Food as Supply for Calcium During Growth. Nature 210, 791. https://doi.org/10.1038/210791a0

Boucher-Rodoni, R., Boucher, G., 1993. Respiratory quotient and calcification of *Nautilus macromphalus* (Cephalopoda: Nautiloidea). Mar. Biol. 117, 629–633.

Bouchet, P., Jaffre, T., Veillon, J.-M., 1995. Plant extinction in New Caledonia: protection of sclerophyll forests urgently needed. Biodivers. Conserv. 4, 415–428. https://doi.org/10.1007/BF00058425

Buchardt, B., Weiner, S., 1981. Diagenesis of aragonite from Upper Cretaceous ammonites: a geochemical case-study. Sedimentology 28, 423–438. https://doi.org/10.1111/j.1365-3091.1981.tb01691.x

Carré, M., Bentaleb, I., Bruguier, O., Ordinola, E., Barrett, N.T., Fontugne, M., 2006. Calcification rate influence on trace element concentrations in aragonitic bivalve shells: Evidences and mechanisms. Geochim. Cosmochim. Acta 70, 4906–4920. https://doi.org/10.1016/j.gca.2006.07.019

Chen, C.-T.A., Lui, H.-K., Hsieh, C.-H., Yanagi, T., Kosugi, N., Ishii, M., Gong, G.-C., 2017. Deep oceans may acidify faster than anticipated due to global warming. Nat. Clim. Change 7, 890. https://doi.org/10.1038/s41558-017-0003-y

Chung, M.-T., Chen, C.-Y., Shiao, J.-C., Shirai, K., Wang, C.-H., 2021. Metabolic proxy for cephalopods: Stable carbon isotope values recorded in different biogenic carbonates. Methods Ecol. Evol. 12, 1648–1657. https://doi.org/10.1111/2041-210X.13630

Chung, M.-T., Trueman, C.N., Godiksen, J.A., Grønkjær, P., 2019a. Otolith $\delta^{13}C$ values as a metabolic proxy: approaches and mechanical underpinnings. Mar. Freshw. Res. https://doi.org/10.1071/MF18317

Chung, M.-T., Trueman, C.N., Godiksen, J.A., Holmstrup, M.E., Grønkjær, P., 2019b. Field metabolic rates of teleost fishes are recorded in otolith carbonate. Commun. Biol. 2, 24. https://doi.org/10.1038/s42003-018-0266-5

Cinner, J.E., Zamborain-Mason, J., Gurney, G.G., Graham, N.A.J., MacNeil, M.A., Hoey, A.S., Mora, C., Villéger, S., Maire, E., McClanahan, T.R., Maina, J.M., Kittinger, J.N., Hicks, C.C., 2020. Meeting fisheries, ecosystem function, and biodiversity goals in a human-dominated world 6.

Clark, G.R.I., 1999. Organic matrix taphonomy in some molluscan shell microstructures. Palaeogeogr. Palaeoclimatol. Palaeoecol. 149, 305–312.

Clark, M.S., Peck, L.S., Arivalagan, J., Backeljau, T., Berland, S., Cardoso, J.C.R., Caurcel, C., Chapelle, G., Noia, M.D., Dupont, S., Gharbi, K., Hoffman, J.I., Last, K.S., Marie, A., Melzner, F., Michalek, K., Morris, J., Power, D.M., Ramesh, K., Sanders, T., Sillanpää, K., Sleight, V.A., Stewart- Sinclair, P.J., Sundell, K., Telesca, L., Vendrami, D.L.J., Ventura, A., Wilding, T.A., Yarra, T., Harper, E.M., 2020. Deciphering mollusc shell production: the roles of genetic mechanisms through to ecology, aquaculture and biomimetics. Biol. Rev. 95, 1812–1837. https://doi.org/10.1111/brv.12640

Cochran, J.K., Kallenberg, K., Landman, N.H., Harries, P.J., Weinreb, D., Turekian, K.K., Beck, A.J., Cobban, W.A., 2010. Effect of diagenesis on the Sr, O, and C isotope composition of Late Cretaceous mollusks from the Western Interior Seaway of North America. Am. J. Sci. 310, 69–88. https://doi.org/10.2475/02.2010.01

Cravatte, S., Kestenare, E., Eldin, G., Ganachaud, A., Lefèvre, J., Marin, F., Menkes, C., Aucan, J., 2015. Regional circulation around New Caledonia from two decades of observations. J. Mar. Syst. 148, 249–271. https://doi.org/10.1016/j.jmarsys.2015.03.004

Crocker, K.C., DeNiro, M.J., Ward, P.D., 1985. Stable isotopic investigations of early development in extant and fossil chambered cephalopods I. Oxygen isotopic composition of eggwater and carbon isotopic composition of siphuncle organic matter in Nautilus. Geochim. Cosmochim. Acta 49, 2527–2532. https://doi.org/10.1016/0016-7037(85)90120-6

DePaolo, D.J., 2011. Surface kinetic model for isotopic and trace element fractionation during precipitation of calcite from aqueous solutions. Geochim. Cosmochim. Acta 75, 1039–1056. https://doi.org/10.1016/j.gca.2010.11.020

Doney, S.C., Fabry, V.J., Feely, R.A., Kleypas, J.A., 2009. Ocean Acidification: The Other CO2 Problem. Annu. Rev. Mar. Sci. 1, 169–192. https://doi.org/10.1146/annurev.marine.010908.163834

Duwig, C., Becquer, T., Clothier, B.E., Vauclin, M., 1998. Nitrate leaching through oxisols of the Loyalty Islands (New Caledonia) under intensified agricultural practices. Geoderma 84, 29–43. https://doi.org/10.1016/S0016-7061(97)00119-5

Eide, M., Olsen, A., Ninnemann, U.S., Eldevik, T., 2017a. A global estimate of the full oceanic 13C Suess effect since the preindustrial. Glob. Biogeochem. Cycles 31, 492–514. https://doi.org/10.1002/2016GB005472

Eide, M., Olsen, A., Ninnemann, U.S., Johannessen, T., 2017b. A global ocean climatology of preindustrial and modern ocean δ^{13}C: Climatology of Preindustrial δ^{13}C. Glob. Biogeochem. Cycles 31, 515–534. https://doi.org/10.1002/2016GB005473

Ellis, N.M., Tobin, T.S., 2019. Evidence for seasonal variation in δ^{13}C and δ^{18}O profiles of *Baculites* and implications for growth rate. Palaeontology 62, 583–598. https://doi.org/10.1111/pala.12416

Etheridge, D.M., Steele, L.P., Langenfelds, R.L., Francey, R.J., Barnola, J.M., Morgan, V.I., 1998. Historical CO2 records from the Law Dome DE08, DE08-2, and DSS ice cores. Trends Compend. Data Glob. Change 351–364.

Eyre, B.D., Cyronak, T., Drupp, P., Carlo, E.H.D., Sachs, J.P., Andersson, A.J., 2018. Coral reefs will transition to net dissolving before end of century. Science 359, 908–911. https://doi.org/10.1126/science.aao1118

Farkaš, J., Böhm, F., Wallmann, K., Blenkinsop, J., Eisenhauer, A., van Geldern, R., Munnecke, A., Voigt, S., Veizer, J., 2007a. Calcium isotope record of Phanerozoic oceans: Implications for chemical evolution of seawater and its causative mechanisms. Geochim. Cosmochim. Acta 71, 5117–5134. https://doi.org/10.1016/j.gca.2007.09.004

Farkaš, J., Buhl, D., Blenkinsop, J., Veizer, J., 2007b. Evolution of the oceanic calcium cycle during the late Mesozoic: Evidence from $\delta^{44/40}$Ca of marine skeletal carbonates. Earth Planet. Sci. Lett. 253, 96–111. https://doi.org/10.1016/j.epsl.2006.10.015

Fennel, K., Testa, J.M., 2019. Biogeochemical Controls on Coastal Hypoxia. Annu. Rev. Mar. Sci. 11, 105–130. https://doi.org/10.1146/annurev-marine-010318-095138

Ferguson, K., Macleod, K.G., Landman, N.H., Sessa, J.A., 2019. Evaluating growth and ecology in baculitid and scaphitid ammonites using stable isotope sclerochronology. PALAIOS 34, 317–329. https://doi.org/10.2110/palo.2019.005

Gaillard, C., Manner, H.I., 2010. Yam Cultivation on the East Coast of New Caledonia: adaptation of agriculture to social and economic changes. Aust. Geogr. 41, 485–505. https://doi.org/10.1080/00049182.2010.519697

Gillikin, D.P., Lorrain, A., Meng, L., Dehairs, F., 2007. A large metabolic carbon contribution to the $\delta^{13}C$ record in marine aragonitic bivalve shells. Geochim. Cosmochim. Acta 71, 2936–2946. https://doi.org/10.1016/j.gca.2007.04.003

Guillemot, N., Léopold, M., Cuif, M., Chabanet, P., 2009. Characterization and management of informal fisheries confronted with socio-economic changes in New Caledonia (South Pacific). Fish. Res. 98, 51–61. https://doi.org/10.1016/j.fishres.2009.03.013

Gussone, N., Dietzel, M., 2016. Calcium Isotope Fractionation During Mineral Precipitation from Aqueous Solution, in: Calcium Stable Isotope Geochemistry. Springer Berlin Heidelberg, Berlin, Heidelberg, pp. 75–110. https://doi.org/10.1007/978-3-540-68953-9_3

Gussone, N., Greifelt, T., 2019. Incorporation of Ca isotopes in carapaxes of marine ostracods. Chem. Geol. 510, 130–139. https://doi.org/10.1016/j.chemgeo.2019.02.012

Gussone, N., Heuser, A., 2016. Biominerals and Biomaterial, in: Calcium Stable Isotope Geochemistry. Springer Berlin Heidelberg, Berlin, Heidelberg, pp. 111–144. https://doi.org/10.1007/978-3-540-68953-9_4

Gussone, N., Langer, G., Thoms, S., Nehrke, G., Eisenhauer, A., Riebesell, U., Wefer, G., 2006. Cellular calcium pathways and isotope fractionation in *Emiliania huxleyi*. Geology 34, 625–628. https://doi.org/10.1130/G22733.1

Hahn, S., Rodolfo-Metalpa, R., Griesshaber, E., Schmahl, W.W., Buhl, D., Hall-Spencer, J.M., Baggini, C., Fehr, K.T., Immenhauser, A., 2012. Marine bivalve shell geochemistry and ultrastructure from modern low pH environments: Environmental effect versus experimental bias. https://doi.org/10.5194/bg-9-1897-2012

Heuser, A., Tütken, T., Gussone, N., Galer, S.J.G., 2011. Calcium isotopes in fossil bones and teeth — Diagenetic versus biogenic origin. Geochim. Cosmochim. Acta 75, 3419–3433. https://doi.org/10.1016/j.gca.2011.03.032

Hippler, D., Witbaard, R., van Aken, H.M., Buhl, D., Immenhauser, A., 2013. Exploring the calcium isotope signature of *Arctica islandica* as an environmental proxy using laboratory- and field-cultured specimens. Palaeogeogr. Palaeoclimatol. Palaeoecol., Unraveling environmental histories from skeletal diaries - advances in sclerochronology 373, 75–87. https://doi.org/10.1016/j.palaeo.2011.11.015

Hoffmann, R., Richter, D.K., Neuser, R.D., Jöns, N., Linzmeier, B.J., Lemanis, R.E., Fusseis, F., Xiao, X., Immenhauser, A., 2016. Evidence for a composite organic–inorganic fabric of belemnite rostra: Implications for palaeoceanography and palaeoecology. Sediment. Geol. 341, 203–215. https://doi.org/10.1016/j.sedgeo.2016.06.001

Hoffmann, R., Riechelmann, S., Liebetrau, V., Eisenhauer, A., Immenhauser, A., 2021. Calcium isotope values of modern and fossil cephalopod shells – Trophic level or proxy for seawater geochemistry? Chem. Geol. 583, 120466. https://doi.org/10.1016/j.chemgeo.2021.120466

Holmden, C., Papanastassiou, D.A., Blanchon, P., Evans, S., 2012. $\delta^{44/40}Ca$ variability in shallow water carbonates and the impact of submarine groundwater discharge on Ca-cycling in marine environments. Geochim. Cosmochim. Acta 83, 179–194. https://doi.org/10.1016/j.gca.2011.12.031

House, M.R., 2010. Geographic Distribution of Nautilus Shells, in: Saunders, W.B., Landman, N.H. (Eds.), Nautilus: The Biology and Paleobiology of a Living Fossil, Reprint with Additions, Topics in Geobiology. Springer Netherlands, Dordrecht, pp. 53–64. https://doi.org/10.1007/978-90-481-3299-7_4

Immenhauser, A., Schöne, B.R., Hoffmann, R., Niedermayr, A., 2016. Mollusc and brachiopod skeletal hard parts: Intricate archives of their marine environment. Sedimentology 63, 1–59. https://doi.org/10.1111/sed.12231

Junium, C.K., Arthur, M.A., 2007. Nitrogen cycling during the Cretaceous, Cenomanian-Turonian Oceanic Anoxic Event II. Geochem. Geophys. Geosystems 8. https://doi.org/10.1029/2006GC001328

Kashiyama, Y., Ogawa, N.O., Chikaraishi, Y., Kashi, N., Sakai, S., Tanabe, K., Ohkouchi, N., 2010. Reconstructing the life history of modern and fossil nautiloids based on the nitrogen isotopic composition of shell organic matter and amino acids, in: Tanabe, K., Shigeta, Y., Sasaki, T., Hirano, H. (Eds.), Cephalopods - Present and Past. Tokai University Press, Tokyo, pp. 67–75.

Kelly, M.W., Hofmann, G.E., 2013. Adaptation and the physiology of ocean acidification. Funct. Ecol. 27, 980–990. https://doi.org/10.1111/j.1365-2435.2012.02061.x

Kidwell, S.M., 2002. Time-averaged molluscan death assemblages: Palimpsests of richness, snapshots of abundance. Geology 30, 803–806. https://doi.org/10.1130/0091-7613(2002)030<0803:TAMDAP>2.0.CO;2

Kitch, G.D., Jacobson, A.D., Harper, D.T., Hurtgen, M.T., Sageman, B.B., Zachos, J.C., 2021. Calcium isotope composition of *Morozovella* over the latePaleocene–early Eocene. Geology. https://doi.org/10.1130/G48619.1

Kowalewski, M., Casebolt, S., Hua, Q., Whitacre, K.E., Kaufman, D.S., Kosnik, M.A., 2018. One fossil record, multiple time resolutions: Disparate time-averaging of echinoids and mollusks on a Holocene carbonate platform. Geology 46, 51–54. https://doi.org/10.1130/G39789.1

Kroeker, K.J., Kordas, R.L., Harley, C.D.G., 2017. Embracing interactions in ocean acidification research: confronting multiple stressor scenarios and context dependence. Biol. Lett. 13, 20160802. https://doi.org/10.1098/rsbl.2016.0802

Kröger, B., Vinther, J., Fuchs, D., 2011. Cephalopod origin and evolution: A congruent picture emerging from fossils, development and molecules. BioEssays 33, 602–613. https://doi.org/10.1002/bies.201100001

Landman, N.H., Cochran, J.K., 2010. Growth and Longevity of *Nautilus*, in: Nautilus, Topics in Geobiology. Springer, Dordrecht, pp. 401–420. https://doi.org/10.1007/978-90-481-3299-7_28

Landman, N.H., Cochran, J.K., Rye, D.M., Tanabe, K., Arnold, J.M., 1994. Early life history of *Nautilus*: evidence from isotopic analyses of aquarium-reared specimens. Paleobiology 20, 40–51.

Landman, N.H., Cochran, J.K., Slovacek, M., Larson, N.L., Garb, M.P., Brezina, J., Witts, J.D., 2018. Isotope sclerochronology of ammonites (*Baculites compressus*) from methane seep and non-seep sites in the Late Cretaceous Western Interior Seaway, USA: Implications for ammonite habitat and mode of life. Am. J. Sci. 318, 603–639. https://doi.org/10.2475/06.2018.01

Landman, N.H., Mapes, R.H., Cochran, J.K., Lignier, V., Hembree, D.I., Goiran, C., Folcher, E., Brunet, P., 2014. An unusual occurrence of *Nautilus macromphalus* in a Cenote in the

Loyalty Islands (New Caledonia). PLOS ONE 9, e113372. https://doi.org/10.1371/journal.pone.0113372

Lehn, G.O., Jacobson, A.D., Holmden, C., 2013. Precise analysis of Ca isotope ratios ($\delta^{44/40}Ca$) using an optimized ^{43}Ca–^{42}Ca double-spike MC-TIMS method. Int. J. Mass Spectrom. 351, 69–75. https://doi.org/10.1016/j.ijms.2013.06.013

Linzmeier, B.J., 2019. Refining the interpretation of oxygen isotope variability in free-swimming organisms. Swiss J. Palaeontol. 138, 109–121. https://doi.org/10.1007/s13358-019-00187-3

Linzmeier, B.J., Jacobson, A.D., Sageman, B.B., Hurtgen, M.T., Ankney, M.E., Petersen, S.V., Tobin, T.S., Kitch, G.D., Wang, J., 2020. Calcium isotope evidence for environmental variability before and across the Cretaceous-Paleogene mass extinction. Geology 48, 34–38. https://doi.org/10.1130/G46431.1

Linzmeier, B.J., Kozdon, R., Peters, S.E., Valley, J.W., 2016. Oxygen isotope variability within *Nautilus* shell growth bands. PLOS ONE 11, e0153890. https://doi.org/10.1371/journal.pone.0153890

Linzmeier, B.J., Landman, N.H., Peters, S.E., Kozdon, R., Kitajima, K., Valley, J.W., 2018. Ion microprobe–measured stable isotope evidence for ammonite habitat and life mode during early ontogeny. Paleobiology 44, 684–708. https://doi.org/10.1017/pab.2018.21

Liu, Y.-W., Aciego, S.M., Wanamaker, A.D., 2015. Environmental controls on the boron and strontium isotopic composition of aragonite shell material of cultured *Arctica islandica*. Biogeosciences 12, 3351–3368. https://doi.org/10.5194/bg-12-3351-2015

Lowenstam, H.A., Traub, W., Weiner, S., 1984. *Nautilus* hard parts: a study of the mineral and organic constitutents. Paleobiology 10, 268–279.

Lukeneder, A., Harzhauser, M., Müllegger, S., Piller, W.E., 2010. Ontogeny and habitat change in Mesozoic cephalopods revealed by stable isotopes ($\delta^{18}O$, $\delta^{13}C$). Earth Planet. Sci. Lett. 296, 103–114. https://doi.org/10.1016/j.epsl.2010.04.053

Martin, J.E., Vincent, P., Tacail, T., Khaldoune, F., Jourani, E., Bardet, N., Balter, V., 2017. Calcium Isotopic Evidence for Vulnerable Marine Ecosystem Structure Prior to the K/Pg Extinction. Curr. Biol. 27, 1641-1644.e2. https://doi.org/10.1016/j.cub.2017.04.043

McConnaughey, T.A., Burdett, J., Whelan, J.F., Paull, C.K., 1997. Carbon isotopes in biological carbonates: Respiration and photosynthesis. Geochim. Cosmochim. Acta 61, 611–622. https://doi.org/10.1016/S0016-7037(96)00361-4

McConnaughey, T.A., Gillikin, D.P., 2008. Carbon isotopes in mollusk shell carbonates. Geo-Mar. Lett. 28, 287–299. https://doi.org/10.1007/s00367-008-0116-4

Milano, S., Prendergast, A.L., Schöne, B.R., 2016. Effects of cooking on mollusk shell structure and chemistry: Implications for archeology and paleoenvironmental reconstruction. J. Archaeol. Sci. Rep. 7, 14–26. https://doi.org/10.1016/j.jasrep.2016.03.045

Mills, J.V., DePaolo, D.J., Lammers, L.N., 2021. The influence of Ca:CO_3 stoichiometry on Ca isotope fractionation: Implications for process-based models of calcite growth. Geochim. Cosmochim. Acta 298, 87–111. https://doi.org/10.1016/j.gca.2021.01.016

Moriya, K., Nishi, H., Kawahata, H., Tanabe, K., Takayanagi, Y., 2003. Demersal habitat of Late Cretaceous ammonoids: Evidence from oxygen isotopes for the Campanian (Late Cretaceous) northwestern Pacific thermal structure. Geology 31, 167–170.

Mutvei, H., 1972. Ultrastructural relationships between the prismatic and nacreous layers in *Nautilus* (Cephalopoda). Biomineralization 4, 81–86.

Mutvei, H., 1964. On the shells of *Nautilus* and *Spirula*: with notes on shell secretion in non-cephalopod molluscs. Ark. För Zool. 16, 221–278.
Myers, C.E., Bergmann, K.D., Sun, C.-Y., Boekelheide, N., Knoll, A.H., Gilbert, P.U.P.A., 2018. Exceptional preservation of organic matrix and shell microstructure in a Late Cretaceous *Pinna* fossil revealed by photoemission electron spectromicroscopy. Geology 46, 711–714. https://doi.org/10.1130/G45271.1
Newsome, S.D., Tinker, M.T., Monson, D.H., Oftedal, O.T., Ralls, K., Staedler, M.M., Fogel, M.L., Estes, J.A., 2009. Using Stable Isotopes to Investigate Individual Diet Specialization in California Sea Otters (*Enhydra lutris nereis*). Ecology 90, 961–974.
Newsome, S.D., Yeakel, J.D., Wheatley, P.V., Tinker, M.T., 2012. Tools for quantifying isotopic niche space and dietary variation at the individual and population level. J. Mammal. 93, 329–341. https://doi.org/10.1644/11-MAMM-S-187.1
Orr, J.C., Fabry, V.J., Aumont, O., Bopp, L., Doney, S.C., Feely, R.A., Gnanadesikan, A., Gruber, N., Ishida, A., Joos, F., Key, R.M., Lindsay, K., Maier-Reimer, E., Matear, R., Monfray, P., Mouchet, A., Najjar, R.G., Plattner, G.-K., Rodgers, K.B., Sabine, C.L., Sarmiento, J.L., Schlitzer, R., Slater, R.D., Totterdell, I.J., Weirig, M.-F., Yamanaka, Y., Yool, A., 2005. Anthropogenic ocean acidification over the twenty-first century and its impact on calcifying organisms. Nature 437, 681. https://doi.org/10.1038/nature04095
Pascal, M., Forges, B.R.D., Guyader, H.L., Simberloff, D., 2008. Mining and Other Threats to the New Caledonia Biodiversity Hotspot. Conserv. Biol. 22, 498–499. https://doi.org/10.1111/j.1523-1739.2008.00889.x
Pederson, C., Mavromatis, V., Dietzel, M., Rollion-Bard, C., Nehrke, G., Jöns, N., Jochum, K.P., Immenhauser, A., 2019. Diagenesis of mollusc aragonite and the role of fluid reservoirs. Earth Planet. Sci. Lett. 514, 130–142. https://doi.org/10.1016/j.epsl.2019.02.038
Pretet, C., Samankassou, E., Felis, T., Reynaud, S., Böhm, F., Eisenhauer, A., Ferrier-Pagès, C., Gattuso, J.-P., Camoin, G., 2013. Constraining calcium isotope fractionation (δ44/40Ca) in modern and fossil scleractinian coral skeleton. Chem. Geol. 340, 49–58. https://doi.org/10.1016/j.chemgeo.2012.12.006
Price, G.D., Twitchett, R.J., Wheeley, J.R., Buono, G., 2013. Isotopic evidence for long term warmth in the Mesozoic. Sci. Rep. 3. https://doi.org/10.1038/srep01438
Qi, D., Chen, L., Chen, B., Gao, Z., Zhong, W., Feely, R.A., Anderson, L.G., Sun, H., Chen, J., Chen, M., Zhan, L., Zhang, Y., Cai, W.-J., 2017. Increase in acidifying water in the western Arctic Ocean. Nat. Clim. Change 7, 195–199. https://doi.org/10.1038/nclimate3228
Ries, J.B., Ghazaleh, M.N., Connolly, B., Westfield, I., Castillo, K.D., 2016. Impacts of seawater saturation state (ΩA=0.4–4.6) and temperature (10, 25°C) on the dissolution kinetics of whole-shell biogenic carbonates. Geochim. Cosmochim. Acta 192, 318–337. https://doi.org/10.1016/j.gca.2016.07.001
Romanek, C.S., Grossman, E.L., Morse, J.W., 1992. Carbon isotopic fractionation in synthetic aragonite and calcite: Effects of temperature and precipitation rate. Geochim. Cosmochim. Acta 56, 419–430. https://doi.org/10.1016/0016-7037(92)90142-6
Saunders, W.B., 2010. The Species of *Nautilus*, in: Nautilus, Topics in Geobiology. Springer, Dordrecht, pp. 35–52. https://doi.org/10.1007/978-90-481-3299-7_3
Saunders, W.B., Ward, P.D., 2010. Ecology, Distribution, and Population Characteristics of *Nautilus*, in: Nautilus, Topics in Geobiology. Springer, Dordrecht, pp. 137–162. https://doi.org/10.1007/978-90-481-3299-7_9

Schimmelmann, A., Albertino, A., Sauer, P.E., Qi, H., Molinie, R., Mesnard, F., 2009. Nicotine, acetanilide and urea multi-level 2H-, 13C- and 15N-abundance reference materials for continuous-flow isotope ratio mass spectrometry. Rapid Commun. Mass Spectrom. 23, 3513–3521. https://doi.org/10.1002/rcm.4277
Schipp, R., Martin, A.W., 2010. The Excretory System of Nautilus, in: Saunders, W.B., Landman, N.H. (Eds.), Nautilus: The Biology and Paleobiology of a Living Fossil, Reprint with Additions, Topics in Geobiology. Springer Netherlands, Dordrecht, pp. 281–304. https://doi.org/10.1007/978-90-481-3299-7_20
Schmitt, A.-D., Stille, P., Vennemann, T., 2003. Variations of the $^{44}Ca/^{40}Ca$ ratio in seawater during the past 24 million years: evidence from $\delta^{44}Ca$ and $\delta^{18}O$ values of Miocene phosphates. Geochim. Cosmochim. Acta 67, 2607–2614. https://doi.org/10.1016/S0016-7037(03)00100-5
Schunter, C., Welch, M.J., Nilsson, G.E., Rummer, J.L., Munday, P.L., Ravasi, T., 2018. An interplay between plasticity and parental phenotype determines impacts of ocean acidification on a reef fish. Nat. Ecol. Evol. 2, 334–342. https://doi.org/10.1038/s41559-017-0428-8
Sessa, J.A., Larina, E., Knoll, K., Garb, M., Cochran, J.K., Huber, B.T., MacLeod, K.G., Landman, N.H., 2015. Ammonite habitat revealed via isotopic composition and comparisons with co-occurring benthic and planktonic organisms. Proc. Natl. Acad. Sci. 112, 15562–15567. https://doi.org/10.1073/pnas.1507554112
Seuss, B., Titschack, J., Seifert, S., Neubauer, J., Nützel, A., 2012. Oxygen and stable carbon isotopes from a nautiloid from the middle Pennsylvanian (Late Carboniferous) impregnation Lagerstätte 'Buckhorn Asphalt Quarry' — Primary paleo-environmental signals versus diagenesis. Palaeogeogr. Palaeoclimatol. Palaeoecol. 319–320, 1–15. https://doi.org/10.1016/j.palaeo.2011.12.008
Sime, N.G., De La Rocha, C.L., Galy, A., 2005. Negligible temperature dependence of calcium isotope fractionation in 12 species of planktonic foraminifera. Earth Planet. Sci. Lett. 232, 51–66. https://doi.org/10.1016/j.epsl.2005.01.011
Skulan, J., DePaolo, D.J., 1999. Calcium isotope fractionation between soft and mineralized tissues as a monitor of calcium use in vertebrates. Proc. Natl. Acad. Sci. 96, 13709–13713. https://doi.org/10.1073/pnas.96.24.13709
Stevenson, J., Dodson, J.R., Prosser, I.P., 2001. A late Quaternary record of environmental change and human impact from New Caledonia. Palaeogeogr. Palaeoclimatol. Palaeoecol. 168, 97–123. https://doi.org/10.1016/S0031-0182(00)00251-0
Swart, P.K., 2015. The geochemistry of carbonate diagenesis: The past, present and future. Sedimentology 62, 1233–1304. https://doi.org/10.1111/sed.12205
Telesca, L., Peck, L.S., Backeljau, T., Heinig, M.F., Harper, E.M., 2021. A century of coping with environmental and ecological changes via compensatory biomineralization in mussels. Glob. Change Biol. 27, 624–639. https://doi.org/10.1111/gcb.15417
Tomašových, A., Schlögl, J., Biroň, A., Hudáčková, N., Mikuš, T., 2017. Taphonomic clock and bathymetric dependence of cephalopod preservation in bathyal, sediment-starved environments. PALAIOS 32, 135–152. https://doi.org/10.2110/palo.2016.039
Urban, M.C., Bocedi, G., Hendry, A.P., Mihoub, J.-B., Pe'er, G., Singer, A., Bridle, J.R., Crozier, L.G., Meester, L.D., Godsoe, W., Gonzalez, A., Hellmann, J.J., Holt, R.D., Huth, A., Johst, K., Krug, C.B., Leadley, P.W., Palmer, S.C.F., Pantel, J.H., Schmitz, A.,

Zollner, P.A., Travis, J.M.J., 2016. Improving the forecast for biodiversity under climate change. Science 353. https://doi.org/10.1126/science.aad8466

Urey, H.C., Lowenstam, H.A., Epstein, S., McKinney, C.R., 1951. Measurement of paleotemperatures and temperatures of the Upper Cretaceous of England, Denmark, and the Southeastern United States. GSA Bull. 62, 399–416. https://doi.org/10.1130/0016-7606(1951)62[399:MOPATO]2.0.CO;2

Waldbusser, G.G., Salisbury, J.E., 2014. Ocean acidification in the Coastal Zone from an organism's perspective: Multiple system parameters, frequency domains, and habitats. Annu. Rev. Mar. Sci. 6, 221–247. https://doi.org/10.1146/annurev-marine-121211-172238

Wang, J., Jacobson, A.D., Sageman, B.B., Hurtgen, M.T., 2020. Stable Ca and Sr isotopes support volcanically triggered biocalcification crisis during Oceanic Anoxic Event 1a. Geology. https://doi.org/10.1130/G47945.1

Ward, P.D., 1985. Periodicity of chamber formation in chambered cephalopods: Evidence from *Nautilus macromphalus* and *Nautilus pompilius*. Paleobiology 11, 438–450.

Weiner, S., Lowenstam, H.A., Hood, L., 1976. Characterization of 80-million-year-old mollusk shell proteins. Proc. Natl. Acad. Sci. 73, 2541–2545. https://doi.org/10.1073/pnas.73.8.2541

Witts, J.D., Newton, R.J., Mills, B.J.W., Wignall, P.B., Bottrell, S.H., Hall, J.L.O., Francis, J.E., Alistair Crame, J., 2018. The impact of the Cretaceous–Paleogene (K–Pg) mass extinction event on the global sulfur cycle: Evidence from Seymour Island, Antarctica. Geochim. Cosmochim. Acta 230, 17–45. https://doi.org/10.1016/j.gca.2018.02.037

Wu, H.C., Dissard, D., Douville, E., Blamart, D., Bordier, L., Tribollet, A., Le Cornec, F., Pons-Branchu, E., Dapoigny, A., Lazareth, C.E., 2018. Surface ocean pH variations since 1689 CE and recent ocean acidification in the tropical South Pacific. Nat. Commun. 9, 2543. https://doi.org/10.1038/s41467-018-04922-1

Young, J.Z., 2010. The Central Nervous System, in: Saunders, W.B., Landman, N.H. (Eds.), Nautilus: The Biology and Paleobiology of a Living Fossil, Reprint with Additions, Topics in Geobiology. Springer Netherlands, Dordrecht, pp. 215–221. https://doi.org/10.1007/978-90-481-3299-7_13

Zhao, L., Yang, F., Milano, S., Han, T., Walliser, E.O., Schöne, B.R., 2018. Transgenerational acclimation to seawater acidification in the Manila clam Ruditapes philippinarum: Preferential uptake of metabolic carbon. Sci. Total Environ. 627, 95–103. https://doi.org/10.1016/j.scitotenv.2018.01.225

Figure 1. Overview of Nautilus macromphalus shell (ROM 48997) with annotation of major shell structures, growth direction, and shell layers. Nautilus shells grow in a spiral and septa are secreted to allow for the maintenance of neutral buoyance during growth through the removal of liquid from the chambers (Ward, 1985). *Shells comprise two major structural forms of aragonite, the inner nacreous layer and outer spherulitic prismatic layer* (Mutvei, 1972, 1964).

Figure 2. **Map showing the sample collection location and major currents surrounding New Caledonia.** A) World map showing the location of New Caledonia in the Southwestern Pacific Ocean. B) Local map showing New Caledonia and the surrounding islands with a star indicating the location of the cenote. *All cenote specimens are from Lifou, Loyalty Islands* (Landman et al., 2014). *All other specimens were collected near the large island and are recent museum specimens. Reliable accounts of the live collection of Nautilus macromphalus are mostly known from areas close to New Caledonia, and the species is thought to have a geographic range restricted to that region* (House, 2010). *Local currents are overlain for reference* (Cravatte et al., 2015).

Figure 3. **Timeline for sample ages, pCO_2, and human settlement of New Caledonia.** Purple vertical bars represent the age of specimens used in this study with the museum repositories noted. *Yellow vertical bar represents indigenous settlement as demonstrated by landscape change observed in lacustrine sediments ~3000 BCE* (Stevenson et al., 2001). French invasion occurred in the mid-1800's. *The comparative record of pCO_2 change showing industrial increases is from Law Dome, East Antarctica* (Etheridge et al., 1998).

Formatted: Font: Bold, Not Italic

Formatted: Font: Not Italic

Formatted: Font: Bold, Not Italic

Formatted: Font: Not Italic

Formatted: Font: Not Italic

~~*Figure 4. Crossplots of Sr/Ca and Mg/Ca ratios vs isotope ratios measured on shell carbonate.*~~ ~~A) $\delta^{44/40}$Ca vs Mg/Ca, B) $\delta^{44/40}$Ca vs Sr/Ca, C) $\delta^{13}C_{shell}$ vs Mg/Ca, D) $\delta^{13}C_{shell}$ vs Sr/Ca, E) $\delta^{18}O_{shell}$ vs Mg/Ca, F) $\delta^{18}O_{shell}$ vs Mg/Ca, G) δ^{15}N vs Mg/Ca, H) δ^{15}N vs Sr/Ca, I) $\delta^{13}C_{org}$ vs Mg/Ca, and J) $\delta^{13}C_{org}$ vs Sr/Ca. The cenote and historical samples distinctly differ along Mg/Ca, Sr/Ca, δ^{15}N, $\delta^{13}C_{org}$, and $\delta^{44/40}$Ca axes.~~

~~*Figure 5. Crossplot of carbon and oxygen isotope ratios measured on shell carbonate.*~~

~~*Figure 6. Stable isotope ratios through time.*~~ ~~A) Shell $\delta^{44/40}$Ca through time. B) The δ^{13}C of shell carbonate through time. C) The δ^{18}O of shell carbonate through time. D) The δ^{15}N of shell organic matter though time. E) The δ^{13}C of shell organic matter through time.~~

~~*Figure 7. Isotope ratios of organic matter and the C/N ratio of the organic matter. A) $\delta^{13}C_{org}$ vs $\delta^{15}N_{org}$, B) C/N vs $\delta^{15}N_{org}$.*~~

Formatted: Font: Not Italic

Formatted: Font: Not Italic

Formatted: Font: Not Italic

Formatted: Font: Not Italic

Formatted: Font: Not Italic

Formatted: Font: Not Italic

Formatted: Font: Not Italic

Formatted: Font: Bold, Not Italic

Formatted: Font: Not Italic

Formatted: Font: Not Italic

Formatted: Font: Not Italic

~~*Figure 8. Crossplots of isotope ratios measured in shell organic matter vs shell carbonate*. A) $\delta^{13}C_{org}$ vs $\delta^{13}C_{shell}$; B) $\delta^{15}N$ vs $\delta^{13}C_{shell}$; C) $\delta^{13}C_{org}$ vs $\delta^{18}O_{shell}$; D) $\delta^{15}N$ vs $\delta^{18}O_{shell}$; E) $\delta^{13}C_{org}$ vs $\delta^{44/40}Ca$, and F) $\delta^{15}N$ vs $\delta^{44/40}Ca$.~~

Formatted: Font: Not Italic

Formatted: Font: Not Italic

Formatted: Font: Not Italic

Formatted: Font: Not Italic

Formatted: Font: Not Italic

Supporting information

S1 File. Spreadsheet with all analytical data for all samples. Each tab contains one group of analyses.

~~*Figure 9. Crossplot of the calculated metabolic carbon contribution vs time.*~~

Reviewer #1: Dear authors,

In their manuscript, Linzmeier et al. present a multi-proxy dataset measured in modern and sub-fossil Nautilus macromphalus shell carbonate. The goal of the study is to investigate the effect of several environmental parameters on the shell composition of N. macromphalus to provide a baseline for studies on historical and fossil specimens of this and related species aiming to reconstruct environmental change. The authors present a valuable dataset of natural variability as recorded in Nautilus shells which complements previous culture experiments. The decision to work on shells from historical collection is interesting, as it avoids certain complications of culture experiments (most notably the shock to the animals due to changing aquarium conditions) as well as the ethical considerations of experimenting on live cephalopods. Overall, I believe this manuscript merits publication barring some moderate revisions, mostly pertaining to the methodology and the presentation of the data, which I will highlight in my line-by-line comments below:

We thank the reviewer for their work on our manuscript. We have carefully addressed your comments in the document below and in the revised manuscript.

Line 42: "isotope systematics of light stable isotopes" rephrase to "systematics of light stable isotopes"

Done.

Line 92: The authors should provide the uncertainty on the radiocarbon date of the cenote specimen in the text

Done.

Lines 104-115: Recently, there has been some discussion in the literature about the pre-treatment methods required to distinguish between the contributions of skeletal carbonate-bound organic matter and non-carbonate bound organic matter phases in d13C and d15N analyses of biocarbonates (see Ren et al., 2009; Straub et al., 2013; Gillikin et al., 2017; de Winter et al., 2021). It seems like the pre-treatment method used in this study (simple ashing and acid treatment) does not allow this distinction to be made, while the difference between the sources of organic matter are likely important for the diagenesis discussion (section 4.1.1). I suggest that the authors add some discussion of their methodology in comparison to the state of the art and discuss how they think the preservation of different sources of organic matter may impact d13C and d15N measured in (sub-)fossil Nautilus specimens. Perhaps they could also give some recommendations for sample treatment that would allow future authors to capture the original isotopic composition of organic matter in Nautilus shells.

We thank the reviewer for this comment. We did not pre-treat the *Nautilus* shell in this manuscript but isolated all inter and intra carbonate organic matter either through drying or collecting on the ashed glass filters. The methods section has been expanded to note the differences between isolation of organic matter vs averaging all organic matter. Future work using compound specific nitrogen isotope analysis (Chikaraishi et al. 2011; Steffan et al. 2013) may provide further insight into the possibility of diagenetic alteration due to changes in the ratios within different amino acids and alteration of the amino acid chirality.

Line 108: "with less HCl" please provide the amount and concentration of the acid such that the experiments can be reproduced.

Rephrased to smaller volumes of 1 N HCl. For the cenote samples with less organic matter, more carbonate needed to be dissolved and therefore a larger volume of acid was used for complete dissolution.

Line 120-121: Only one international standard was used to correct the carbonate isotope composition. This procedure does not allow testing of potential scale compression by the mass spectrometer, which influences the accuracy of the measurements without affecting the reproducibility (precision) measured on the independent standard. If the authors have data on (an) additional independent standard(s) measured in the same routine, I suggest they add this data to show that this scale compression effect is not an issue.

Two standards were used for carbonate carbon and oxygen isotope compositions, the international NBS-18 standard, and the internal Carrera Lago Marble (CLM). The methods have been elaborated on to state that we use a two-point calibration for these measurements using NBS-18 and CLM and that additional standards NBS-19 and IAEA-603 were used to calibrate CLM. These methods were done to correct for scale compression.

Line 131: "Standard measurements suggest an instrumental precision of ±5% (RSD)." Please mention which elements were measured here. It is not clear for which element(s) this precision is reported. It is highly unlikely that all measured elements have the same measurement precision. The authors should add more detailed statistics of accuracy and precision for all measured elements here in the main text.

We have added a list of elements to the text (Ca, Mg, Mn, Na, Sr) and have also clarified the relative standard deviation. The relative standard deviation is related to the measured concentration and therefore scales with the concentration in solution. This does not mean that the precision is the same in ppm for all elements. We have also added RSD estimates for these shells.

Line 157-158: The modern shell (2000 CE) also has higher Sr/Ca and lower Mg/Ca values than the subfossil ones, but this is not really discussed in the manuscript. On the contrary, the authors make the case that the higher Sr/Ca and lower Mg/Ca values in the cenote sample are a consequence of diagenetic alteration (see section 4.1.2), but this does not explain why the modern shell also has higher Sr/Ca and lower Mg/Ca. I therefore think the paper could benefit from a more detailed discussion of the factors that may influence trace element ratios in Nautilus shells.

> We have added additional discussion about the controls on Mg/Ca and Sr/Ca in both the context of diagenesis and possible interpretation. Previous work has suggested that combined Sr/Ca and Mg/Ca is controlled by phylogeny with different *Nautilus* species falling into distinct zones. The range within the modern shell does suggest the cenote shell may not be altered and raises questions about previous work related to species-specific elemental ratios.

Line 162: "~0.12‰ lower" An assessment of the difference in isotopic value like this requires statistics. The authors should report the exact difference and the propagated error on this difference to demonstrate that it is statistically significant.

> We have added a propagation of error based on a Monte Carlo estimate from the differences using the instrumental precision for this and subsequent differences. The Student's t-test is an inappropriate test to use here for several reasons. 1) The hypothesis test with a t-test is for the means of the two populations being equal. 2) The statistical power is low at these low sample sizes (N< 2). 3) The test provides no estimate of the error on the differences between means with incorporation of instrumental error.
>
> We have added the explanation for our Monte Carlo approach to the methods section and have added the 5th to 95th percentiles of the differences between groups to the statements within the text where we compare groups of analyses.

Line 167-168: "~7‰ and ~3‰, respectively" See comment above: these comparisons should be tested statistically (e.g. using a Student's t-test) to show the reader whether the difference is significant.

> See above. These have been quantified and the 95% confidence interval from the Monte Carlo method.

Line 177: "Some have noted much lower δ15N in much older fossil nautiloids" Rephrase to "Some authors have noted…".

> Done.

Here I think a more thorough discussion of the different sources of carbon and nitrogen in the shell (e.g. carbonate-bound and non-carbonate-bound) and how they influence the d13C and d15N values is warranted. Especially since the comparison is made with very old fossil specimens.

> We have noted that our sampling combines both inter- and intra-crystalline organic matter.

Line 183-185: Here, several reasons are given for the difference in d13C between the subfossil and modern specimens. However, contrary to the d15N value, the possibility of diagenesis is not discussed as far as I can see. Can the authors be certain that diagenetic processes did not affect d13C but did affect d15N? If so, they should add some evidence to support this assumption.

> Thank you for this comment. We have added some discussion of the way that the $\delta^{13}C$ of organic matter can change with diagenesis to this section and reduced emphasis on the other mechanisms for explaining the potential cenote diagenesis.

Line 193-196: "Shell d44/44Ca… …or strontianite." I am not convinced that the authors demonstrate the correlation between calcium isotope value and Sr/Ca ratios which hints at diagenesis. The fact that the Sr/Ca values in the modern shells are highest actually contradicts this, and this is not discussed. The authors should discuss these trends in more detail if they want to convincingly demonstrate the diagenetic effect on the calcium isotopes.

> We have added discussion of the trends in Sr/Ca values in the manuscript. We have walked back the interpretation of a diagenetic effect on the calcium isotope values in the manuscript as well given the Sr/Ca range across modern specimens.

Section 4.2.2 is one of the most interesting parts of the manuscript, and the discussion here should form a nice benchmark for interpreting future cephalopod calcium isotope measurements.

> Thank you for your comment on this section.

Lines 237-239: "Culturing experiments show that the pH of extrapallial fluid from Arctica islandica bivalves is at least partially regulated during shell formation, but can vary with ambient pH (Liu et al., 2015)." The authors should explain in more detail how this fact impacts the discussion of calcium isotope variability in Nautilus shells.

We have added: If *Nautilus* similarly regulates the pH and potentially saturation state of extrapallial fluid during shell precipitation, then variation in $\Delta^{44/40}Ca_{SW-Shell}$ would be driven by fractionation during transport into the extrapallial fluid rather than with varying saturation state and precipitation rate at the site of precipitation as has been observed in inorganic systems (Lemarchand et al. 2004; Jacobson and Holmden 2008).

Line 285: "The δ13C metabolic rate proxy is more advanced in fish but holds promise for cephalopods" Here I think the authors should give a bit more background about this proxy. Most notably how it was developed and what the underlying assumptions are.

We have expanded the discussion of the development of the metabolic rate proxy. The assumptions are 1) All carbon comes from DIC or respired CO2 derived from food, 2) the aragonite enrichment factor is uniform through time, and for our calculations 3) the carbon isotope value of DIC estimation is effected by the Suess effect.

Line 296: "bulk samples" If I understood the sampling protocol correctly, these are samples from the aperture. If I'm not mistaken, this should mean that they represent the part of the shell that mineralized last. If more detailed information is known about the life cycle of Nautilus and/or their living depth at the time of depth, the authors could produce a more reliable estimate of the living depth during deposition of the sampled shell material.

A range in living depths is used here because *Nautilus* are active swimmers that cross hundreds of meters of water depth within individual days (Carlson et al. 1984; Ward et al. 1984; Dunstan et al. 2011). Shell precipitates nearly continuously across these depths (Linzmeier et al. 2016). All samples that we have from this study average days to weeks of growth across multiple depths, so any further refinement of the living depth at the time of deposition would provide false confidence given what is known about their behavior and the precipitation of their shell.

Line 300: "(~15%)" The reference to increase of the fraction of respired carbon in the shell as a percentage is slightly confusing, since this could be misread as a 15% increase relative to the original value (not an increase in absolute percentage). Perhaps this can be clarified.

Changed to reflect the change in the average Cmeta.

Line 308: Many mollusks show marked changes in d13C of their shells over their lifetimes (see e.g. McConnaughey and Gillikin, 1997 and references therein). Can such an ontogenetic trend be excluded as an explanation for the observed difference

between the samples? If so, the authors should motivate why this explanation is not considered here in the discussion.

> We have now addressed this in the discussion. Most of the $\delta^{13}C_{carb}$ change is near hatching in *Nautilus* and is not progressive near the later part of life as has been shown in bivalves.

Line 327: I am not convinced that there is more work done on diagenetic assessment in aragonites than in calcites. There exist many studies that focus on calcite alteration (e.g. Brand and Veizer, 1980; Al-Aasm and Veizer, 1986a; b; Ullmann and Korte, 2015). Perhaps this statement can be revised or clarified to better reflect the literature.

> We thank you for this comment and we have revised the document to better reflect this.

Figures 4-9: In all these figures, I would ask the authors to more clearly highlight the uncertainty on the measurements. All symbols should be equipped with error bars in horizontal and vertical direction, and ideally these error bars should reveal the same metric (preferably 2 standard deviations of reproducibility or a 95% confidence level). Even if the error bars fall within the symbol, I would still think it improves the clarity of the data if the authors would make the symbol smaller and/or represent the data points as crossover points of error bars. This way, it is more straightforward for the reader to judge whether the differences between datapoints and between groups of datapoints discussed in the text are statistically sound. In addition, if possible, it would be even better to include error ellipses (or combined error bars) of groups of datapoints that are discussed together (e.g. all sub-fossil samples or all modern specimens) on groups of specimens/measurements so the differences between groups is easier to interpret.

> We have added the error bars to the figures. We would argue that because of the low sample size in this, error ellipses are not necessarily useful for comparison between groups.

References

Al-Aasm I. S. and Veizer J. (1986a) Diagenetic stabilization of aragonite and low-Mg calcite, II. Stable isotopes in rudists. Journal of Sedimentary Research 56, 763–770.
Al-Aasm I. S. and Veizer J. (1986b) Diagenetic stabilization of aragonite and low-Mg calcite, II. Stable isotopes in rudists. Journal of Sedimentary Research 56.
Brand U. and Veizer J. (1980) Chemical diagenesis of a multicomponent carbonate system–1: Trace elements. Journal of Sedimentary Research 50, 1219–1236.
Gillikin D. P., Lorrain A., Jolivet A., Kelemen Z., Chauvaud L. and Bouillon S. (2017) High-resolution nitrogen stable isotope sclerochronology of bivalve shell carbonate-bound organics. Geochimica et Cosmochimica Acta 200, 55–66.
McConnaughey T. A., Burdett J., Whelan J. F. and Paull C. K. (1997) Carbon isotopes in biological carbonates: respiration and photosynthesis. Geochimica et Cosmochimica

Acta 61, 611–622.Ren H., Sigman D. M., Meckler A. N., Plessen B., Robinson R. S., Rosenthal Y. and Haug G. H. (2009) Foraminiferal isotope evidence of reduced nitrogen fixation in the ice age Atlantic Ocean. Science 323, 244–248.
Straub M., Sigman D. M., Ren H., Martínez-García A., Meckler A. N., Hain M. P. and Haug G. H. (2013) Changes in North Atlantic nitrogen fixation controlled by ocean circulation. Nature 501, 200–203.
Ullmann C. V. and Korte C. (2015) Diagenetic alteration in low-Mg calcite from macrofossils: a review. Geological Quarterly 59, 3–20.
de Winter N. J., Dämmer L. K., Falkenroth M., Reichart G.-J., Moretti S., Martínez-García A., Höche N., Schöne B. R., Rodiouchkina K., Goderis S., Vanhaecke F., van Leeuwen S. M. and Ziegler M. (2021) Multi-isotopic and trace element evidence against different formation pathways for oyster microstructures. Geochimica et Cosmochimica Acta 308, 326–352.

Reviewer #2: This paper lays out a clear rationale for using historical samples to assess the meaning of geochemical signals in Nautilus shells. The manuscript is very well organized, and the analytical methods used are clearly documented. However, it is difficult to gather what the meaningful lessons from this dataset are. The stated goal is to 'calibrate the geochemical response of cephalopod [analyses] to modern anthropogenic environmental change', and yet it seems the historical environmental records of these variables and the ways that the proxies respond to such forcings are both too poorly defined to achieve this goal. What are the actual hypotheses that are being tested?

> We thank the reviewer for their work to assess our manuscript. We have added to the introduction to better outline our hypotheses. We assume gradual change since the 1890's if driven by CO_2 and associated climate change and abrupt changes if ecological change driven by collapse is present. We have added this clarification to the introduction. There are limited historical records of change here, so this dataset is best interpreted in the rate and timing of change between the samples.

For example, there seems to be too many possibilities for forcing environmental d13C to know how to interpret the Nautilus signal. If the d13C signal is the result of anthropogenic changes, then why does the cenote sample fall between the historical anthropogenically-driven trend? Similarly, how can we interpret the substantial range of organic matter d13C that apparently cannot be attributed to diagenesis or diet or circulation (section 4.1.1)? There is discussion of 4-5 possible controls on d44/40Ca, but none is apparently important despite recent environmental changes because there is no change across most samples.

> One of the goals in publishing this manuscript is to greatly expand the number of $\delta^{13}C_{org}$ values available for use in calculating the percentage of metabolic carbon in the shells of extinct cephalopods (Tobin and Ward 2015; Landman et al. 2018; Ellis and Tobin 2019). We have expanded the discussion of the cenote δ^{13}Corg

and δ^{13}Ccarb and discuss how δ^{13}Corg may be changed by diagenesis. The stability of $\delta^{44/40}$Ca observed in these samples

There is a lack of quantification in some areas of the discussion. The depth range of the Nautilus habitat should be more precisely described (line 295 says average depth of 400 m – this should come earlier and include variation and range).

We have noted in the introduction, and at this location that Nautilus swims across hundreds of meters of water depth within individual days to address this point. An average living depth of 400 m is based on previous isotope analyses at New Caledonia. Being overly precise with living depth with an organism that can move as quickly as Nautilus is hasty, which is why we avoided overly precise estimates of living depth but agree that a range is important.

Line 177: 'much lower d15N' – how much lower? Comparison of Cretaceous mollusks (line 194): how much higher Sr/Ca and lower d44/40Ca in this other dataset, to aid comparison? To what degree could the cenote water composition vary from the open ocean?

We have walked back the strong conclusions about diagenetic change in the cenote specimen from the Sr/Ca and $\delta^{44/40}$Ca values. It is unclear how much different the cenote water composition could be from the open ocean.

What is the magnitude of the anthropogenic Suess effect in d13C, against which the shell d13C is compared?

The values used in the calculation are included in the discussion. The magnitude of change of $\delta^{13}C_{DIC}$ from the Suess effect at ~400 m water depth is ~0.2‰ in the last 100 years based on (Eide et al. 2017). This has a minor effect on the Cmeta contribution (e.g. calculation for modern sample without Suess effect 24.9% compared to 24.1% with the Suess effect correction).

What drives the lack of temporal change in Mg/Ca or Sr/Ca (these data are not discussed at all)?

We have expanded discussion to address this. Generally, Mg/Ca and Sr/Ca are thought to taxonomically controlled, which would explain the lack of a temporal trend. This is mentioned in the response to reviewer #1 and is expanded in the discussion.

The age axis on figures needs to be more accurate to include the 6800 year old age of the cenote sample, as well as the timing of indigenous settlement at 3000 BCE.

Given the age compression on the x axis in the figures, we decline to make this change, but instead use the broken axis style to plot the cenote, historical, and

modern datasets. The ages of samples are available in the supplemental dataset for reuse by other authors.

In summary, this paper needs to outline its hypotheses more clearly, and more clearly and quantitatively describe what the likely known or unknown forcings are, tested against the known or unknown effects of the proxies.

We thank the reviewer for their comments and hope that our revisions have adequately addressed all the outstanding changes requested.

Reviewer #3: Linzmeier et al compared isotope data from modern, historical and subfossil Nautilus shells. A very useful set of data, worth to be published in PLoS One after some moderate changes.

The Introduction needs to be more specific on the goals of the study; testable hypotheses and research questions need to be provided. In the Discussion, these questions need to be picked up and answered.

We have expanded the last paragraph of the introduction to expand the hypotheses tested here. Briefly, we suggest that gradual change since the 1890's may be driven by change in pCO_2 and abrupt change earlier is either ecological or diagenetic. Gradual or abrupt change is best interpretation.

Authors need to provide more analytical details (eg L114, L128-131 etc.).

We have added more analytical details for the isotope and elemental analyses throughout the manuscript.

The NBS-18 ref.mat is far away from the isotope values of the measured shells. It requires a two-point calibration with another, much higher standard, or more simply, you should have used a reference material closer to the samples, i.e., IAEA 603. I guess it is now too late to do that. Can you provide results from other certified materials (blindly measured ref.mats for quality control) or material with known isotope composition that have likewise been calibrated against NBS-18 in the past (or in the meantime) documenting the accuracy of the data?

We have revised our methods section to better communicate the two-point calibration done with the dataset using NBS-18 and the internal reference material. This was not done with a single standard for calibration.

What do you mean with "precision", internal precision or external precision (= accuracy)?

We mean internal precision or how well we can get the same measurement on the same material from the same instrument. This has been clarified in the text.

I am not happy with the style of the figures. Symbols are too big, color gradient of scale is superfluous, x- and y-axis must be black lines, grey background and white lines need to be removed. Figure captions need to provide details on meaning of error bars.

We have altered the style of the figures to use a discrete color palate, reduced the size of symbols, altered the color of the x- and y-axis, and removed the grey background. Figure captions all state that the error bars are 2 standard deviation instrumental precision.

Further comments are provided in the annotated pdf (copied and responded to, below).

Line 47: How separated from intra-crystalline?

Not done in this study. Changed to shell.

Line 48 – 50: in bivalve on average only 10% respired C in shell organics, though extreme cases of 40% were reported. Thus, I would not say "reflect", but is somewhat influenced by it.

Changed.

Line 57: Needs a better transition from the last paragraph.

Changed.

Line 67: New paragraph

Done.

Line 67: Goals / hypotheses / research questions?

We have adjusted this to make the research questions more apparent with a focus on abrupt vs gradual change related to differing forcings.

Line 82: perhaps, better 'sacrification'

Done, although euthanized is probably better usage, but both are commonly used.

Line 84: sacrificed

Done.

Line 114: needs more information here (not just the reference): material, isotope values

More information has been added here.

Line 120: But this is far away from your Carrara (I guess that is what you meant, not "Carrera") marble and shell values. Here, a two point calibration would be needed using an additional certified material with much higher values. Is there a chance to provide results from blindly measured reference materials close to the values of your samples (e.g., IAEA-603 or NBS-19)?

Methods have been revised to reflect the fact that we used a two-point calibration with Carrara marble and NBS-18 rather than a single point calibration.

Line 125: vol%, wt%, mol%? Please specify.

These are wt%. Values have been specified.

Line 126: any remains (filter cake?)

Material may have remained on the filter (organic matter in solution) but this was not measured in this study.

Line 128: More analytical details, please. mL of solution injected, which type of nebulizer, carrier gas; which elements have been measured, which standards used, provide RSD for each element separately. Drift during measurements corrected, how? matrix matching of samples and standards, how done or why not? additional spike used to monitor stability? etc.

We have added information on this in the methods section. Three replicates of 3 mL of solution were used. Argon was the carrier gas.

An additional spike was not used to monitor stability, but during the $\delta^{44/40}Ca$ measurement, the concentration of Ca within the original solution was checked using the double-spike method and was within 5% of the concentration measured with the ICP-OES.

Line 144: amps?

The prepared filaments are heated with an electrical current, so the current value (in amps) is provided here. In TIMS, filaments are heated in the mass spectrometer with amps.

Line 151: period means something recurring, are you sure this is what you would like to express? In my opinion L151 can be deleted.

We have slightly adjusted the wording of this sentence to not use period and choose to keep it in because we are reporting the long-term stability of measurement of a secondary standard.

Line 157: Consistently use same number of decimals for element-to-Ca values, here 3.0. Check and correct throughout ms.

Adjusted here and checked throughout.

Line 164: Likewise, consistently use same number of decimals for all isotope values, here 0.50. Check and correct throughout ms.

We checked for within isotope value consistency (e.g. $\delta^{44/40}$Ca and δ^{18}O) but disagree that all should have the same significant digits because the analytical precision is much different between the different isotope systems measured. For instance, the $\delta^{44/40}$Ca has a 2SD precision of 0.04‰ and the δ^{18}O measurements have a 2SD precision of 0.2‰.

Line 175: But there is more soluble organics than amino acids in biominerals than contain N and C, e.g., glycoproteins and sugars.

Generalized to “organic matter” and added “(e.g. amino acids, glycoproteins, sugars)” to be more precise with respect to this section.

Line 176: I guess you need to use a more scientific term here.

What exactly makes it prone for removal or resistant against loss? References would be great. For d15N you could cite Misarti et al. RCMS 31, 881-...

The term “recalcitrant organic matter” is widely used to describe organic molecules that do not easily decay.

Thank you for sharing (Misarti et al. 2017), we have revised the discussion to address points raised in that manuscript.

Line 177: Some what?

Rephrased to “Some authors”.

Line 178: Refers to what?

Low δ^{15}N values has been added here.

Line 183: abhorrent specify in which direction

Adjusted to state “lower than the modern.”

Line 195: I am sure there are other papers that could be cited and describe altered patches with otherwise well-preserved material.

Line 198: explain briefly why

This has been addressed. The location of the cenote and the radiocarbon ages for the samples point to this low temperature and age.

Line 213: macromphalus misspelled

Fixed.

Line 219: abbreviate genus name

Abbreviations done throughout the manuscript.

Line 689: coastal zone <- lower case

Fixed.

Line 722: Please also check: Mitchell, P. R. and Phakey, P. P. (1995) "Notes on the Microstructure of the Nautilus Shell," Scanning Microscopy: Vol. 9 : No. 1 , Article 16.

Line 742: Meaning of error bars?

Error bars are 2 standard deviation instrumental precision based on replicate analyses of standards for all isotope analyses and 5% RSD/5% RSD for elemental ratios. This has been noted in the figure captions.

Line 742: You provided isotope values (= delta values), not isotope ratios per se. Please correct throughout ms.

Changed throughout the manuscript.

Fig. 1: Insufficient picture quality, too small. Even the tiff was not good enough.

We are uncertain about what additional information the reviewer would like to see in this figure. We can replace the inset image with a line drawing showing the prismatic and nacreous layer schematics, but what they would like is unclear from this comment. The magnification of the shell wall is low, so nacre tablets will not be visible.

Fig. 2: lat/lon missing

Added to the redrafted map.

Fig. 4: What's the purpose of using color gradients if the symbols only use four distinct colors?

There are 8 different ages for the Nautilus samples, we are now using another discrete color gradient to differentiate the samples rather than the continuous scale used previously.

I find the grey background and white vertical and horizontal lines distracting and not useful. Please use black lines on axes.

We have changed this to a white background without. Symbols now use only 4 color levels.

Fig. 6: The symbols are not distinguishable. I find the symbols in general too large, here and in the remaining diagrams.

Symbol size has been reduced and error bars have been added to all figures.

Work Cited

Carlson, B., J. N. McKibben, and M. V. deGruy. 1984: Telemetric Investigation of Vertical Migration of *Nautilus belauensis* in Palau. Pacific Science 38:183–188.

Chikaraishi, Y., N. O. Ogawa, H. Doi, and N. Ohkouchi. 2011: $^{15}N/^{14}N$ ratios of amino acids as a tool for studying terrestrial food webs: a case study of terrestrial insects (bees, wasps, and hornets). Ecological Research 26:835–844.

Dunstan, A. J., P. D. Ward, and N. J. Marshall. 2011: Vertical distribution and migration patterns of *Nautilus pompilius*. PLoS ONE 6:e16311.

Eide, M., A. Olsen, U. S. Ninnemann, and T. Eldevik. 2017: A global estimate of the full oceanic 13C Suess effect since the preindustrial. Global Biogeochemical Cycles 31:492–514.

Ellis, N. M., and T. S. Tobin. 2019: Evidence for seasonal variation in $\delta^{13}C$ and $\delta^{18}O$ profiles of *Baculites* and implications for growth rate. Palaeontology 62:583–598.

Jacobson, A. D., and C. Holmden. 2008: $\delta^{44}Ca$ evolution in a carbonate aquifer and its bearing on the equilibrium isotope fractionation factor for calcite. Earth and Planetary Science Letters 270:349–353.

Landman, N. H., J. K. Cochran, M. Slovacek, N. L. Larson, M. P. Garb, J. Brezina, and J. D. Witts. 2018: Isotope sclerochronology of ammonites (*Baculites compressus*) from methane seep and non-seep sites in the Late Cretaceous Western Interior Seaway, USA: Implications for ammonite habitat and mode of life. American Journal of Science 318:603–639.

Lemarchand, D., G. J. Wasserburg, and D. A. Papanastassiou. 2004: Rate-controlled calcium isotope fractionation in synthetic calcite. Geochimica et Cosmochimica Acta 68:4665–4678.

Linzmeier, B. J., R. Kozdon, S. E. Peters, and J. W. Valley. 2016: Oxygen isotope variability within *Nautilus* shell growth bands. PLOS ONE 11:e0153890.

Misarti, N., E. Gier, B. Finney, K. Barnes, and M. McCarthy. 2017: Compound-specific amino acid $\delta^{15}N$ values in archaeological shell: Assessing diagenetic integrity and potential for isotopic baseline reconstruction. Rapid Communications in Mass Spectrometry 31:1881–1891.

Steffan, S. A., Y. Chikaraishi, D. R. Horton, N. Ohkouchi, M. E. Singleton, E. Miliczky, D. B. Hogg, and V. P. Jones. 2013: Trophic Hierarchies Illuminated via Amino Acid Isotopic Analysis. PLoS ONE 8:e76152.

Tobin, T. S., and P. D. Ward. 2015: Carbon isotope ($\delta^{13}C$) differences between Late Cretaceous ammonites and benthic mollusks from Antarctica. Palaeogeography, Palaeoclimatology, Palaeoecology 428:50–57.

Ward, P. D., B. Carlson, M. Weekly, and B. Brumbaugh. 1984: Remote telemetry of daily vertical and horizontal movement of *Nautilus* in Palau. Nature 309:248–250.
