# Supplementary material for: Isotope systematics of subfossil, historical, and modern Nautilus macromphalus from New Caledonia
Source: PLoS One. 2022 Dec 28;17(12):e0277666. doi: 10.1371/journal.pone.0277666 (PMC9797077; doi:10.1371/journal.pone.0277666)
Supplement: Attachment — Submitted filename: Response to Reviewers.docx [file pone.0277666.s003.docx]

*Reviewer #1: Dear authors,

In their manuscript, Linzmeier et al. present a multi-proxy dataset measured in modern and sub-fossil Nautilus macromphalus shell carbonate. The goal of the study is to investigate the effect of several environmental parameters on the shell composition of N. macromphalus to provide a baseline for studies on historical and fossil specimens of this and related species aiming to reconstruct environmental change. The authors present a valuable dataset of natural variability as recorded in Nautilus shells which complements previous culture experiments. The decision to work on shells from historical collection is interesting, as it avoids certain complications of culture experiments (most notably the shock to the animals due to changing aquarium conditions) as well as the ethical considerations of experimenting on live cephalopods. Overall, I believe this manuscript merits publication barring some moderate revisions, mostly pertaining to the methodology and the presentation of the data, which I will highlight in my line-by-line comments below:*

We thank the reviewer for their work on our manuscript. We have carefully addressed your comments in the document below and in the revised manuscript.

*Line 42: “isotope systematics of light stable isotopes” rephrase to “**systematics of light stable isotopes”*

Done.

*Line 92: The authors should provide the uncertainty on the radiocarbon date of the cenote specimen in the text*

Done.

*Lines 104-115: Recently, there has been some discussion in the literature about the pre-treatment methods required to distinguish between the contributions of skeletal carbonate-bound organic matter and non-carbonate bound organic matter phases in d13C and d15N analyses of biocarbonates (see Ren et al., 2009; Straub et al., 2013; Gillikin et al., 2017; de Winter et al., 2021). It seems like the pre-treatment method used in this study (simple ashing and acid treatment) does not allow this distinction to be made, while the difference between the sources of organic matter are likely important for the diagenesis discussion (section 4.1.1). I suggest that the authors add some discussion of their methodology in comparison to the state of the art and discuss how they think the preservation of different sources of organic matter may impact d13C and d15N measured in (sub-)fossil Nautilus specimens. Perhaps they could also give some recommendations for sample treatment that would allow future authors to capture the original isotopic composition of organic matter in Nautilus shells.*

We thank the reviewer for this comment. We did not pre-treat the *Nautilus* shell in this manuscript but isolated all inter and intra carbonate organic matter either through drying or collecting on the ashed glass filters. The methods section has been expanded to note the differences between isolation of organic matter vs averaging all organic matter. Future work using compound specific nitrogen isotope analysis (Chikaraishi et al. 2011; Steffan et al. 2013) may provide further insight into the possibility of diagenetic alteration due to changes in the ratios within different amino acids and alteration of the amino acid chirality.

*Line 108: “with less HCl” please provide the amount and concentration of the acid such that the experiments can be reproduced.*

Rephrased to smaller volumes of 1 N HCl. For the cenote samples with less organic matter, more carbonate needed to be dissolved and therefore a larger volume of acid was used for complete dissolution.

*Line 120-121: Only one international standard was used to correct the carbonate isotope composition. This procedure does not allow testing of potential scale compression by the mass spectrometer, which influences the accuracy of the measurements without affecting the reproducibility (precision) measured on the independent standard. If the authors have data on (an) additional independent standard(s) measured in the same routine, I suggest they add this data to show that this scale compression effect is not an issue.*

Two standards were used for carbonate carbon and oxygen isotope compositions, the international NBS-18 standard, and the internal Carrera Lago Marble (CLM). The methods have been elaborated on to state that we use a two-point calibration for these measurements using NBS-18 and CLM and that additional standards NBS-19 and IAEA-603 were used to calibrate CLM. These methods were done to correct for scale compression.

*Line 131: “Standard measurements suggest an instrumental precision of ±5% (RSD).” Please mention which elements were measured here. It is not clear for which element(s) this precision is reported. It is highly unlikely that all measured elements have the same measurement precision. The authors should add more detailed statistics of accuracy and precision for all measured elements here in the main text.*

We have added a list of elements to the text (Ca, Mg, Mn, Na, Sr) and have also clarified the relative standard deviation. The relative standard deviation is related to the measured concentration and therefore scales with the concentration in solution. This does not mean that the precision is the same in ppm for all elements. We have also added RSD estimates for these shells.

*Line 157-158: The modern shell (2000 CE) also has higher Sr/Ca and lower Mg/Ca values than the subfossil ones, but this is not really discussed in the manuscript. On the contrary, the authors make the case that the higher Sr/Ca and lower Mg/Ca values in the cenote sample are a consequence of diagenetic alteration (see section 4.1.2), but this does not explain why the modern shell also has higher Sr/Ca and lower Mg/Ca. I therefore think the paper could benefit from a more detailed discussion of the factors that may influence trace element ratios in Nautilus shells.*

We have added additional discussion about the controls on Mg/Ca and Sr/Ca in both the context of diagenesis and possible interpretation. Previous work has suggested that combined Sr/Ca and Mg/Ca is controlled by phylogeny with different *Nautilus* species falling into distinct zones. The range within the modern shell does suggest the cenote shell may not be altered and raises questions about previous work related to species-specific elemental ratios.

*Line 162: “~0.12‰ lower” An assessment of the difference in isotopic value like this requires statistics. The authors should report the exact difference and the propagated error on this difference to demonstrate that it is statistically significant.*

We have added a propagation of error based on a Monte Carlo estimate from the differences using the instrumental precision for this and subsequent differences. The Student’s t-test is an inappropriate test to use here for several reasons. 1) The hypothesis test with a t-test is for the means of the two populations being equal. 2) The statistical power is low at these low sample sizes (N< 2). 3) The test provides no estimate of the error on the differences between means with incorporation of instrumental error.

We have added the explanation for our Monte Carlo approach to the methods section and have added the 5^th^ to 95^th^ percentiles of the differences between groups to the statements within the text where we compare groups of analyses.

*Line 167-168: “~7‰ and ~3‰, respectively” See comment above: these comparisons should be tested statistically (e.g. using a Student’s t-test) to show the reader whether the difference is significant.*

See above. These have been quantified and the 95% confidence interval from the Monte Carlo method.

*Line 177: “Some have noted much lower δ15N in much older fossil nautiloids” Rephrase to “Some authors have noted…”.*

Done.

*Here I think a more thorough discussion of the different sources of carbon and nitrogen in the shell (e.g. carbonate-bound and non-carbonate-bound) and how they influence the d13C and d15N values is warranted. Especially since the comparison is made with very old fossil specimens.*

We have noted that our sampling combines both inter- and intra-crystalline organic matter.

*Line 183-185: Here, several reasons are given for the difference in d13C between the subfossil and modern specimens. However, contrary to the d15N value, the possibility of diagenesis is not discussed as far as I can see. Can the authors be certain that diagenetic processes did not affect d13C but did affect d15N? If so, they should add some evidence to support this assumption.*

Thank you for this comment. We have added some discussion of the way that the δ^13^C of organic matter can change with diagenesis to this section and reduced emphasis on the other mechanisms for explaining the potential cenote diagenesis.

*Line 193-196: “Shell d44/44Ca… …or strontianite.” I am not convinced that the authors demonstrate the correlation between calcium isotope value and Sr/Ca ratios which hints at diagenesis. The fact that the Sr/Ca values in the modern shells are highest actually contradicts this, and this is not discussed. The authors should discuss these trends in more detail if they want to convincingly demonstrate the diagenetic effect on the calcium isotopes.*

We have added discussion of the trends in Sr/Ca values in the manuscript. We have walked back the interpretation of a diagenetic effect on the calcium isotope values in the manuscript as well given the Sr/Ca range across modern specimens.

*Section 4.2.2 is one of the most interesting parts of the manuscript, and the discussion here should form a nice benchmark for interpreting future cephalopod calcium isotope measurements.*

Thank you for your comment on this section.

*Lines 237-239: “Culturing experiments show that the pH of extrapallial fluid from Arctica islandica bivalves is at least partially regulated during shell formation, but can vary with ambient pH (Liu et al., 2015).” The authors should explain in more detail how this fact impacts the discussion of calcium isotope variability in Nautilus shells.*

We have added: If *Nautilus* similarly regulates the pH and potentially saturation state of extrapallial fluid during shell precipitation, then variation in Δ^44/40^Ca_SW-Shell_ would be driven by fractionation during transport into the extrapallial fluid rather than with varying saturation state and precipitation rate at the site of precipitation as has been observed in inorganic systems (Lemarchand et al. 2004; Jacobson and Holmden 2008).

*Line 285: “The δ13C metabolic rate proxy is more advanced in fish but holds promise for cephalopods” Here I think the authors should give a bit more background about this proxy. Most notably how it was developed and what the underlying assumptions are.*

We have expanded the discussion of the development of the metabolic rate proxy. The assumptions are 1) All carbon comes from DIC or respired CO2 derived from food, 2) the aragonite enrichment factor is uniform through time, and for our calculations 3) the carbon isotope value of DIC estimation is effected by the Suess effect.

*Line 296: “bulk samples” If I understood the sampling protocol correctly, these are samples from the aperture. If I’m not mistaken, this should mean that they represent the part of the shell that mineralized last. If more detailed information is known about the life cycle of Nautilus and/or their living depth at the time of depth, the authors could produce a more reliable estimate of the living depth during deposition of the sampled shell material.*

A range in living depths is used here because *Nautilus* are active swimmers that cross hundreds of meters of water depth within individual days (Carlson et al. 1984; Ward et al. 1984; Dunstan et al. 2011). Shell precipitates nearly continuously across these depths (Linzmeier et al. 2016). All samples that we have from this study average days to weeks of growth across multiple depths, so any further refinement of the living depth at the time of deposition would provide false confidence given what is known about their behavior and the precipitation of their shell.

*Line 300: “(~15%)” The reference to increase of the fraction of respired carbon in the shell as a percentage is slightly confusing, since this could be misread as a 15% increase relative to the original value (not an increase in absolute percentage). Perhaps this can be clarified.*

Changed to reflect the change in the average Cmeta.

*Line 308: Many mollusks show marked changes in d13C of their shells over their lifetimes (see e.g. McConnaughey and Gillikin, 1997 and references therein). Can such an ontogenetic trend be excluded as an explanation for the observed difference between the samples? If so, the authors should motivate why this explanation is not considered here in the discussion.*

We have now addressed this in the discussion. Most of the δ^13^C_carb_ change is near hatching in *Nautilus* and is not progressive near the later part of life as has been shown in bivalves.

*Line 327: I am not convinced that there is more work done on diagenetic assessment in aragonites than in calcites. There exist many studies that focus on calcite alteration (e.g. Brand and Veizer, 1980; Al-Aasm and Veizer, 1986a; b; Ullmann and Korte, 2015). Perhaps this statement can be revised or clarified to better reflect the literature.*

We thank you for this comment and we have revised the document to better reflect this.

*Figures 4-9: In all these figures, I would ask the authors to more clearly highlight the uncertainty on the measurements. All symbols should be equipped with error bars in horizontal and vertical direction, and ideally these error bars should reveal the same metric (preferably 2 standard deviations of reproducibility or a 95% confidence level). Even if the error bars fall within the symbol, I would still think it improves the clarity of the data if the authors would make the symbol smaller and/or represent the data points as crossover points of error bars. This way, it is more straightforward for the reader to judge whether the differences between datapoints and between groups of datapoints discussed in the text are statistically sound. In addition, if possible, it would be even better to include error ellipses (or combined error bars) of groups of datapoints that are discussed together (e.g. all sub-fossil samples or all modern specimens) on groups of specimens/measurements so the differences between groups is easier to interpret.*

We have added the error bars to the figures. We would argue that because of the low sample size in this, error ellipses are not necessarily useful for comparison between groups.

References
Al-Aasm I. S. and Veizer J. (1986a) Diagenetic stabilization of aragonite and low-Mg calcite, II. Stable isotopes in rudists. Journal of Sedimentary Research 56, 763–770.
Al-Aasm I. S. and Veizer J. (1986b) Diagenetic stabilization of aragonite and low-Mg calcite, II. Stable isotopes in rudists. Journal of Sedimentary Research 56.
Brand U. and Veizer J. (1980) Chemical diagenesis of a multicomponent carbonate system–1: Trace elements. Journal of Sedimentary Research 50, 1219–1236.
Gillikin D. P., Lorrain A., Jolivet A., Kelemen Z., Chauvaud L. and Bouillon S. (2017) High-resolution nitrogen stable isotope sclerochronology of bivalve shell carbonate-bound organics. Geochimica et Cosmochimica Acta 200, 55–66.
McConnaughey T. A., Burdett J., Whelan J. F. and Paull C. K. (1997) Carbon isotopes in biological carbonates: respiration and photosynthesis. Geochimica et Cosmochimica Acta 61, 611–622.Ren H., Sigman D. M., Meckler A. N., Plessen B., Robinson R. S., Rosenthal Y. and Haug G. H. (2009) Foraminiferal isotope evidence of reduced nitrogen fixation in the ice age Atlantic Ocean. Science 323, 244–248.
Straub M., Sigman D. M., Ren H., Martínez-García A., Meckler A. N., Hain M. P. and Haug G. H. (2013) Changes in North Atlantic nitrogen fixation controlled by ocean circulation. Nature 501, 200–203.
Ullmann C. V. and Korte C. (2015) Diagenetic alteration in low-Mg calcite from macrofossils: a review. Geological Quarterly 59, 3–20.
de Winter N. J., Dämmer L. K., Falkenroth M., Reichart G.-J., Moretti S., Martínez-García A., Höche N., Schöne B. R., Rodiouchkina K., Goderis S., Vanhaecke F., van Leeuwen S. M. and Ziegler M. (2021) Multi-isotopic and trace element evidence against different formation pathways for oyster microstructures. Geochimica et Cosmochimica Acta 308, 326–352.

*Reviewer #2: This paper lays out a clear rationale for using historical samples to assess the meaning of geochemical signals in Nautilus shells. The manuscript is very well organized, and the analytical methods used are clearly documented. However, it is difficult to gather what the meaningful lessons from this dataset are. The stated goal is to ‘calibrate the geochemical response of cephalopod [analyses] to modern anthropogenic environmental change’, and yet it seems the historical environmental records of these variables and the ways that the proxies respond to such forcings are both too poorly defined to achieve this goal. What are the actual hypotheses that are being tested?*

We thank the reviewer for their work to assess our manuscript. We have added to the introduction to better outline our hypotheses. We assume gradual change since the 1890’s if driven by CO_2_ and associated climate change and abrupt changes if ecological change driven by collapse is present. We have added this clarification to the introduction. There are limited historical records of change here, so this dataset is best interpreted in the rate and timing of change between the samples.

*For example, there seems to be too many possibilities for forcing environmental d13C to know how to interpret the Nautilus signal. If the d13C signal is the result of anthropogenic changes, then why does the cenote sample fall between the historical anthropogenically-driven trend? Similarly, how can we interpret the substantial range of organic matter d13C that apparently cannot be attributed to diagenesis or diet or circulation (section 4.1.1)? There is discussion of 4-5 possible controls on d44/40Ca, but none is apparently important despite recent environmental changes because there is no change across most samples.*

One of the goals in publishing this manuscript is to greatly expand the number of δ^13^C_org_ values available for use in calculating the percentage of metabolic carbon in the shells of extinct cephalopods (Tobin and Ward 2015; Landman et al. 2018; Ellis and Tobin 2019). We have expanded the discussion of the cenote δ^13^Corg and δ^13^Ccarb and discuss how δ^13^Corg may be changed by diagenesis. The stability of δ^44/40^Ca observed in these samples

*There is a lack of quantification in some areas of the discussion. The depth range of the Nautilus habitat should be more precisely described (line 295 says average depth of 400 m – this should come earlier and include variation and range).*

We have noted in the introduction, and at this location that Nautilus swims across hundreds of meters of water depth within individual days to address this point. An average living depth of 400 m is based on previous isotope analyses at New Caledonia. Being overly precise with living depth with an organism that can move as quickly as Nautilus is hasty, which is why we avoided overly precise estimates of living depth but agree that a range is important.

*Line 177: ‘much lower d15N’ – how much lower? Comparison of Cretaceous mollusks (line 194): how much higher Sr/Ca and lower d44/40Ca in this other dataset, to aid comparison? To what degree could the cenote water composition vary from the open ocean?*

We have walked back the strong conclusions about diagenetic change in the cenote specimen from the Sr/Ca and δ^44/40^Ca values. It is unclear how much different the cenote water composition could be from the open ocean.

*What is the magnitude of the anthropogenic Suess effect in d13C, against which the shell d13C is compared?*

The values used in the calculation are included in the discussion. The magnitude of change of δ^13^C_DIC_ from the Suess effect at ~400 m water depth is ~0.2‰ in the last 100 years based on (Eide et al. 2017). This has a minor effect on the Cmeta contribution (e.g. calculation for modern sample without Suess effect 24.9% compared to 24.1% with the Suess effect correction).

*What drives the lack of temporal change in Mg/Ca or Sr/Ca (these data are not discussed at all)?*

We have expanded discussion to address this. Generally, Mg/Ca and Sr/Ca are thought to taxonomically controlled, which would explain the lack of a temporal trend. This is mentioned in the response to reviewer #1 and is expanded in the discussion.

*The age axis on figures needs to be more accurate to include the 6800 year old age of the cenote sample, as well as the timing of indigenous settlement at 3000 BCE.*

Given the age compression on the x axis in the figures, we decline to make this change, but instead use the broken axis style to plot the cenote, historical, and modern datasets. The ages of samples are available in the supplemental dataset for reuse by other authors.

*In summary, this paper needs to outline its hypotheses more clearly, and more clearly and quantitatively describe what the likely known or unknown forcings are, tested against the known or unknown effects of the proxies.*

We thank the reviewer for their comments and hope that our revisions have adequately addressed all the outstanding changes requested.

*Reviewer #3: Linzmeier et al compared isotope data from modern, historical and subfossil Nautilus shells. A very useful set of data, worth to be published in PLoS One after some moderate changes.

The Introduction needs to be more specific on the goals of the study; testable hypotheses and research questions need to be provided. In the Discussion, these questions need to be picked up and answered.*

We have expanded the last paragraph of the introduction to expand the hypotheses tested here. Briefly, we suggest that gradual change since the 1890’s may be driven by change in *p*CO_2_ and abrupt change earlier is either ecological or diagenetic. Gradual or abrupt change is best interpretation.

*Authors need to provide more analytical details (eg L114, L128-131 etc.).*

We have added more analytical details for the isotope and elemental analyses throughout the manuscript.

*The NBS-18 ref.mat is far away from the isotope values of the measured shells. It requires a two-point calibration with another, much higher standard, or more simply, you should have used a reference material closer to the samples, i.e., IAEA 603. I guess it is now too late to do that. Can you provide results from other certified materials (blindly measured ref.mats for quality control) or material with known isotope composition that have likewise been calibrated against NBS-18 in the past (or in the meantime) documenting the accuracy of the data?*

We have revised our methods section to better communicate the two-point calibration done with the dataset using NBS-18 and the internal reference material. This was not done with a single standard for calibration.

*What do you mean with "precision", internal precision or external precision (= accuracy)?*

We mean internal precision or how well we can get the same measurement on the same material from the same instrument. This has been clarified in the text.

*I am not happy with the style of the figures. Symbols are too big, color gradient of scale is superfluous, x- and y-axis must be black lines, grey background and white lines need to be removed. Figure captions need to provide details on meaning of error bars.*

We have altered the style of the figures to use a discrete color palate, reduced the size of symbols, altered the color of the x- and y-axis, and removed the grey background. Figure captions all state that the error bars are 2 standard deviation instrumental precision.

***Further comments are provided in the annotated pdf (copied and responded to, below).***

*Line 47: How separated from intra-crystalline?*

Not done in this study. Changed to shell.

*Line 48 – 50: in bivalve on average only 10% respired C in shell organics, though extreme cases of 40% were reported. Thus, I would not say "reflect", but is somewhat influenced by it.*

Changed.

*Line 57: Needs a better transition from the last paragraph.*

Changed.

*Line 67: New paragraph*

Done.

*Line 67: Goals / hypotheses / research questions?*

We have adjusted this to make the research questions more apparent with a focus on abrupt vs gradual change related to differing forcings.

*Line 82: perhaps, better 'sacrification'*

Done, although euthanized is probably better usage, but both are commonly used.

*Line 84: sacrificed*

Done.

*Line 114:* *needs more information here (not just the reference): material, isotope values*

More information has been added here.

*Line 120: But this is far away from your Carrara (I guess that is what you meant, not "Carrera") marble and shell values. Here, a two point calibration would be needed using an additional certified material with much higher values. Is there a chance to provide results from blindly measured reference materials close to the values of your samples (e.g., IAEA-603 or NBS-19)?*

Methods have been revised to reflect the fact that we used a two-point calibration with Carrara marble and NBS-18 rather than a single point calibration.

*Line 125: vol%, wt%, mol%? Please specify.*

These are wt%. Values have been specified.

*Line 126: any remains (filter cake?)*

Material may have remained on the filter (organic matter in solution) but this was not measured in this study.

*Line 128:* *More analytical details, please. mL of solution injected, which type of nebulizer, carrier gas; which elements have been measured, which standards used, provide RSD for each element separately. Drift during measurements corrected, how? matrix matching of samples and standards, how done or why not? additional spike used to monitor stability? etc.*

We have added information on this in the methods section. Three replicates of 3 mL of solution were used. Argon was the carrier gas.

An additional spike was not used to monitor stability, but during the δ^44/40^Ca measurement, the concentration of Ca within the original solution was checked using the double-spike method and was within 5% of the concentration measured with the ICP-OES.

*Line 144: amps?*

The prepared filaments are heated with an electrical current, so the current value (in amps) is provided here. In TIMS, filaments are heated in the mass spectrometer with amps.

*Line 151: period means something recurring, are you sure this is what you would like to express? In my opinion L151 can be deleted.*

We have slightly adjusted the wording of this sentence to not use period and choose to keep it in because we are reporting the long-term stability of measurement of a secondary standard.

*Line 157: Consistently use same number of decimals for element-to-Ca values, here 3.0. Check and correct throughout ms.*

Adjusted here and checked throughout.

*Line 164: Likewise, consistently use same number of decimals for all isotope values, here 0.50. Check and correct throughout ms.*

We checked for within isotope value consistency (e.g. δ^44/40^Ca and δ^18^O) but disagree that all should have the same significant digits because the analytical precision is much different between the different isotope systems measured. For instance, the δ^44/40^Ca has a 2SD precision of 0.04‰ and the δ^18^O measurements have a 2SD precision of 0.2‰.

*Line 175: But there is more soluble organics than amino acids in biominerals than contain N and C, e.g., glycoproteins and sugars.*

Generalized to “organic matter” and added “(e.g. amino acids, glycoproteins, sugars)” to be more precise with respect to this section.

*Line 176: I guess you need to use a more scientific term here.*

*What exactly makes it prone for removal or resistant against loss? References would be great. For d15N you could cite Misarti et al. RCMS 31, 881-...*

The term “recalcitrant organic matter” is widely used to describe organic molecules that do not easily decay.

Thank you for sharing (Misarti et al. 2017), we have revised the discussion to address points raised in that manuscript.

*Line 177: Some what?*

Rephrased to “Some authors”.

*Line 178: Refers to what?*

Low δ^15^N values has been added here.

*Line 183: abhorrent specify in which direction*

Adjusted to state “lower than the modern.”

*Line 195: I am sure there are other papers that could be cited and describe altered patches with otherwise well-preserved material.*

*Line 198: explain briefly why*

This has been addressed. The location of the cenote and the radiocarbon ages for the samples point to this low temperature and age.

*Line 213: macromphalus misspelled*

Fixed.

*Line 219: abbreviate genus name*

Abbreviations done throughout the manuscript.

*Line 689: coastal zone <- lower case*

Fixed.

*Line 722: Please also check: Mitchell, P. R. and Phakey, P. P. (1995) “Notes on the Microstructure of the Nautilus Shell,” Scanning Microscopy: Vol. 9 : No. 1 , Article 16.*

*Line 742: Meaning of error bars?*

Error bars are 2 standard deviation instrumental precision based on replicate analyses of standards for all isotope analyses and 5% RSD/5% RSD for elemental ratios. This has been noted in the figure captions.

*Line 742: You provided isotope values (= delta values), not isotope ratios per se. Please correct throughout ms.*

Changed throughout the manuscript.

*Fig. 1: Insufficient picture quality, too small. Even the tiff was not good enough.*

We are uncertain about what additional information the reviewer would like to see in this figure. We can replace the inset image with a line drawing showing the prismatic and nacreous layer schematics, but what they would like is unclear from this comment. The magnification of the shell wall is low, so nacre tablets will not be visible.

*Fig. 2: lat/lon missing*

Added to the redrafted map.

*Fig. 4: What's the purpose of using color gradients if the symbols only use four distinct colors?*

There are 8 different ages for the Nautilus samples, we are now using another discrete color gradient to differentiate the samples rather than the continuous scale used previously.

*I find the grey background and white vertical and horizontal lines distracting and not useful. Please use black lines on axes.*

We have changed this to a white background without. Symbols now use only 4 color levels.

*Fig. 6: The symbols are not distinguishable. I find the symbols in general too large, here and in the remaining diagrams.*

Symbol size has been reduced and error bars have been added to all figures.

**Work Cited**

Carlson, B., J. N. McKibben, and M. V. deGruy. 1984: Telemetric Investigation of Vertical Migration of *Nautilus belauensis* in Palau. Pacific Science 38:183–188.

Chikaraishi, Y., N. O. Ogawa, H. Doi, and N. Ohkouchi. 2011: ^15^N/^14^N ratios of amino acids as a tool for studying terrestrial food webs: a case study of terrestrial insects (bees, wasps, and hornets). Ecological Research 26:835–844.

Dunstan, A. J., P. D. Ward, and N. J. Marshall. 2011: Vertical distribution and migration patterns of *Nautilus pompilius*. PLoS ONE 6:e16311.

Eide, M., A. Olsen, U. S. Ninnemann, and T. Eldevik. 2017: A global estimate of the full oceanic 13C Suess effect since the preindustrial. Global Biogeochemical Cycles 31:492–514.

Ellis, N. M., and T. S. Tobin. 2019: Evidence for seasonal variation in δ^13^C and δ^18^O profiles of *Baculites* and implications for growth rate. Palaeontology 62:583–598.

Jacobson, A. D., and C. Holmden. 2008: δ^44^Ca evolution in a carbonate aquifer and its bearing on the equilibrium isotope fractionation factor for calcite. Earth and Planetary Science Letters 270:349–353.

Landman, N. H., J. K. Cochran, M. Slovacek, N. L. Larson, M. P. Garb, J. Brezina, and J. D. Witts. 2018: Isotope sclerochronology of ammonites (*Baculites compressus*) from methane seep and non-seep sites in the Late Cretaceous Western Interior Seaway, USA: Implications for ammonite habitat and mode of life. American Journal of Science 318:603–639.

Lemarchand, D., G. J. Wasserburg, and D. A. Papanastassiou. 2004: Rate-controlled calcium isotope fractionation in synthetic calcite. Geochimica et Cosmochimica Acta 68:4665–4678.

Linzmeier, B. J., R. Kozdon, S. E. Peters, and J. W. Valley. 2016: Oxygen isotope variability within *Nautilus* shell growth bands. PLOS ONE 11:e0153890.

Misarti, N., E. Gier, B. Finney, K. Barnes, and M. McCarthy. 2017: Compound-specific amino acid δ^15^N values in archaeological shell: Assessing diagenetic integrity and potential for isotopic baseline reconstruction. Rapid Communications in Mass Spectrometry 31:1881–1891.

Steffan, S. A., Y. Chikaraishi, D. R. Horton, N. Ohkouchi, M. E. Singleton, E. Miliczky, D. B. Hogg, and V. P. Jones. 2013: Trophic Hierarchies Illuminated via Amino Acid Isotopic Analysis. PLoS ONE 8:e76152.

Tobin, T. S., and P. D. Ward. 2015: Carbon isotope (δ^13^C) differences between Late Cretaceous ammonites and benthic mollusks from Antarctica. Palaeogeography, Palaeoclimatology, Palaeoecology 428:50–57.

Ward, P. D., B. Carlson, M. Weekly, and B. Brumbaugh. 1984: Remote telemetry of daily vertical and horizontal movement of *Nautilus* in Palau. Nature 309:248–250.
