# Supplementary material for: Isotope systematics of subfossil, historical, and modern Nautilus macromphalus from New Caledonia
Source: PLoS One. 2022 Dec 28;17(12):e0277666. doi: 10.1371/journal.pone.0277666 (PMC9797077; doi:10.1371/journal.pone.0277666)
Supplement: Attachment — Submitted filename: Isotope systematics of Nautilus Response to Reviews 2.docx [file pone.0277666.s005.docx]

Editor Line-by-line comments:

Line 59: y

Changed

Line 64: perhaps cite the earliest study/ies

In [20], some unpublished data on *Nautilus* are presented and discussed. The reference itself is a book chapter. Since initial submission of this paper, others [21] have published a few additional δ^44/40^Ca analyses of *Nautilus* and other cephalopods.

Line 84: perhaps mention earlier that the samples are from collections

We have added a clause in the first sentence.

Line 94: in italics and N. macromphalus, as reviewer suggested

We have addressed this through the manuscript.

Line 97: red? What are the abbreviations?

Changed and notes on the abbreviations (museum codes) added.

Line 118: are these radiocarbon or calendar years?

These are radiocarbon ages reported as years before present.

Line 181: perhaps write in full: amperes, or use the standard abbreviation A

This has been changed.

Line 189: is this how the data are made publicly available? Why not make them even FAIRer and deposit them in a known data repository (pangaea.de seems a good choice for this type of work; <https://journals.plos.org/plosone/s/recommended-repositories>)

We will deposit them in Earthchem to be FAIRer with these data. Pangaea.de has a fee for curation of data and takes a bit longer (6 weeks vs 2 weeks) to curate.

Line 281: s

Added.

Line 387: the formula (and lack of unit) suggest this is a fraction or proportion, rather than quantity?

Changed to proportion.

Line 386: adaptation?

We are not stating strict adaptation because there is not selection but rather some sort of phenotypic response that may take adjustment during development to express. From Fregley, 1996): Acclimation is a phenotypic response developed by an animal to a environmental stressor.

Fregley, M. J . 1996. Adaptations: some general characteristics. In: Fregley, M. J., and C. M.Blatteis, editors. Handbook of physiology, section 4: environmental physiology. vol. I. Oxford: Oxford University Press; p. 3–15.

Reviewer #1: Dear Authors,

Thank you for your comprehensive reply to the review comments, and for submitting a thoroughly revised manuscript. In my opinion, all the queries I have posed in my review have been addressed and I appreciate the way the authors responded to the other review comments as well. The manuscript has improved significantly since the previous version and I am happy to support publication in its current form.

Thank you for your work in critiquing the first and revised versions of the manuscript.

Reviewer #2: The authors have completed a solid revision of their manuscript that presents their results in helpful and appropriate context, including possible mechanisms explaining the variation (and ruling out some that do not) and guiding the reader to remaining questions and room for further work. I think this contribution will be valuable to others seeking to perform robust calibrations of skeletal-based geochemical proxies.

Thank you for both your first review and your work to check through the revision.

Reviewer #3: Most of the comments raised in my first review have been properly addressed by Benjamin Linzmeier and colleagues.

However, the authors need to follow common rules in abbreviating taxonomic names. I did not ask to abbreviate the species name (macromphalus), but the genus name, Nautilus, after the first mention in the ms. Hence, Nautilus macromphalus becomes N. macromphalus with the second and following occurences in the main text. Genus and species names should still spelled out in the figure captions and not abbreviated.

Thank you for catching this. I’m not entirely sure why I ended up doing that style of abbreviation, but it has been changed.

It still falsely reads "Carrera"! The Italian city is spelled Carrara (with three "a"s), and so is the corresponding marble. The company Carrera produces racetracks for kids.

We are using the Carrara Marble from Italy and not the marble from General Carrera Lake in Chile/Argentina. It has been changed to the proper spelling.

I still do not understand the relevance of Figure 3C. If the authors want to show how the microstructure of the different shell layers looks like, nacreous and prismatic, they need to provide a high-resolution close-up (SEM) image that reveals the individual nacre tablets and prisms. The current figure does not provide any useful information.

We have removed it.
